# Thermodynamic screening of solid–gas CO_2_ mineral carbonation: a provenance-traceable Gibbs-energy framework across 23 reactions

**DOI:** 10.1039/d6ra07742k

**Published:** 2026-10-21

**Authors:** Abdulaziz A. Alturki

**Affiliations:** a Department of Chemical and Materials Engineering, Faculty of Engineering, King Abdulaziz University Jeddah 21589 Saudi Arabia aaaaltrki@kau.edu.sa; b Center of Research Excellence in Renewable Energy and Power Systems, King Abdulaziz University Jeddah 21589 Saudi Arabia

## Abstract

Screening solid sorbents for CO_2_ capture by the standard reaction Gibbs energy, Δ*G*°, or its turnover temperature does not show how far carbonation proceeds in a dilute gas charge. Established Gibbs-energy minimization is applied explicitly along the reaction extent *ξ* for 23 solid–gas carbonation reactions from six sorbent families. Gas-phase stoichiometry separates the reactions into three classes, whose profile shapes also depend on the initial charge. In Class I, where CO_2_ is the only gaseous participant (18 reactions), an undiluted feed fixes the gas mole fraction, makes *G* linear in *ξ* and gives binary equilibrium except at the transition; an inert diluent instead depletes CO_2_ along the reaction coordinate and permits partial conversion. Moving from pure CO_2_ to 4% CO_2_ at 1 bar lowers the boundary to the dilute-feed 50% conversion point by a median 93.8 K across 16 reactions with located inputs. R6 and R7 remain provisional and are excluded from that aggregate. Selected-source sensitivities give 93.8–94.3 K; the wider JANAF magnesite enthalpy interval gives 92.4–95.2 K, with other inputs fixed. These conditional ranges are not comprehensive confidence intervals. At 15% CO_2_ and 1 bar, the CaO 90% boundary is 941.6 K. Four Class II reactions release H_2_O(g) and are curved without inert; the Class III reaction consumes H_2_O(g), and has a linear profile only for an undiluted stoichiometric CO_2_/H_2_O charge. The database records 104 thermochemical entries for 32 species. Literature comparisons and numerical verification are reported separately. The screen assumes pure phases and single reactions; the Na_2_CO_3_/NaHCO_3_ route and Li_8_SiO_6_ are excluded because sufficiently complete, traceable inputs were not assembled.

## Abstract 2

For 16 source-located Class I reactions at 1 bar, the 4% CO_2_ finite-charge 50% conversion boundary lies a median 93.8 K below the pure-CO_2_ step boundary. Feed-specific targets distinguish favorable chemistry from attainable conversion.

## Graphical abstract

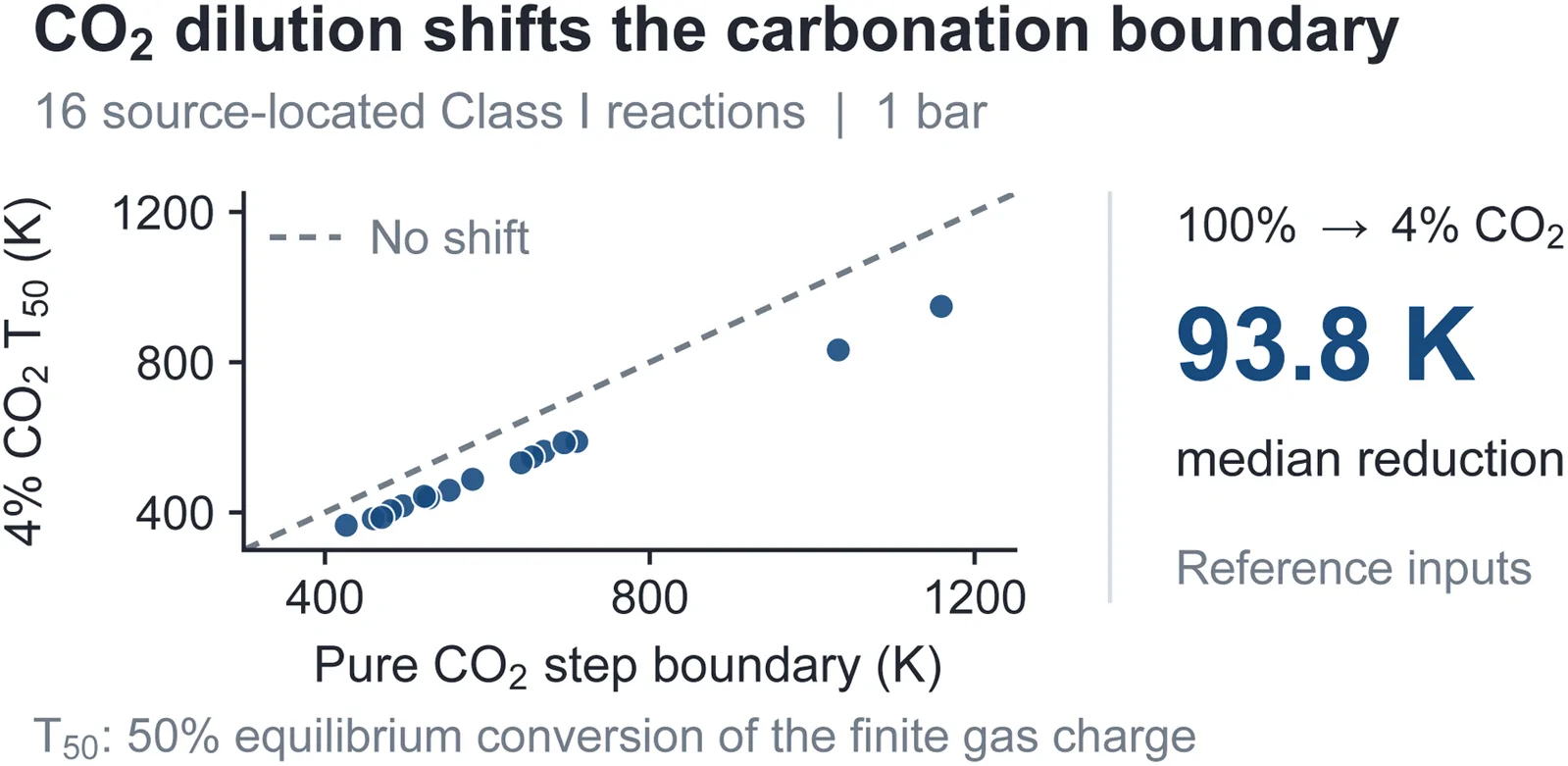

## 1 Introduction

Mineral carbonation, the reaction of CO_2_ with metal oxide, hydroxide or silicate solids to form carbonates, can retain carbon in stable carbonate phases over geological timescales.^1–3^ Field studies in basalt have reported rapid mineralization where reactive surface is accessible,^4,5^ and the *ex situ* route continues to be assessed for peridotite, serpentinized ultramafic rock and alkaline residues.^6,7^ The same oxide and carbonate equilibria underpin calcium looping.^8,9^

Six groups of solids carry most of the interest. Alkaline earth oxides and hydroxides (MgO, CaO, Ca(OH)_2_, Mg(OH)_2_) are the simplest, brucite in particular carbonating readily in dilute streams.^10^ Magnesium silicates and the serpentine group hold the reserves needed for large deployment.^3,11,12^ Calcium and calcium–magnesium silicates include wollastonite^13^ and reactive silicate-bearing slag phases.^14,15^ Aluminosilicates such as anorthite and the garnets are included for compositional coverage rather than for demonstrated reactivity. Alkali ceramics (Li_4_SiO_4_, Li_2_ZrO_3_, Na_2_ZrO_3_, Li_2_SiO_3_) have been widely studied as high-temperature sorbents, with cyclic performance dependent on composition and processing.^16–19^ Dolomite formation closes the set.

Choosing between these materials, fixing an operating window and sizing a carbonator all depend on the equilibrium composition rather than on the sign of the standard reaction Gibbs energy. A negative Δ*G*° states that the reaction is favorable with every species in its standard state, which for a gas means a partial pressure of 1 bar. A flue gas at 15% CO_2_ is far from that reference, and the equilibrium extent there can be small while Δ*G*° remains comfortably negative, because the chemical potential of CO_2_ depends on its mole fraction and on the total pressure. For the dry Class I cases considered here, screening from Δ*G*°(*T*) alone can overstate the accessible temperature window.

Several classes of tool address chemical equilibrium, all of them more general than the calculation used here. Commercial packages such as FactSage^20^ and HSC Chemistry^21^ carry large assessed databases and general Gibbs energy minimizers, but their implementations and full assessed databases are proprietary. Thermo-Calc and the SGTE substance databases occupy the same position. The NASA CEA code^22^ covers gas and condensed phases with a focus on combustion. Cantera,^23^ OpenCalphad^24^ and the CALPHAD framework more generally^25^ handle solution phases and multi-reaction systems that a single reaction coordinate cannot represent, and require an appropriate species set and thermodynamic database. Density functional theory with lattice phonon dynamics provides detailed first-principles property estimates for individual sorbents.^26–29^ Broad screening still requires consistent phase and reference-state conventions. Computational screening across sorbent and solvent classes is now an established route to candidate selection.^30,31^

Ojelade and Zaman^32^ set out the extent-of-reaction form of Gibbs energy minimization for NH_3_ decomposition, deriving *G*(*T*, *P*, *ξ*) and minimizing it numerically. Theirs is an all-gas system, in which every species contributes to the mixing entropy. The solid–gas case behaves differently: pure condensed phases have unit activity and contribute no mixing term, so the shape of *G*(*ξ*), and with it the character of the equilibrium, is set by which species occupy the gas phase along the reaction coordinate and by whether a diluent is present. That difference is the origin of the gap between Δ*G*°-based feasibility statements and equilibrium conversions in specified gas charges.

Gibbs energy minimization is long established, and no claim of a new equilibrium principle is made here. The contribution is threefold. First, the shape of the Gibbs-energy profile is related to gas-phase reactant and product roles, net gas stoichiometry and the initial charge: the reactions sort into three classes according to whether CO_2_ is the only gaseous participant, shares the gas with a coproduct or reacts with a gaseous co-reactant. Second, the thermochemical inputs are curated at parameter level: source, locator, validity range, method flag and fit residual are recorded for 104 entries covering 32 species, with three unlocated inputs explicitly flagged; no heat capacity is estimated by oxide additivity. Third, paired pure- and dilute-charge calculations quantify the shift in screening temperatures, with source sensitivities and 90% conversion boundaries alongside the 50% measure. The set contains 21 reactions with located input sources and two provisional cases (R6 and R7); it is not a comprehensive survey of mineral carbonation. Sections 2 and 3 set out the framework and the underlying thermodynamics, Section 4 the database and its quality control, Section 5 the results and Section 6 what they do and do not support.

## 2 Thermodynamic framework and numerical method

### 2.1 Overview

The calculation proceeds in three stages of input, processing and output, with a fourth stage of verification and benchmarking applied to the outputs. The input stage holds a species database of Shomate coefficients, formation enthalpies, standard entropies, phase identifiers and, for every parameter, its source and validity range; a reaction definition normalized per mole of CO_2_ consumed; and the operating conditions. The processing stage evaluates 
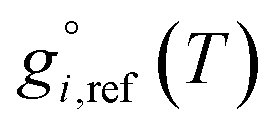
 for every species, constructs *G*(*T*, *P*, *ξ*) as an explicit function of the reaction extent and minimizes it to locate *ξ*_eq_. The output stage returns the equilibrium extent, CO_2_ conversion, equilibrium constant, chemical potentials, turnover temperatures, a validity flag for every reported temperature and the *G*(*ξ*) profile.

Each database entry holds the standard formation enthalpy 
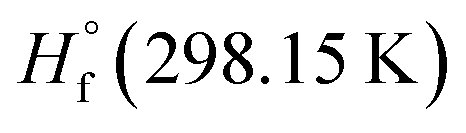
, the standard entropy *S*°(298.15 K), a phase tag and a list of Shomate ranges, each with its temperature interval, the eight coefficients A to H and, where a phase transition falls at a boundary, the enthalpy of that transition. The database covers 32 species, two reactive gases (CO_2_, H_2_O) and 30 condensed species, including liquid carbonates. Every value carries a row in a provenance table (Table S1, SI) giving the parameter, its value, its validity range, a method flag, the citation, the DOI or ISBN, the table or page locator within that source and, for fitted coefficients, the largest residual against the source data. Three unlocated inputs are flagged as provisional, as discussed in Section 4.4.

A calibration routine enforces thermodynamic self-consistency before any property is evaluated. Using the reference-range polynomial at 298.15 K, the F and G coefficients are set so that the enthalpy increment vanishes at the reference temperature and *S*(298.15) = *S*°(298.15). Where a species has several ranges, continuity of *H*(*T*) and *S*(*T*) is enforced at each ordinary boundary; a first-order transition instead contributes its latent enthalpy and the corresponding entropy Δ*H*_tr_/*T*_tr_, preserving continuity of Gibbs energy. These consistency conditions do not remove uncertainty in the measured or assessed inputs.

### 2.2 Reaction definition

Reactions are stored as (species, stoichiometric coefficient) pairs, negative for reactants and positive for products, each normalized to one mole of CO_2_ consumed so that reaction properties are comparable across sorbents. All reactions balance on every element. Condensed reactants are charged in stoichiometric quantities; condensed products are initially absent. The principal dry-feed cases contain one mole of CO_2_ and an inert loading *I* = (1 − *y*_0_)/*y*_0_, where *y*_0_ is the initial dry CO_2_ fraction. R24 additionally receives two moles of water per mole of CO_2_. Its actual gas composition is therefore (1, 2, *I*)/(3 + *I*), rather than the nominal dry composition. Separate humidity comparisons specify the actual initial fractions of all three gases. All target conversions refer to a finite charge at fixed total pressure, not to a continuously replenished gas stream.

### 2.3 Locating the equilibrium extent

Equilibrium is located by bounded scalar minimization on 0 ≤ *ξ* ≤ *ξ*_max_ using scipy.optimize.minimize_scalar, method bounded, with xatol = 10^−10^. Each objective evaluation computes the moles of all species at the current *ξ*, forms the gas-phase mole fractions and sums the chemical potential contributions. Turnover temperatures, at which carbonation ceases to be favorable at a given CO_2_ partial pressure, come from scipy.optimize.brentq root finding on Δ*G*°(*T*) − *RT* ln(*P*_CO_2__/*P*°) = 0 over 300–2000 K. That is the general condition of Section 3.2 evaluated with every gas species other than CO_2_ held at 1 bar, so for the five reactions that produce or consume H_2_O(g) it returns a *p*_H_2_O_ = 1 bar value, reported alongside other explicitly stated water conventions in Table 2. Finite-charge *T*_50_ and *T*_90_ instead solve eqn (11) at the specified conversion, with an absolute temperature tolerance of 10^−8^ K. The interval is scanned together with phase boundaries to bracket roots; endpoint behavior is checked separately. The Gibbs-energy derivative used here is the negative of the reaction affinity. Calculations used Python 3.10.12, NumPy 2.2.6 and SciPy 1.15.3,^33,34^ with *R* = 8.314 J mol^−1^ K^−1^. The SI and accompanying CSV tables provide the thermochemical inputs, equations and numerical outputs for independent implementation and verification.

The main state grid contains 1495 evaluations (23 reactions, 13 temperatures and five gas conditions). The imposed-partial-pressure grid has 92 entries, of which 89 have a root within 300–2000 K. Additional calculations resolve 50% and 90% targets over five feed compositions and two total pressures, together with paired source sensitivities and matched humid feeds. These roots are calculated directly rather than interpolated from the 13-temperature grid.

### 2.4 Numerical verification and gas nonideality

For a single reaction among pure condensed phases and one ideal-gas phase, minimizing *G*(*ξ*) along the reaction coordinate and minimizing *G* over the composition space of the specified species set subject to the element balances are mathematically equivalent when the element matrix has nullity one, as it does for each reaction here, so any disagreement is a numerical or coding difference rather than a modeling choice. A separate element-potential (RAND) solver was formulated from the published algorithm of White, Johnson and Dantzig^35^ in the form given by Smith and Missen.^36^ The second formulation treats every species mole number as an unknown, imposes the element balances through Lagrange multipliers and determines the condensed phases present by enumeration rather than assumption. Its stationarity condition is 
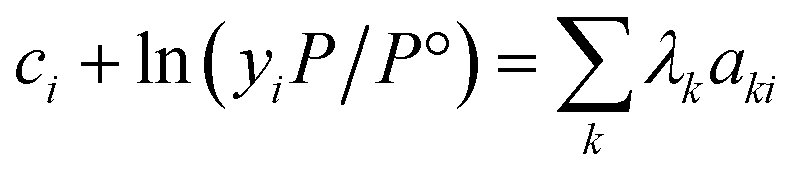
 for gas species and 
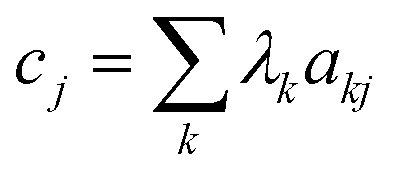
 for condensed phases present, where 
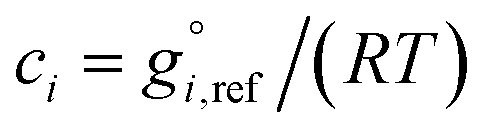
, *a*_*ki*_ is the number of atoms of element *k* in species *i* and *λ*_*k*_ is the dimensionless element potential (SI Section S4). The two formulations share the thermochemical database but no part of the reaction-coordinate machinery, so the comparison tests implementation consistency and the completeness of the one-dimensional parameterization, and says nothing about whether the underlying data are correct. It does not establish global equilibrium when competing reactions or additional phases are admitted.

Representative nonideal comparisons replace *y*_*i*_*P* by *ϕ*_*i*_*y*_*i*_*P* using the Peng–Robinson (PR) equation of state (EOS),^37^ with classical mixing rules and zero binary interaction parameters. Critical constants and acentric factors are documented with their sources in SI Section S7.^38^ The fugacity coefficients are evaluated at the depleted gas composition at the target extent and at the total pressure. The dry-gas comparison covers five sorbents, three pressures and three initial compositions. Four humid cases are supplementary sensitivity checks. A zero binary interaction parameter is a poor description of the CO_2_/H_2_O pair in particular. Those four cases therefore illustrate sensitivity to this gas-phase correction and are not a calibrated humid-mixture result. They are not a replacement for a water condensation or electrolyte calculation.

## 3 Theory

### 3.1 Gibbs energy of a mixed-phase system

The starting point is the total Gibbs energy of a multicomponent system at fixed temperature and pressure,^39,40^1
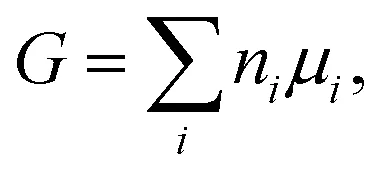
where *n*_*i*_ and *µ*_*i*_ are the moles and chemical potential of species *i*, and at equilibrium *G* is a minimum with respect to all compositional changes allowed by mass conservation. Here the allowed changes follow one balanced reaction among the specified species; this is a restricted equilibrium. For a pure condensed phase the activity is unity, so2
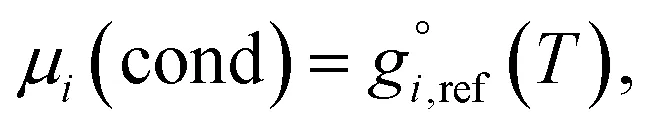
while for an ideal gas species^32^3
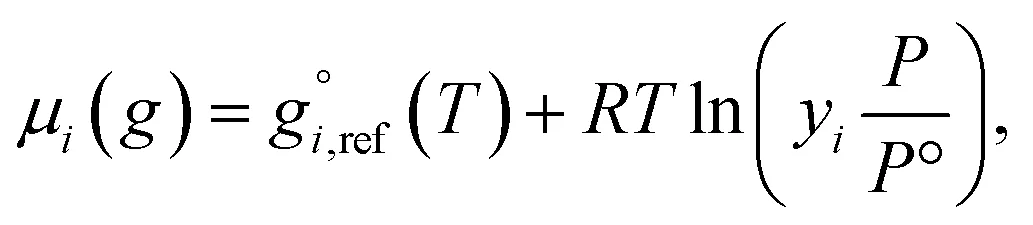
with *y*_*i*_ the gas-phase mole fraction, *P* the total pressure and *P*° = 1 bar. The reference chemical potential 
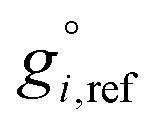
 is defined below in eqn (17). The extent of reaction *ξ* fixes the composition through4
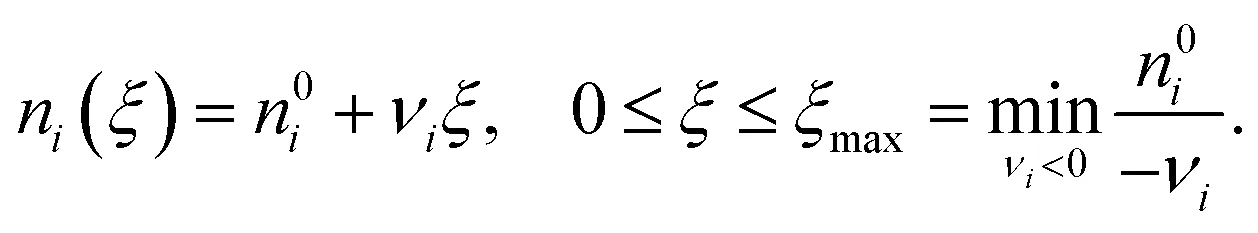
with *ν*_*i*_ negative for reactants and positive for products. Stoichiometric initial conditions, *n*^0^_*i*_ = |*ν*_*i*_| for reactants and *n*^0^_*i*_ = 0 for products, make *ξ* run from 0 at no reaction to 1 at complete conversion. Every reaction consumes one mole of CO_2_ per unit extent, so *ξ* equals the fraction of the initial CO_2_ converted. Insufficient water lowers *ξ*_max_ for R24. Substituting the chemical potentials and mole numbers into eqn (1) gives5
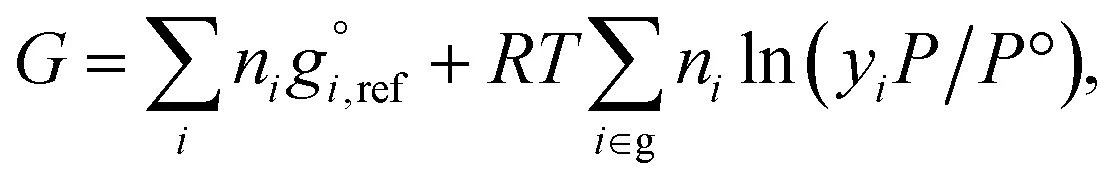


Flue gas is represented by an inert carrier (N_2_) at a loading *I* = (1 − *y*_CO_2_,feed_)/*y*_CO_2_,feed_ per mole of CO_2_, and the equilibrium extent follows from6
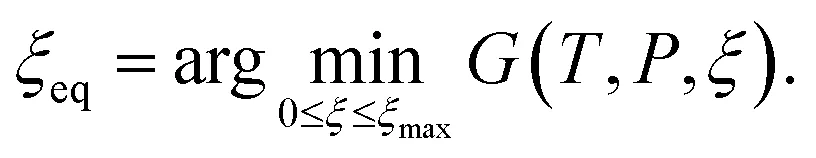


The gas sum includes the inert: its standard contribution is constant, but its mixing contribution must be retained. Condensed-phase pressure corrections are neglected; Section 5.7 quantifies the omission. The charge is closed to reactive matter while exchanging heat and mechanical work with its surroundings, so gas volume changes at fixed pressure. It is not a continuously replenished gas reservoir. Initial gas fractions are proportional to (1, *w*_0_, *I*) for water loading *w*_0_.

### 3.2 The equilibrium condition and the gas-stoichiometry classes

Eqn (5) contains two contributions. The sum of 
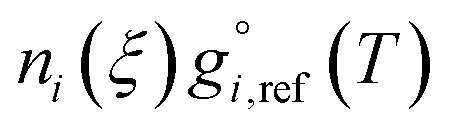
 is the standard part *G*°(*ξ*), linear in *ξ* by construction; the *RT* ln(*y*_*i*_*P*/*P*°) terms of the gas species supply the mixing and pressure correction, and the solids contribute nothing to it. Differentiating with respect to *ξ* therefore gives the interior equilibrium condition in its general form,7
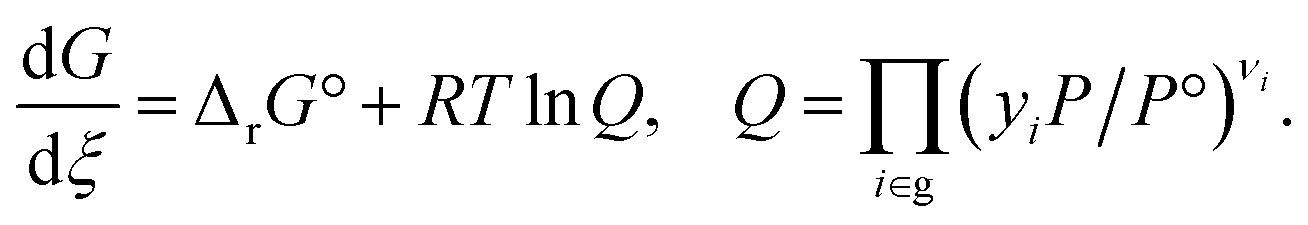
in which only the gas-phase species appear. In an all-gas system such as NH_3_ decomposition^32^ every species enters *Q*, but the existence of an interior minimum depends on the charge and reaction conditions. In solid–gas carbonation which species enter *Q* is a property of the reaction, and it sorts the 23 reactions of this work into three classes. An interior minimum satisfies d*G*/d*ξ* = 0; the right derivative is nonnegative at a minimum at zero, and the left derivative is nonpositive at a minimum at *ξ*_max_. The curvature is8
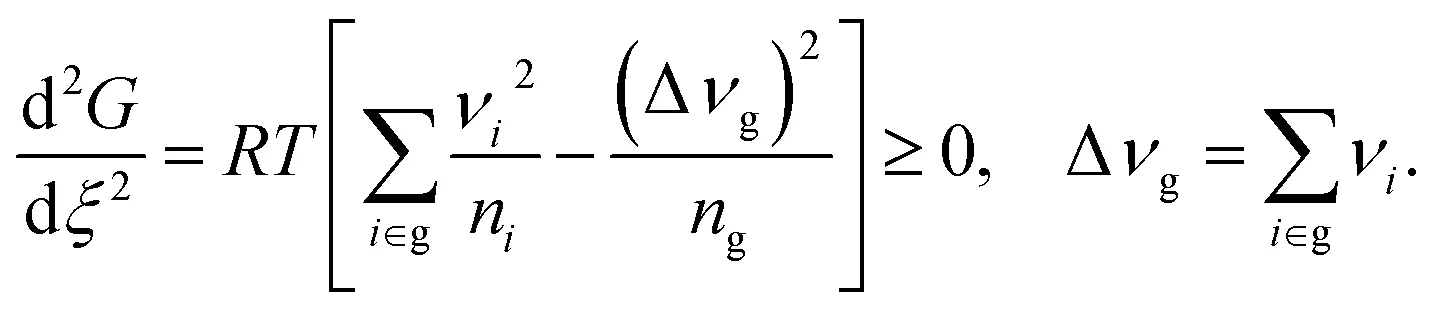
Cauchy–Schwarz establishes the inequality. Equality requires *ν*_*i*_/*n*_*i*_ to be identical for all present gas species. Profile shape therefore depends on both gas stoichiometry and the initial charge.

Class I, CO_2_ the only gaseous species: 18 reactions. Here *Q*^−1^ = *y*_CO_2__*P*/*P*°. With no diluent *y*_CO_2__ = 1 while gas remains, the correction is constant, *G* is strictly linear in *ξ*, the minimum lies at an end point and equilibrium is binary except at the flat step boundary, where no unique conversion is selected. With a diluent, carbonation removes CO_2_ while the inert carrier remains, so *y*_CO_2__ falls as *ξ* advances and the reaction-quotient correction −*RT* ln(*y*_CO_2__*P*/*P*°) becomes progressively more positive. The profile acquires curvature, and where the standard driving force is modest its derivative crosses zero inside the physical domain: equilibrium is partial conversion at the composition for which the reaction-quotient penalty balances the standard driving force. An undiluted calculation predicts a step change between full conversion and none at the turnover temperature, whereas the curvature restored by a diluent permits partial conversion over a temperature interval.

Class II, a gaseous coproduct: 4 reactions. Carbonation of Ca(OH)_2_, Mg(OH)_2_, chrysotile and talc releases H_2_O(g), so the quotient carries a water term, 
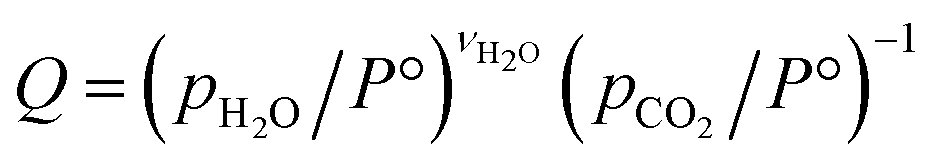
, and the gas is a CO_2_–H_2_O mixture as soon as the reaction starts even from an undiluted CO_2_ charge. These profiles are curved with no inert present at all. The normalized chord departures in Fig. 1 isolate this mixing curvature from the standard reaction energy.

**Fig. 1:**
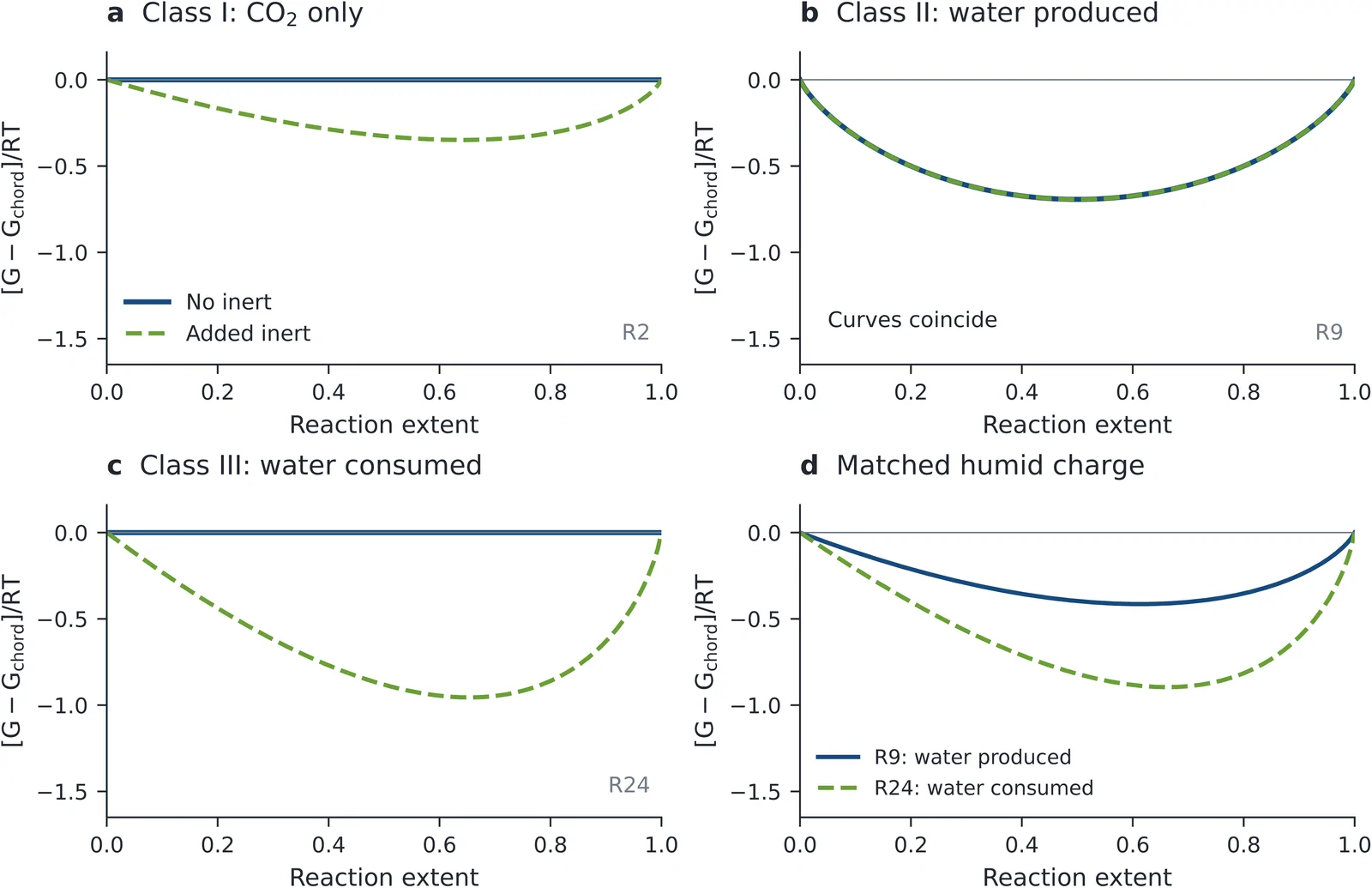
Gas stoichiometry and initial charge jointly determine profile curvature. The ordinate subtracts the straight chord between the endpoint Gibbs energies and divides by *RT*; it isolates mixing curvature and does not locate the equilibrium minimum. (a) R2: one gaseous reactant, no gaseous product, Δ*ν*_g_ = −1. (b) R9: one gaseous reactant and one gaseous product, Δ*ν*_g_ = 0. (c) R24: two gaseous reactant species, no gaseous product, Δ*ν*_g_ = −3. Panels (a)–(c) compare no inert with *I* = 5.667 mol per mole of initial CO_2_; R24 contains two initial moles of water in both cases. (d) R9 and R24 under the same actual initial composition: 15% CO_2_, 30% H_2_O and 55% N_2_. Both are curved, although water retains its different stoichiometric role.

Adding inert at fixed extent and unchanged reactive mole numbers multiplies every reactive gas mole fraction by the common factor *f* = *n*_gas,reactive_/(*n*_gas,reactive_ + *I*), so that, with 
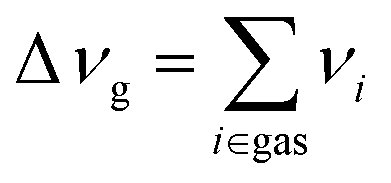
 the net change in the number of gas moles,

9 ln *Q*(*ξ*; *I*) = ln *Q*(*ξ*; 0) + Δ*ν*_g_ ln *f*.

When Δ*ν*_g_ = 0 the reactive gas count does not change along the reaction coordinate, *f* is independent of *ξ*, and ln *Q* is unshifted at every extent. The derivative d*G*/d*ξ*, and hence the shape of *G*(*ξ*) up to an additive constant, *ξ*_eq_(*T*) and the 50% conversion temperature, are therefore independent of the inert loading and of the total pressure for unchanged reactive inventories under ideal-gas assumptions. The result is exact rather than a small-effect argument; it does not remove humidity dependence. Two reactions satisfy it, portlandite and brucite, each releasing one mole of H_2_O(g) per mole of CO_2_ consumed, with equilibrium ratio *p*_H_2_O_/*p*_CO_2__ = exp[−Δ_r_*G*°/(*RT*)]. Fugacity corrections generally break the exact pressure cancellation.

Class III, a gaseous co-reactant: 1 reaction. The hydrous anorthite route consumes two moles of H_2_O(g) per mole of CO_2_, so water enters *Q* with a negative exponent and Δ*ν*_g_ = −3. For an undiluted stoichiometric CO_2_/H_2_O charge, the 1 : 2 ratio is maintained along the reaction coordinate, with *y*_CO_2__ = 1/3 and *y*_H_2_O_ = 2/3; *Q* is therefore constant and *G*(*ξ*) is linear. With an inert gas present, the reactive gases retain their 1 : 2 ratio but their combined fraction changes with *ξ*. Because Δ*ν*_g_ = −3, *Q* then changes with extent and *G*(*ξ*) is not generally linear. Excess water also restores curvature without changing the gaseous co-reactant class. The reaction is treated as a humid-gas case throughout, with all three mole fractions stated for every condition. Thus water's reactant/product role defines the class independently of the feed; that role, Δ*ν*_g_ and the initial composition must be reported together.

### 3.3 Target conversion and imposed-gas turnover

The turnover temperature. Evaluating *Q* with every gas species other than CO_2_ held at 1 bar reduces eqn (7) to10
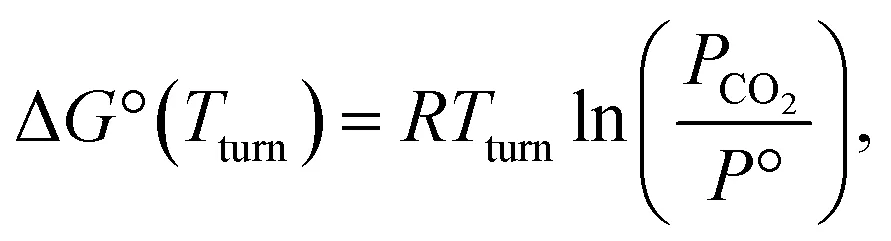
since *ν*_H_2_O_ ln 1 = 0 whatever *ν*_H_2_O_ may be. This imposed-gas condition is therefore exact for Class I under the ideal-gas assumption, and for Classes II and III it is one member of a two-variable family: a turnover temperature is not defined there by *P*_CO_2__ alone, and the water partial pressure must be specified alongside it. The choice *p*_H_2_O_ = 1 bar gives one reference member of that family; Table 2 reports other stated water conditions separately.

For a specified finite charge, the temperature at target conversion *c* instead satisfies

11 *F*(*T*_*c*_, *c*) = Δ_r_*G*°(*T*_*c*_) + *RT*_*c*_ ln *Q*(*c*) = 0.

*T*_50_ and *T*_90_ correspond to *c* = 0.5 and 0.9. For linear profiles, the root is the step boundary described above; elsewhere the target is evaluated on the declining-conversion temperature branch. For a dry Class I charge,12
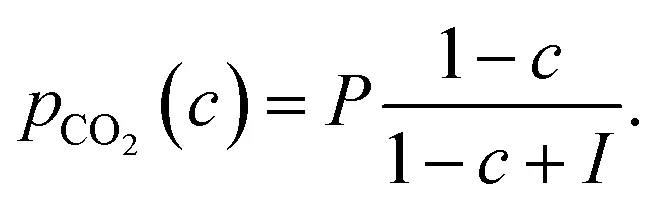


Thus a 4% dry charge at 1 bar gives *p*_CO_2__ = 1/49 bar at 50% conversion. If reaction enthalpy and entropy are treated as temperature independent, *T*_*c*_ = Δ_r_*H*°/[Δ_r_*S*° + *R* ln(*p*_CO_2__/*P*°)]. With *L* = ln[*P*°/*p*_CO_2__(*c*)] at a total pressure of 1 bar, the pure-to-dilute shift is *T*_pure_*RL*/[|Δ_r_*S*°| + *RL*]. This closed form omits heat-capacity and phase-transition effects; the reported numerical roots include them. It also shows why a set with similar reaction entropies has shifts close to a fixed fraction of the pure-feed temperature. An imposed-gas turnover need not equal this finite-charge target temperature, and neither specifies a reaction rate or residence time.

Away from phase boundaries, implicit differentiation gives the local sensitivity to input *θ*:13
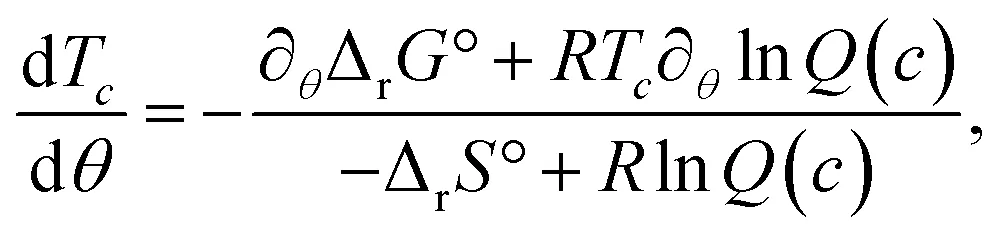
for temperature-independent ideal-gas feed and pressure specifications. Finite source changes and intervals crossing phase transitions are evaluated by resolving eqn (11), rather than extrapolating this derivative.

### 3.4 Temperature-dependent properties

Temperature-dependent properties come from Shomate polynomials,^41^ with *t* = *T* (K)/1000:14
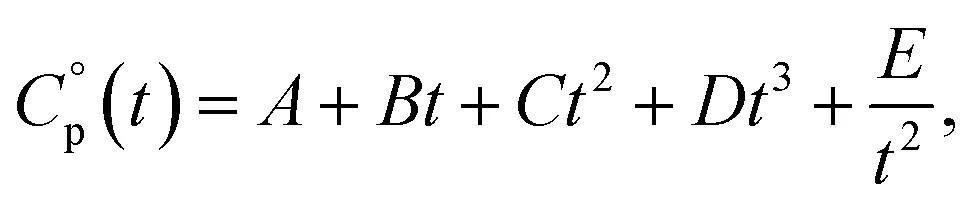
15
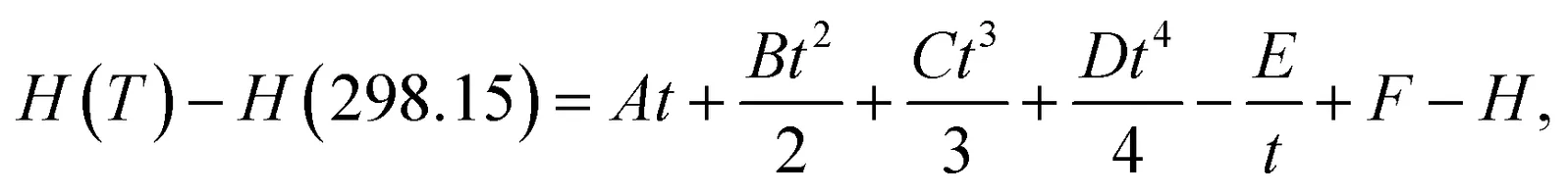
16
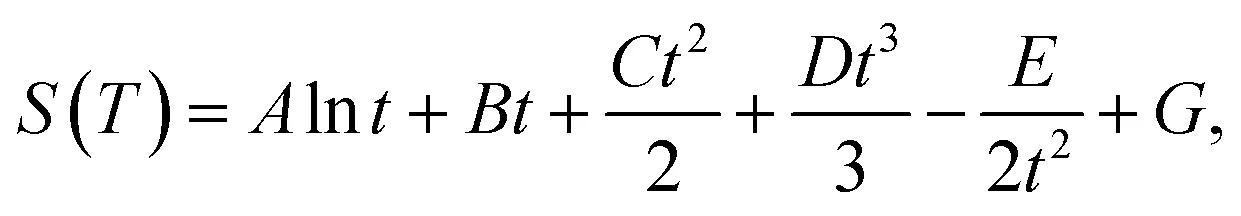
with 
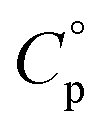
 and *S* in J mol^−1^ K^−1^ and the enthalpy increment in kJ mol^−1^. The reference chemical potential follows as17
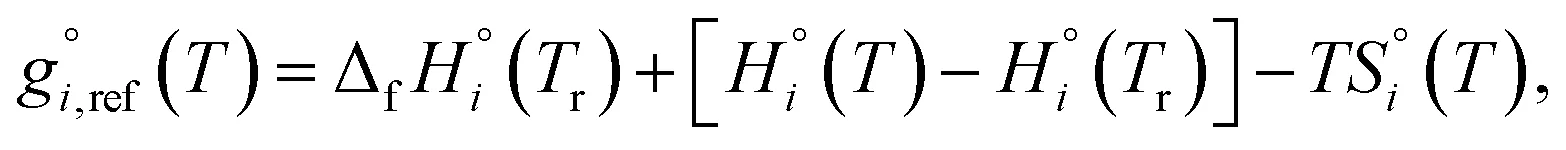
where *T*_r_ = 298.15 K and 
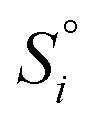
 is the absolute entropy. This is not the Gibbs energy of formation from the elements at *T*. Elemental reference terms cancel in balanced reaction sums and remain constant during element-conserving minimization, so 
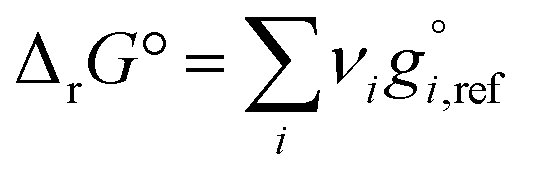
 is the standard reaction Gibbs energy. The six first-order transitions carried in the database contribute both Δ*H*_tr_ and Δ*H*_tr_/*T*_tr_, preserving continuity of Gibbs energy. The unresolved transition is listed separately in SI Table S1(d).

## 4 Thermochemical database, provenance and quality control

### 4.1 Source selection and heat capacities

Technological relevance defines the candidate families, while sufficiently complete, traceable thermochemistry determines admission to the primary screen. That database covers 23 reactions and 32 species through 104 provenance entries. The method census is 25 parameters taken directly from NIST-JANAF tables,^41,42^ 53 from critically assessed compilations,^43–45^ 20 fitted to published heat-capacity tables or equations, 3 from primary calorimetry and 3 unlocated inputs. R6 and R7, which use those inputs, are provisional and excluded from the primary aggregate. Each located anchor is attributed to the compilation that prints it; the mixed source choices are not claimed to form a single internally assessed dataset.

To avoid introducing estimated heat-capacity trends into the screening analysis, only measured, critically assessed or directly fitted source data were admitted to the final thermochemical database; no heat capacity is obtained from Neumann–Kopp oxide additivity. For the lithium and sodium heat capacities this means Li_2_CO_3_, Na_2_CO_3_ and Li_2_SiO_3_ from NIST-JANAF,^41^ Li_2_ZrO_3_ and Li_4_SiO_4_ from Kleykamp's drop calorimetry,^46,47^ and Na_2_ZrO_3_ from the calorimetry of Beyer, Bennington and Brown.^48^ Kleykamp's enthalpy fits are of Maier–Kelley form, so conversion to Shomate coefficients is exact term by term, and the Na_2_ZrO_3_ fit reproduces the source's 25 smoothed heat capacities to 0.211%. The silicate and aluminosilicate phases were fitted by least squares to the printed tables of Robie and Hemingway,^43^ the parameter subset being fixed by a rule set in advance rather than tuned per species; the largest residual over all fitted species is 0.65%, for talc, against a limit of 1%. The brucite fit reproduces the printed table to 0.0014%, and the chrysotile fit reproduces the table on p. 394 to 0.111%. These are fit residuals, not experimental uncertainties. Formation anchors are attributed separately in Table S1: the Li_2_CO_3_, Li_2_SiO_3_ and Li_2_ZrO_3_ enthalpies and the first two entropies are NBS values,^44^ and the Na_2_ZrO_3_ pair is Beyer's calorimetry.

### 4.2 Phase transitions

Enthalpy cannot be made continuous across a first-order phase transition without discarding its latent heat, so a calibration rule that enforces continuity of *H*(*T*) at every Shomate range boundary silently deletes it. Li_2_CO_3_ melts at 993 K with an enthalpy of fusion of 44.687 kJ mol^−1^ and Na_2_CO_3_ at 1123 K with 29.660 kJ mol^−1^, and both have a lower solid–solid transition (Li_2_CO_3_ at 683 K, 2.762 kJ mol^−1^; Na_2_CO_3_ at 723 K, 0.678 kJ mol^−1^).^41^ The calibration routine therefore carries an explicit transition enthalpy and its corresponding entropy per range, reducing to plain continuity when that enthalpy is zero. It also includes the ZrO_2_ monoclinic-to-tetragonal transition at 1478 K (5.941 kJ mol^−1^) and the quartz α–β transition at 847 K (0.729 kJ mol^−1^). All six obey Δ*H*_tr_ = *T*_tr_Δ*S*_tr_. Every turnover temperature is also reported on a solid-only carbonate basis to isolate melting. This diagnostic continuation does not reproduce other solid structures or reference data that may differ in literature calculations.

### 4.3 The magnesite reference state

Selected determinations lie between −1111.68 kJ mol^−1^ from reversed phase equilibrium and −1114.51 kJ mol^−1^ for the Chernosky–Berman estimate tabulated by Koziol and Newton,^49^ with Robie and Hemingway at −1113.30;^43^ the NBS value, −1095.80 kJ mol^−1^,^44^ sits 15.9 kJ mol^−1^ outside that band. The database uses −1111.69 kJ mol^−1^, retaining the measured entropy of Hemingway *et al.*^50^ at 65.09 J mol^−1^ K^−1^. This is a documented combination of sources. The printed JANAF assessment is −1111.7 ± 8 kJ mol^−1^ and was last reviewed in December 1966.^41^ The selected-source range is not a comprehensive confidence interval.

Koziol and Newton^49^ bracket MgCO_3_ → MgO + CO_2_ at 988.15 K and 590 ± 30 bar; with the adopted anchor the ideal-gas turnover lies at 987.2 K. The pressure interval spans approximately 983 to 991 K on their reversal curve, so this is agreement within a bracket, not a 1 K accuracy statement. The experiment shares thermochemical information with, and informed assessment of, the anchor; it is also far above the model's pressure range. At their tabulated 988 K, 600 bar state, the reported CO_2_ fugacity is 682 bar. Its contribution to the reaction Gibbs energy is −1.05 kJ mol^−1^, whereas the source's condensed-phase molar volumes give +1.01 kJ mol^−1^ over 1 to 600 bar. Their near cancellation explains why omitting both terms can give close agreement. This diagnostic is not a new reconstruction of the 590 bar reversal.

Separately, Bénézeth *et al.*^51^ report Δ_f_*H*°(298.15) = −1111.75 ± 2 and Δ_f_*G*°(298.15) = −1026.48 ± 2 kJ mol^−1^ (3*σ*) from aqueous solubility measurements. Their enthalpy agrees with the adopted anchor to 0.06 kJ mol^−1^. The present formation Gibbs energy is −1027.90 kJ mol^−1^, differing by 1.42 kJ mol^−1^, or approximately 2.1*σ*. The difference is predominantly entropic: their entropy is 60.00 against the adopted 65.09 J mol^−1^ K^−1^. A separate comparison using their measured magnesite solubility product with CODATA key values for Mg^2+^(aq) and CO_3_^2−^(aq)^45^ gives −1027.79 kJ mol^−1^, a deviation of 0.10 kJ mol^−1^. These comparisons support the adopted range but do not validate low-pressure dilute-feed carbonation or establish that differing first-principles values are erroneous.

### 4.4 Coverage and validity

The Na_2_CO_3_ + CO_2_ + H_2_O(g) → 2NaHCO_3_ route is excluded because a sufficiently traceable nahcolite heat-capacity function covering the intended range was not assembled from the sources examined.^42,43^ Li_8_SiO_6_ is also excluded because the required formation enthalpy, absolute entropy and heat-capacity dataset was not assembled. Within the admitted set, the Li_4_SiO_4_ formation enthalpy and entropy and the Li_2_ZrO_3_ entropy are printed by none of NIST-JANAF, Robie and Hemingway, the NBS tables or CODATA. These values are treated as provisional assumptions, not as sourced measurements. R6 and R7 are excluded from the primary aggregate and are retained for sensitivity analysis only. Alternative reaction-level anchors for R6 and R7, taken from published reaction-level studies of the same two solids, are evaluated in SI Section S7.3.^27,52^ These are limitations of the present curated screen, not claims that the compounds lack an experimental literature. Further candidates and the source search are recorded in SI Section S6.

Every reported temperature carries the window within which all participating species have heat-capacity data. Of the 92 turnover values, 73 lie inside that window and 16 outside and are flagged in every table and file, the remaining 3 having no root below 2000 K. Six are extrapolations above the data, the two largest being Ca(OH)_2_ at 0.1 and 0.15 bar, 633 and 776 K above the portlandite range, reported as diagnostic extrapolations and not as operating recommendations. Ten are formally below the data because the NIST-JANAF H_2_O(g) range begins at 500 K; the extrapolated polynomial agrees with the tabulated heat capacities to 0.0041% at 300 K and 0.0033% at 400 K. Those checks do not extend the source interval.

### 4.5 Propagation of source choices

Each alternative magnesite anchor is applied consistently to every affected reaction and to both members of each pure/dilute comparison. The resulting shift is therefore a paired quantity, not a difference between unrelated uncertainty bounds. The selected anchors are also crossed with Kleykamp's alternative Li_2_SiO_3_ heat capacity. A broader calculation uses the published JANAF ±8 kJ mol^−1^ enthalpy interval, holding other inputs fixed. For R7, separate calculations retain only solid carbonate or impose the paired reaction enthalpy and Gibbs energy from Duan's 300 K first-principles result while retaining the present heat-capacity functions.^27^ These finite comparisons resolve the target equation across phase transitions. They are conditional source and phase sensitivities; incomplete covariance information prevents their interpretation as comprehensive confidence intervals.

## 5 Results

### 5.1 The reaction set

Table 1 lists the 23 reactions, their gas-stoichiometry class, their standard properties at 298.15 K and the Class I turnover temperatures at four CO_2_ partial pressures, all normalised per mole of CO_2_ consumed. Eighteen belong to Class I, four to Class II and one to Class III in the sense of Section 3.2, with the five water-dependent reactions reported separately in Table 2. R6 and R7 are provisional cases. The set spans six families: alkaline earth oxides and hydroxides (4 reactions), magnesium silicates and the serpentine group (4), calcium and calcium–magnesium silicates including slag phases (6), aluminosilicates (4), alkali ceramic and carbonate sorbents (4) and carbonate to double carbonate (1). Larnite is included as β-Ca_2_SiO_4_, a silicate phase found in some slags;^14,15^ no process or feedstock claim attaches to it here. Below approximately 970 K, this polymorph is metastable with respect to γ-Ca_2_SiO_4_. A more stable reactant would lower the R12 turnover; the shift is not quantified because a traceable heat-capacity function for that polymorph was not admitted (SI Section S6.2).

**Table 1:** The 23 reactions, written per mole of CO_2_ consumed, with the gas-stoichiometry class of Section 3.2, standard properties at 298.15 K and reference turnover temperatures at four imposed CO_2_ partial pressures. Every tabulated value is a restricted, single-reaction equilibrium in the admitted species set of that row; competing reactions, solid solutions and unlisted phases are outside its composition space. The process-relevant finite-charge *T*_50_ and *T*_90_ boundaries are given in Fig. 3 and SI Table S7.1. Classes: I CO_2_ the only gaseous species, II gaseous coproduct, III gaseous co-reactant. Families: F1 alkaline earth oxides and hydroxides, F2 magnesium silicates and the serpentine group, F3 calcium and calcium–magnesium silicates including slag phases, F4 aluminosilicates, F5 alkali ceramic and carbonate sorbents, F6 carbonate to double carbonate

ID	Reaction	Family	Class	Δ*H*° kJ mol^−1^	Δ*S*° J mol^−1^ K^−1^	Δ*G*° kJ mol^−1^	*u*(*T*_turn_)^b^ K	*T*_turn_/K			
								0.1 bar	0.15 bar	1.0 bar	20 bar
R1	MgO + CO_2_ → MgCO_3_	F1	I	−116.6	−175.6	−64.2	±3	601.7	612.5	669.5	786.4
R2	CaO + CO_2_ → CaCO_3_	F1	I	−178.5	−160.3	−130.7	±11	1024.1	1045.4	1159.0	1405.0^a^
R3	Ca(OH)_2_ + CO_2_ → CaCO_3_ + H_2_O(g)	F1	II	−69.1	−16.6	−64.2	—	Water-dependent; see Table 2			
R9	Mg(OH)_2_ + CO_2_ → MgCO_3_ + H_2_O(g)	F1	II	−35.5	−23.1	−28.6	—	Water-dependent; see Table 2			
R5	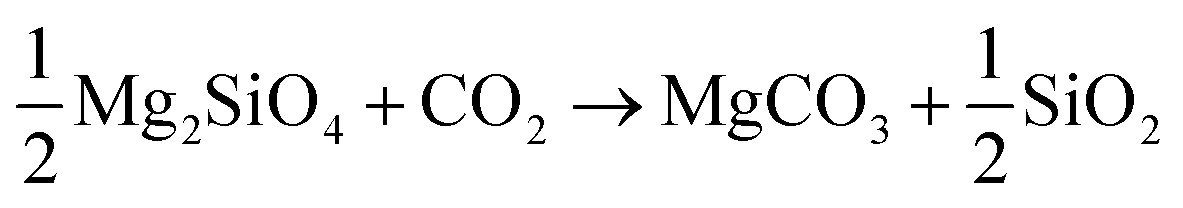	F2	I	−86.5	−175.0	−34.3	±4^c^	446.2	454.2	495.7	580.1
R10	MgSiO_3_ + CO_2_ → MgCO_3_ + SiO_2_	F2	I	−83.3	−173.5	−31.5	±6^c^	432.5	440.3	480.6	562.6
R11	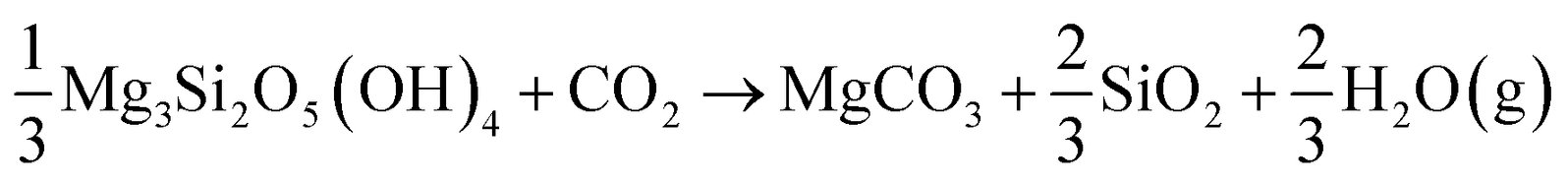	F2	II	−33.2	−68.9	−12.6	—	Water-dependent; see Table 2			
R18	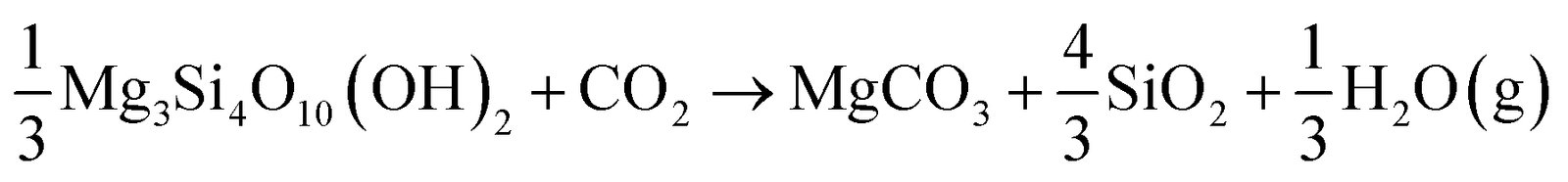	F2	II	−46.4	−117.4	−11.4	—	Water-dependent; see Table 2			
R4	CaSiO_3_ + CO_2_ → CaCO_3_ + SiO_2_	F3	I	−89.1	−162.5	−40.7	±14	492.9	502.5	552.9	658.5
R12	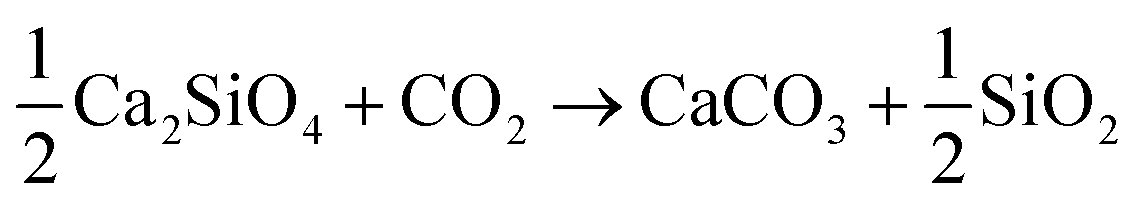	F3	I	−115.4	−165.1	−66.1	±9^c^	632.8	645.1	710.0	846.2
R13	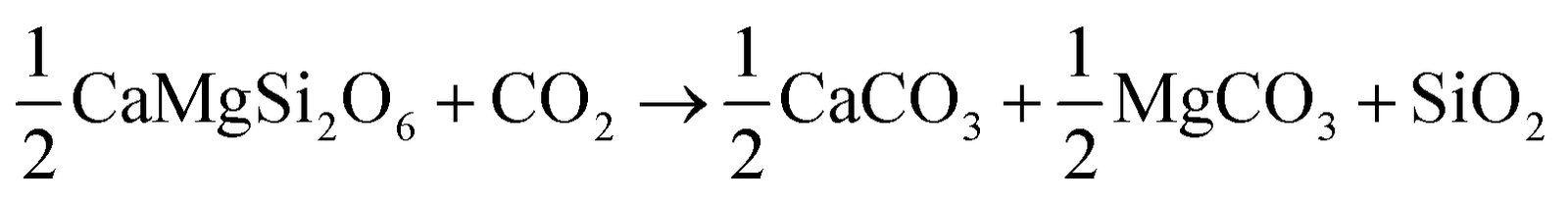	F3	I	−75.7	−165.3	−26.4	±8^c^	411.1	418.9	459.4	543.1
R21	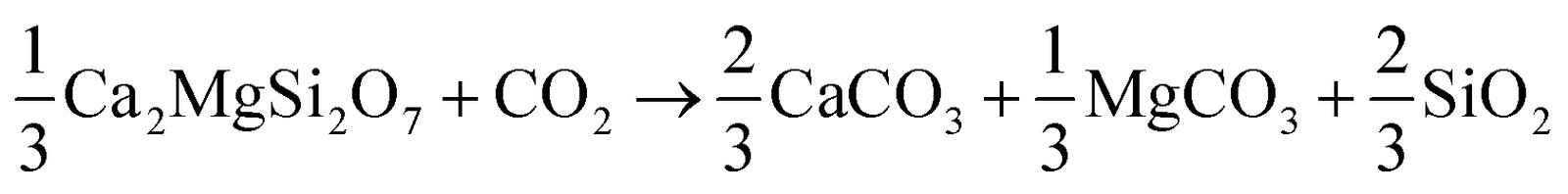	F3	I	−100.5	−174.1	−48.6	±7^c^	522.4	531.9	581.8	684.3
R22	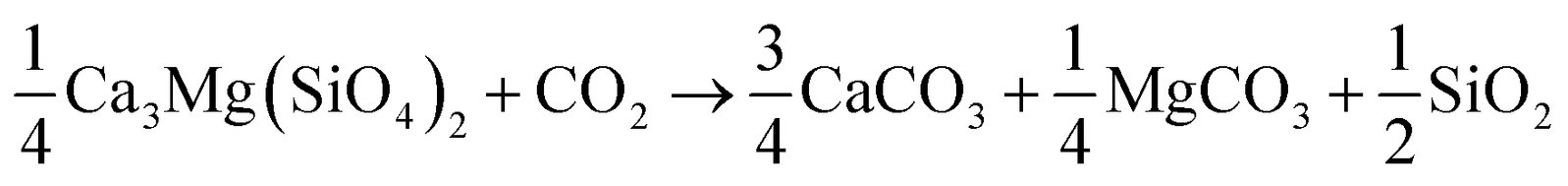	F3	I	−110.9	−171.3	−59.8	±7^c^	586.7	597.6	655.1	774.2
R23	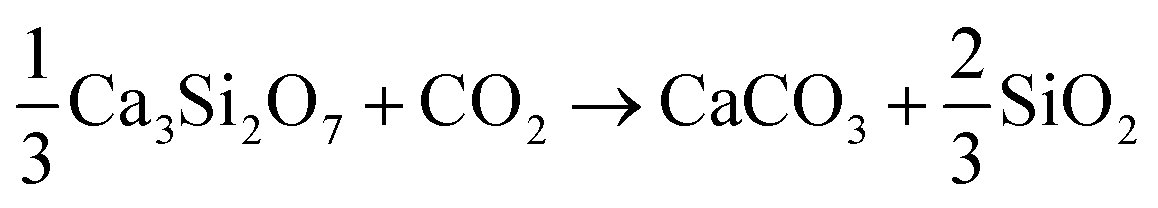	F3	I	−104.2	−164.6	−55.1	±9^c^	571.7	582.8	641.4	764.4
R14	CaAl_2_Si_2_O_8_ + CO_2_ → CaCO_3_ + Al_2_SiO_5_ + SiO_2_	F4	I	−83.9	−197.1	−25.1	±8^c^	388.1	394.3	426.1	489.0
R19	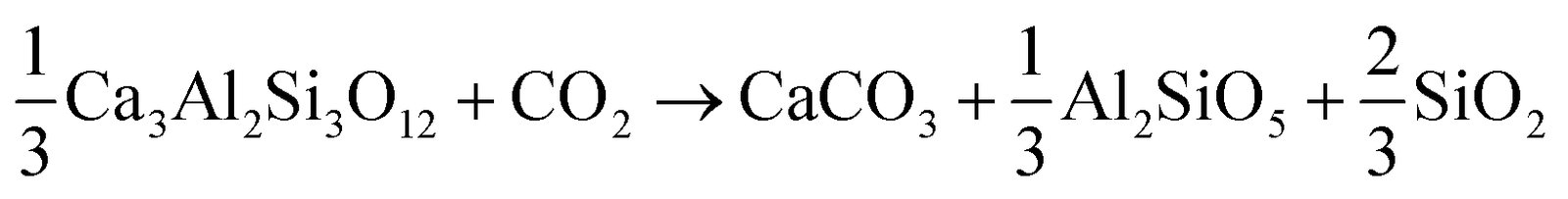	F4	I	−71.8	−153.5	−26.0	±10^c^	416.7	425.2	470.0	565.3
R20	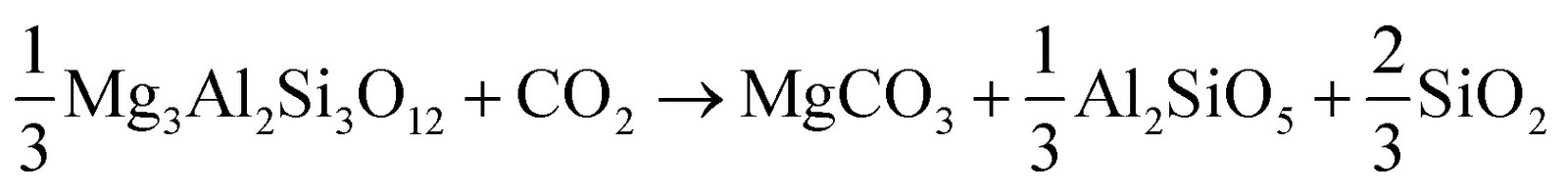	F4	I	−94.9	−182.2	−40.6	±5^c^	472.0	480.2	522.4	607.3
R24	CaAl_2_Si_2_O_8_ + CO_2_ + 2H_2_O(g) → CaCO_3_ + Al_2_Si_2_O_5_(OH)_4_	F4	III	−214.7	−498.6	−66.1	—	Water-dependent; see Table 2			
R6^p^	Li_4_SiO_4_ + CO_2_ → Li_2_CO_3_ + Li_2_SiO_3_	F5	I	−144.3	−165.3	−95.0	—	794.8	810.8	895.9	1121.3
R7^p^	Li_2_ZrO_3_ + CO_2_ → Li_2_CO_3_ + ZrO_2_	F5	I	−162.8	−165.0	−113.6	—	916.8	936.9	1076.5	1675.4^a^
R8	Na_2_ZrO_3_ + CO_2_ → Na_2_CO_3_ + ZrO_2_	F5	I	−151.4	−153.8	−105.5	±15^c^	903.1	923.2	1032.2	1346.7^a^
R17	Li_2_SiO_3_ + CO_2_ → Li_2_CO_3_ + SiO_2_	F5	I	−85.0	−161.8	−36.7	±6^c^	470.7	479.8	527.6	628.6
R15	CaCO_3_ + MgO + CO_2_ → CaMg(CO_3_)_2_	F6	I	−122.5	−177.3	−69.6	±8^c^	625.4	636.5	694.5	812.7

a Above a participating species' heat-capacity range; shown only as an extrapolation.

b First-order propagated uncertainty of the 1 bar imposed-pressure turnover, 
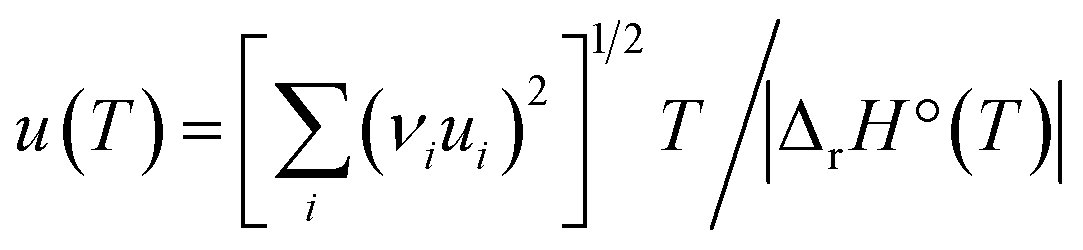
, with *u*_*i*_ the standard formation-enthalpy uncertainty recorded for each adopted anchor in SI Table S1 and treated as independent. One basis is used per species. For magnesite *u*_*i*_ is the ±0.47 kJ mol^−1^ of the Koziol–Newton reversed-equilibrium determination that corroborates the adopted anchor, not the ±8 kJ mol^−1^ of the 1966 JANAF assessment; that wider interval is carried separately as a source-sensitivity scenario (Section 4.3 and SI Section S7.3). It is a source-propagation figure, not a complete uncertainty budget, and it is defined only for the Class I reactions.

c At least one participating species carries no printed uncertainty, so the entry is a lower bound and not comparable with the complete rows; the affected species are listed in SI Section S2.4. p: provisional inputs; R6 and R7 are excluded from the primary aggregate. Their uncertainty entries are withheld because only CO_2_ has a printed uncertainty; the partial calculations remain in SI Section S2.4. The four temperature columns are imposed-CO_2_-pressure turnovers for Class I, not finite-charge 50% or 90% conversion temperatures. Water-dependent reactions are reported separately in Table 2. The numbering is not contiguous: R16 is the excluded Na_2_CO_3_/NaHCO_3_ route of Section 4, which is absent from this table but retained in the SI benchmark register for its recorded comparison rows. The R8 turnover lies inside an unrepresented Na_2_ZrO_3_ transition of about 1.0 kJ mol^−1^ between 1025 and 1110 K (SI Table S1(d)), worth about 7.5 K on that row.

Water-dependent equilibrium quantities, each a restricted, single-reaction equilibrium in the admitted species set of that row rather than a global equilibrium over competing water-mediated reactions. (A) Pressure ratios for reactions with Δ*ν*_g_ = 0, all within the data windows. (B) Selected turnover conventions at imposed *p*_CO_2__ = 0.1 bar, compared with the finite-charge *T*_50_ in an initially pure CO_2_ gas at total pressure 0.1 bar. The latter column allows water to form and is a different boundary condition. Full conventions and validity intervals are in SI Table S2.5^a^

**Table:** 

A. Equilibrium *p*_H_2_O_/*p*_CO_2__ (dimensionless)				
Reaction	Δ*ν*_g_	500 K	700 K	900 K
R3, Ca(OH)_2_	0	2.0 × 10^6^	1.5 × 10^4^	9.0 × 10^2^
R9, Mg(OH)_2_	0	2.9 × 10^2^	21	4.9

a H: above a heat-capacity data window; L: below the 500 K start of the water–vapor polynomial; W: the stated water partial pressure exceeds the saturation pressure at that temperature, so the entry lies inside the liquid–water field and has no vapor-phase meaning. Flagged values are diagnostic extrapolations. “—” denotes no imposed-gas root in 300–2000 K, and “>2000” in the last column means the 50% crossing lies above 2000 K, so equilibrium conversion stays above 50% throughout the tabulated window. R24 cannot proceed in a water-free charge because it consumes water. Part A is quoted to two significant figures because the ratio depends exponentially on Δ_r_*G*°. Section 5.6 gives the temperatures at which the two hydroxides dehydroxylate, which for both lies below the carbonation values in part B.

**Table:** 

B. Temperature (K) under specified gas conditions				
Reaction	Δ*ν*_g_	*p*_H_2_O_ = 1 bar	*p*_H_2_O_ = *p*_CO_2__	Self-generated water, *T*_50_
R3	0	1633.4^H^	—	>2000
R9	0	790.9	1315.6^H^	1315.6^H^
R11	−1/3	376.7^L^	440.4^L^	445.3^L^
R18	−2/3	339.7^L W^	356.3^L^	360.4^L^
R24	−3	415.4^L^	386.5^L^	Not feasible

All 23 reactions are exothermic at 298.15 K and all have Δ*G*°(298.15) < 0. The reaction enthalpy ranges from −33.2 kJ mol^−1^ for chrysotile to −214.7 kJ mol^−1^ for the hydrous anorthite route, which consumes two moles of H_2_O(g) as well as CO_2_ and has the largest entropy loss of the set, −498.6 J mol^−1^ K^−1^. The entropy changes are negative throughout, as expected when a gas is consumed into a solid lattice, but the two reactions that release one mole of H_2_O(g) for every mole of CO_2_ consumed are an order of magnitude smaller in magnitude, portlandite at −16.6 and brucite at −23.1 J mol^−1^ K^−1^, because the gaseous product replaces the gas-phase entropy lost with CO_2_ mole for mole. The corresponding equilibrium pressure ratios and water conventions are therefore reported alongside the temperature-based results.

### 5.2 Gibbs energy along the reaction coordinate

Fig. 2 shows *G*(*T*, *P*, *ξ*) for R2, a Class I reaction, under post-combustion conditions (1 bar, 15% CO_2_ in N_2_) at four temperatures, together with the linear standard part *G*°(*ξ*). At 800 K, Δ*G*° = −52.8 kJ mol^−1^ and *G*(*ξ*) reaches a minimum at *ξ*_eq_ = 0.998, the gap between the two curves widening with *ξ* as depleting CO_2_ raises the mixing penalty. At 1000 K, Δ*G*° = −23.1 kJ mol^−1^ but the minimum has moved inside the domain, to *ξ*_eq_ = 0.626: the standard driving force still favors products, yet the entropy cost of drawing CO_2_ down from 15% to its equilibrium mole fraction cancels most of it. At 1050 K, Δ*G*° = −15.8 kJ mol^−1^ is still negative while *G*(*ξ*) already rises from *ξ* = 0, so equilibrium conversion is zero; at 1150 K, Δ*G*° = −1.3 kJ mol^−1^ and the standard contribution is nearly flat while the total Gibbs energy increases with extent, again with no carbonation. A negative standard Gibbs energy is therefore not sufficient for carbonation in a dilute stream.

**Fig. 2:**
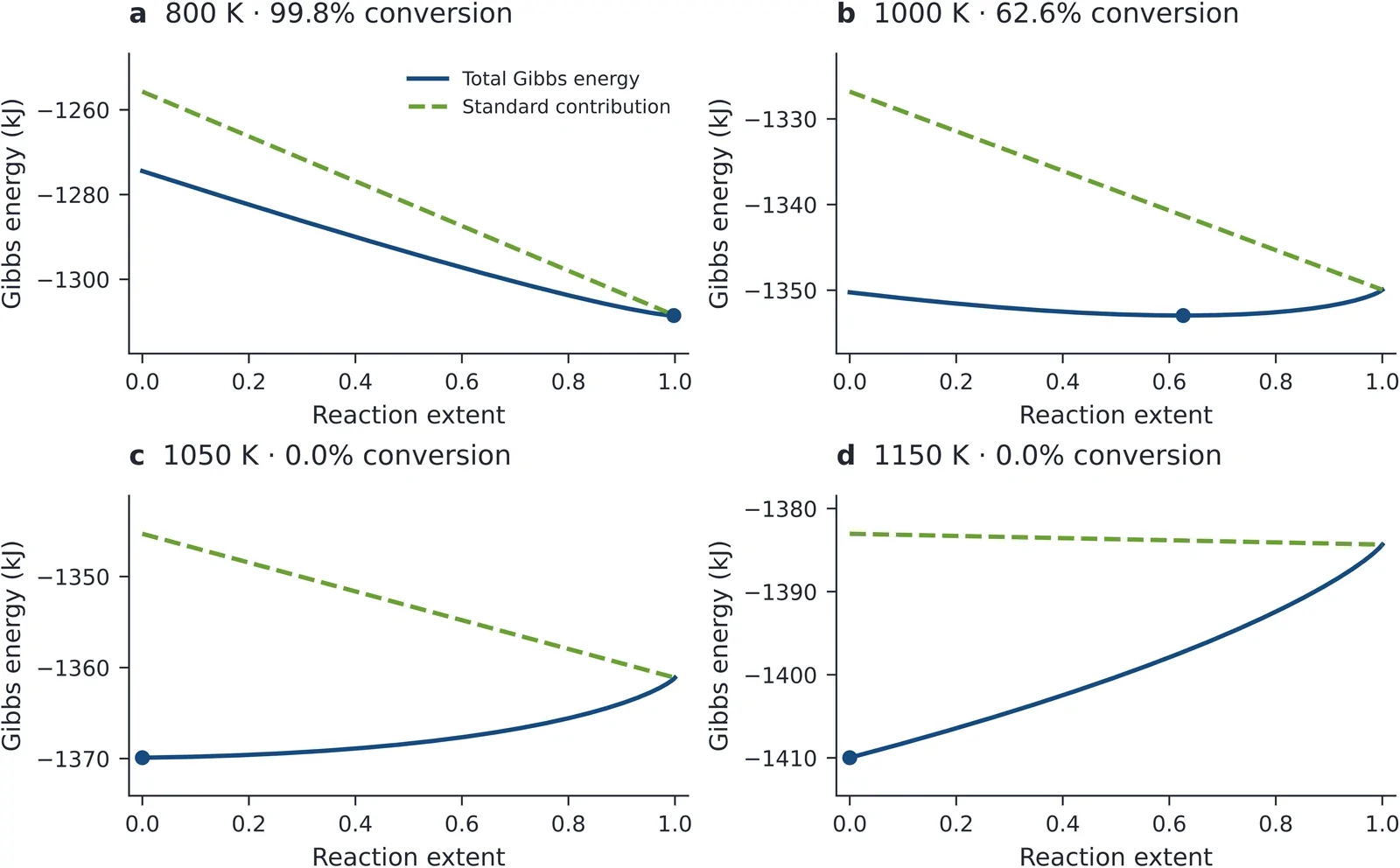
Total Gibbs energy *G*(*T*, *P*, *ξ*) (solid) and standard Gibbs energy *G*°(*ξ*) (dashed) for R2 (CaO + CO_2_ → CaCO_3_) under post-combustion conditions (1 bar, 15% CO_2_ in N_2_) at (a) 800 K, (b) 1000 K, (c) 1050 K and (d) 1150 K. The marker in (b) locates *ξ*_eq_ = 0.626, the 62.6% conversion given in that panel heading. The sequence runs from near-complete carbonation at 800 K, through partial equilibrium at 1000 K, to no carbonation above the turnover temperature. Panel (c) shows that Δ*G*° < 0 does not guarantee appreciable equilibrium conversion in a dilute stream. The dashed reference contribution omits gas mixing; its reaction slope is the standard reaction Gibbs energy. Condensed phases have unit activity and gas standard pressure is 1 bar.

Repeating the construction for all 23 reactions, each near its own turnover point (Fig. S1, SI), gives the same structure: the possibility of an interior minimum extends beyond the CaO system, and for the Class II reactions it survives the removal of the diluent.

### 5.3 Equilibrium conversion maps and sorbent selection

Fig. S5 (SI) gives equilibrium CO_2_ conversion against temperature for post-combustion (15% CO_2_, 1 bar), an undiluted feed carrying no inert (1 bar) and pre-combustion (40% CO_2_, 20 bar) conditions. The contrast between the undiluted and the dilute columns is the practical form of the argument in Section 3.2, and it is a Class I contrast: for the 18 reactions in which CO_2_ is the only gaseous species the undiluted column shows a step, full conversion below *T*_turn_ and none above it, whereas in dilute CO_2_ the steps become graded conversion curves. The four Class II reactions are graded in the undiluted column as well, because the H_2_O(g) they release dilutes the CO_2_ with no inert added, while R24 is stepped there because its stoichiometric humid charge holds the gas composition fixed along the reaction coordinate. CaO holds 96.4% conversion at 900 K under the stated post-combustion feed, falls to 62.6% at 1000 K and reaches zero by 1100 K, a decline that shows why conversion and reaction rate must be considered together in calcium-looping screening.^8,53^

Fig. 3 presents *T*_50_ and *T*_90_ for six representative sorbents at 1 bar. The 90% boundary lies below the 50% boundary because the remaining gas is more depleted at the higher conversion. For CaO at 15% initial CO_2_, *T*_50_ = 1013.4 K and *T*_90_ = 941.6 K, a difference of 71.8 K. Within the modeled charge and phase set, operation below the latter boundary supports at least 90% equilibrium conversion on this branch. It does not establish the residence time or cycling conditions needed to achieve that conversion.^8,53^ Full target tables and validity flags are supplied in SI Section S7; the 23-reaction conversion profiles are retained in Fig. S5.

**Fig. 3:**
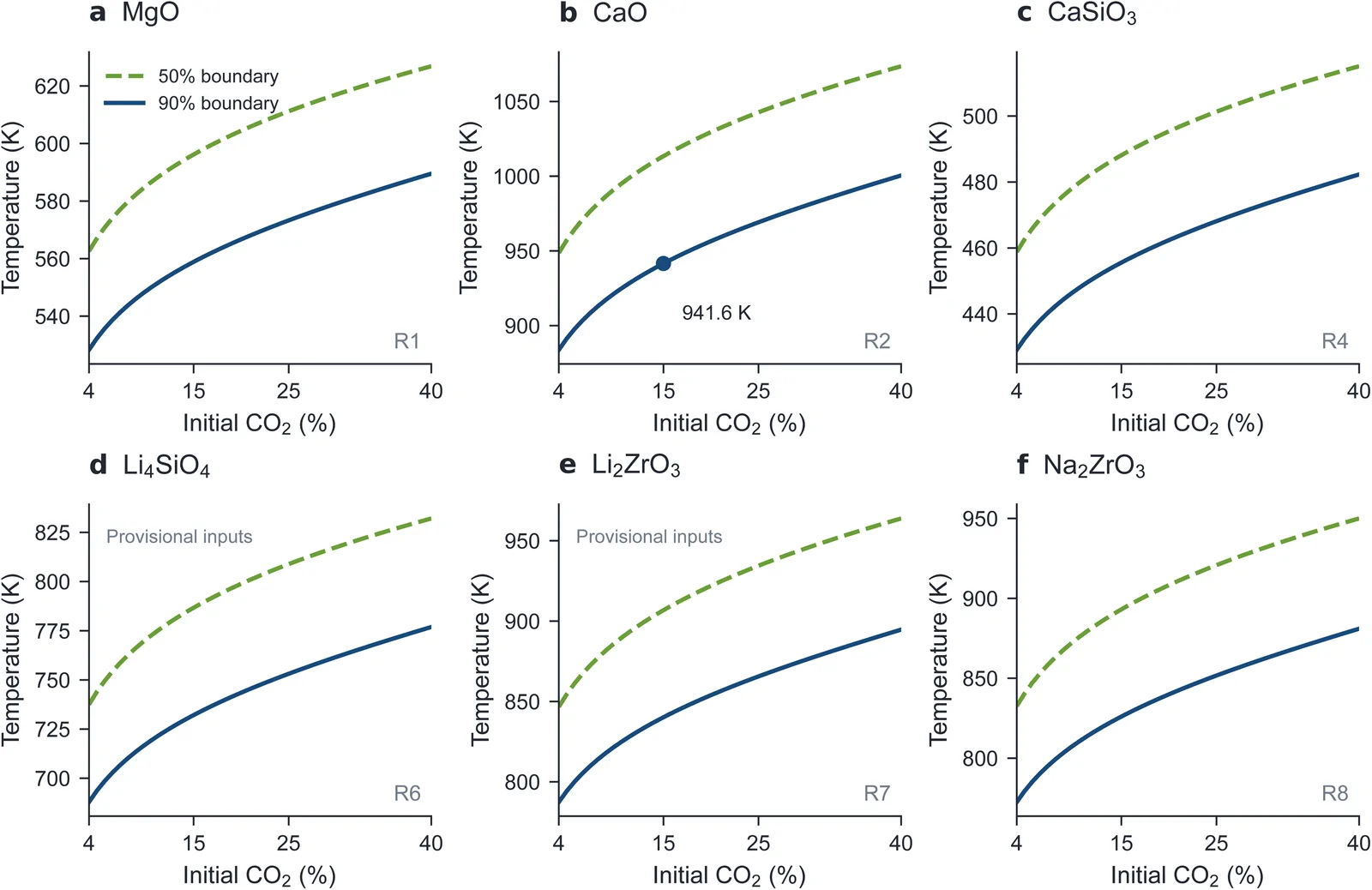
The panels show (a) MgO (R1), (b) CaO (R2), (c) CaSiO_3_ (R4), (d) Li_4_SiO_4_ (R6), (e) Li_2_ZrO_3_ (R7) and (f) Na_2_ZrO_3_ (R8). Condensed reactants are stoichiometric and the gas composition evolves with conversion. Only boundaries within all participating heat-capacity windows are drawn. R6 and R7 have provisional reference-state inputs and are excluded from the primary aggregate. The panels use different temperature scales to resolve each material's operating window. The marked CaO example gives *T*_90_ = 941.6 K at 15% initial CO_2_. These are restricted, single-reaction equilibrium screening boundaries for the specified inventories and admitted species, not global equilibria over competing reactions and not reactor performance predictions.

### 5.4 The dilution penalty across the reaction set

The pure-to-dilute comparison is not specific to any one sorbent, and it belongs to Class I. Taking the temperature at which equilibrium conversion passes 50% as a screening measure, and comparing an undiluted CO_2_ feed at 1 bar with 4% CO_2_ at 1 bar, the comparison is defined for all 18 Class I reactions and all 18 respond. R6 and R7 have unresolved reference-state inputs and are excluded from the primary aggregate. Over the remaining 16 the shift has a median of 93.8 K and a maximum of 210.4 K for CaO. Including the two provisional cases gives the full-set diagnostic median of 100.5 K. The pure-feed member is a degenerate step boundary, whereas the dilute-feed member is the graded 50% conversion point. Reaction by reaction (Fig. S2, SI) the pattern is monotonic in CO_2_ mole fraction: for CaO that temperature falls from 1159.0 K in pure CO_2_ to 1013.4, 992.1 and 948.6 K at 15%, 10% and 4% CO_2_, and for MgO from 669.5 K to 596.2, 585.2 and 562.6 K.

Each source change is applied consistently to both feeds and to every affected reaction. The selected magnesite anchors give primary medians of 93.8–94.3 K; the broader JANAF enthalpy interval gives 92.4–95.2 K with other inputs fixed. The corresponding full-set diagnostic ranges are 100.5–101.4 and 96.5–102.7 K. These are conditional source sensitivities, not confidence intervals. SI Table S7.8 reports both populations for every scenario.

The median is less sensitive than some individual reactions (Fig. S6). Only seven of the 16 primary Class I reactions contain magnesite, so variation of that anchor affects a minority of the population. The leading-order expression in Section 3.3 also explains why reactions consuming one mole of gas exhibit similar relative shifts.

Among the provisional cases, the 229.8 K reference shift for Li_2_ZrO_3_ combines a pure-gas boundary at 1076.5 K above carbonate melting with a dilute-gas boundary at 846.7 K below it. Continuing solid carbonate reduces the shift to 200.9 K. Substituting Duan's paired 300 K reaction enthalpy and Gibbs-energy anchor, while retaining the present heat capacities, gives 150.9 K; the HSC-Chemistry column printed beside it in the same source gives 229.1 K.^27^ Thus the R7 shift spans 150.9–229.8 K across these conditional diagnostics. The anchor substitution is not a full reproduction of the first-principles calculation, and the reference maximum is not a source-independent material property.

### 5.5 Humidity and zero net gas stoichiometry

For R3 and R9, Δ*ν*_g_ = 0, and the equilibrium pressure ratio is more informative than an isolated turnover temperature. At 700 K, (*p*_H_2_O_/*p*_CO_2__)_eq_ is 1.5 × 10^4^ for portlandite and 21 for brucite. The ratio depends exponentially on Δ_r_*G*°, so it is quoted to two significant figures throughout. These ratios follow directly from the standard reaction Gibbs energy and do not require an arbitrary imposed water pressure. Their temperature dependence and the principal water conventions are shown in Table 2. The *p*_H_2_O_ = 1 bar column of that table is the reporting convention recorded in the locators of the published values compared against in Section 5.8. It is a steam-rich condition relative to *p*_CO_2__ = 0.1 bar and is not a representative flue-gas composition.

A large sensitivity attaches to these reactions, this time to the reporting convention rather than to the data. The brucite turnover at *P*_CO_2__ = 0.1 bar is 790.9 K at *p*_H_2_O_ = 1 bar and 1315.6 K at *p*_H_2_O_ = 0.1 bar, a difference of 524.7 K between two different gas conventions, and portlandite has no turnover below 2000 K except in a steam-rich atmosphere. At the unit-ratio condition *p*_H_2_O_ = *p*_CO_2__ the quotient of a Δ*ν*_gas_ = 0 reaction is pressure independent and brucite's turnover is 1315.6 K at every CO_2_ pressure, a pressure-independent comparison convention under the ideal-gas assumption. The 1315.6 K value exceeds the 900 K brucite heat-capacity window and is a diagnostic extrapolation. The spread over the five conventions of SI Table S2.5 reaches 653.7 K for brucite at *p*_CO_2__ = 0.15 bar. For a closed charge, water is produced or consumed according to stoichiometry instead of being held at an imposed pressure. The matched-humidity calculations in SI Section S7 and Fig. 1d compare R9 and R24 under the same actual initial gas composition. Insufficient initial water can prevent a target conversion for R24 regardless of temperature.

### 5.6 Competing reactions outside the restricted equilibrium

Every value in Tables 1 and 2 is a restricted single-reaction equilibrium, so none of them tests whether a competing reaction reaches its own crossover first. Three such competitors can be assembled from the species set of this work and are quantified in Table 3. Subtracting R1 from R9 gives Mg(OH)_2_ → MgO + H_2_O(g), whose crossover at *p*_H_2_O_ = 1 bar is 537.6 K. The brucite carbonation turnover of 790.9 K at *p*_CO_2__ = 0.1 bar therefore describes a reactant that the same database places outside its stability field under that fixed-water convention. The same construction from R2 and R3 puts portlandite dehydroxylation at 795.3 K, far below the 1633.4 K reported for R3. This provides a possible contribution to benchmark B008, where the published 600 K sits 191 K below the restricted carbonation turnover; a matched multireaction calculation would be needed to establish the cause.

**Table 3:** Competing reactions assembled from the same species set, with the temperature at which each crosses its own equilibrium. Dehydroxylation crossovers are the roots of Δ_r_*G*°(*T*) + *RT* ln(*p*_H_2_O_/*P*°) = 0; the double-carbonate rows give the turnover temperature of the same carbonation reaction when its CaCO_3_/MgCO_3_ product pair is replaced by dolomite. Full rows are supplied with the SI data

Competing process	Condition	*T*/K
Mg(OH)_2_ → MgO + H_2_O(g)	*p*_H_2_O_ = 1 bar	537.6
Ca(OH)_2_ → CaO + H_2_O(g)	*p*_H_2_O_ = 1 bar	795.3
CaCO_3_ + MgCO_3_ → CaMg(CO_3_)_2_	Condensed phases	No root
R13 with a dolomite product	*p*_CO_2__ = 1 bar	474.9
R21 with a dolomite product	*p*_CO_2__ = 1 bar	591.0
R22 with a dolomite product	*p*_CO_2__ = 1 bar	661.8

Subtracting R1 from R15 gives CaCO_3_ + MgCO_3_ → CaMg(CO_3_)_2_, with Δ_r_*G*° = −5.4 kJ mol^−1^ at 298.15 K and no crossing below 2000 K, so the ordered double carbonate is the more stable product assemblage throughout. R13, R21 and R22 are nevertheless written to produce CaCO_3_ and MgCO_3_ as separate pure phases. Replacing that pair by dolomite, as far as the Ca to Mg ratio of each reaction allows, raises their 1 bar turnover temperatures by 15.4, 9.2 and 6.7 K respectively. These shifts quantify this particular product-phase substitution; additional solid-solution models could alter the boundaries further. The shifts are larger than the gas-phase fugacity correction reported in Section 5.7.

These crossovers are computed from the same Shomate functions and anchors as the screen itself and inherit its qualifications. They are stated so that a reader can see which reported boundaries lie in a field where an alternative phase assemblage becomes thermodynamically favorable. They do not constitute a multireaction equilibrium calculation, which would require the composition space the formalism deliberately excludes.

### 5.7 Effect of total pressure

For Class I, raising the total pressure works in the opposite direction to dilution and can be used to recover part of the loss. The span in turnover temperature between 0.15 and 20 bar CO_2_ has a median of 165 K across the full-set diagnostic population of 18 Class I reactions, including provisional R6 and R7, the population for which that span is a function of *P*_CO_2__ alone, and reaches 738.5 K for Li_2_ZrO_3_, where the upper value lies above the melting point of the Li_2_CO_3_ product and outside the heat-capacity validity window. These extrapolated values are not operating recommendations. Fugacity effects were checked separately at the finite-charge 50% boundaries for MgO, CaO, Li_4_SiO_4_, Li_2_ZrO_3_ and Na_2_ZrO_3_, with total pressures of 0.15, 1 and 20 bar and initial CO_2_ fractions of 0.15, 0.40 and 1.

Across these 45 cases, replacing ideal partial pressures with PR fugacities changes *T*_50_ by at most 1.65 K; restricting both roots to their heat-capacity windows reduces the maximum to 0.85 K. The CO_2_ fugacity coefficient at the roots ranges from 0.99993 to 1.00520 (Fig. S7). The condensed-phase pressure term neglected in eqn (5) can be sized from the same volume data used for the 600 bar diagnostic of Section 4.3. For the magnesite reaction it is 0.032 kJ mol^−1^ at 20 bar and moves that turnover by 0.23 K. These comparisons support the ideal-gas approximation at the tested screening boundaries. They are not a uniform error bound over 300–2000 K: the 45 tested roots lie between 551 and 1675 K, and pure CO_2_ at 300 K and 20 bar has a substantially larger departure, with *ϕ* approximately 0.896. The coldest roots of the screen fall below the tested range and were bounded separately. For the five lowest Class I turnovers at 20 bar, from 489.0 K for R14 to 580.1 K for R5, the pure-CO_2_ fugacity coefficient lies between 0.982 and 0.993. Imposing the 300 K value 0.8955 instead, as a deliberately conservative bound, moves those turnovers by at most 4.23 K. Water-containing calculations and all critical constants, mixing rules and flags are reported in SI Section S7.

### 5.8 Benchmark distribution and numerical verification

Each published turnover temperature, decomposition equilibrium and standard reaction property located for the covered reactions was collected with a source, a DOI and a page or table locator, and joined to the computed results. The benchmark register holds 112 rows from 11 source documents; 100 receive a computed value and 59 enter the deviation statistics, 33 of temperature and 26 of energy, covering 17 of the 23 reactions. Rows are excluded with a stated reason: measurements far above the pressure range in which the ideal-gas treatment applies, kinetic uptake windows that are not equilibrium quantities, phases outside the species set, and three rows in which the source quotes for one phase a value belonging to another.

Each row carries a lineage class. Independent first-principles and independent experimental rows distinguish density functional theory with lattice phonon dynamics from direct measurements; source and phase dependencies are assessed separately for each record. Shared-data-lineage rows come from a compilation that also supplies inputs to this work, and secondary-source rows were read from a paper quoting another primary. The distinction changes the meaning of every statistic computed from these rows. All 33 temperature deviations are shown individually in Fig. 4, including the 675.4 K R7 discrepancy outside the heat-capacity window. With only 11 first-principles temperature comparisons, a pooled median is not used as a measure of predictive accuracy. The complete records, descriptive statistics and exclusion reasons remain in SI Section S3.

**Fig. 4:**
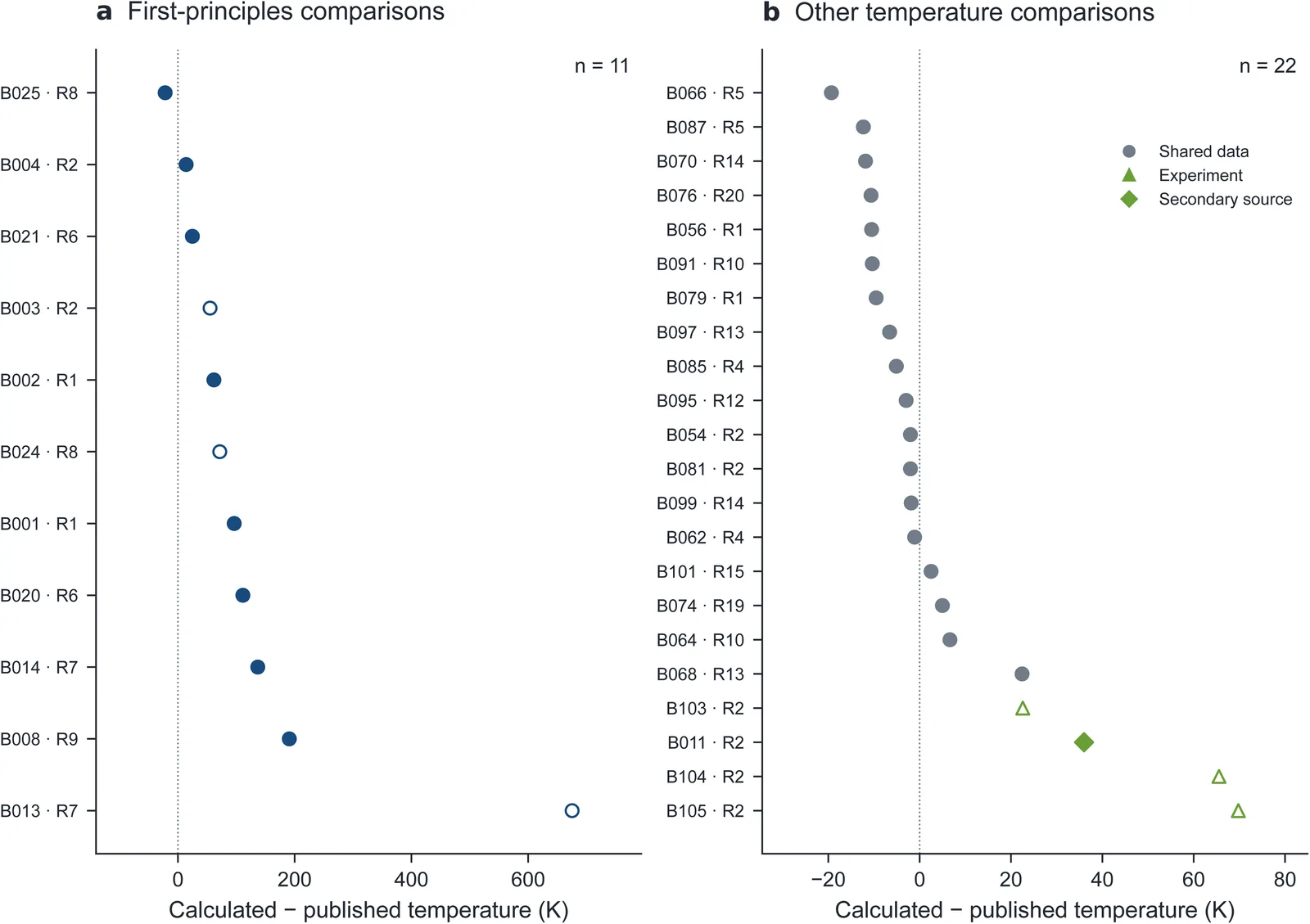
All 33 retained temperature comparisons, labeled by benchmark and reaction identifiers linked to SI Table S3.1. (a) The 11 first-principles comparisons. (b) The remaining shared-data, experimental and secondary-source comparisons. Positive deviations mean the present temperature is higher. For experimental brackets, the signed deviation is measured to the nearest bracket edge and is zero within the bracket. Open symbols identify temperatures outside a participating heat-capacity interval; these remain visible for diagnosis rather than being removed from the distribution. Shared-lineage comparisons assess source consistency and are not independent validation.

One lineage is not apparent from the citation alone. Lackner *et al.*^2^ state that their Table 2 is based on their ref. 25, which is Robie, Hemingway and Fisher, U.S. Geological Survey Bulletin 1452,^54^ the earlier volume of the series that Bulletin 2131 continues.^43^ Two consequences follow: Lackner *et al.* and Robie and Hemingway are not two independent sources, and because the 298 K frame of most silicate species here comes from Bulletin 2131, a comparison against either tests this work against the compilation supplying its own inputs.

The close shared-lineage agreement verifies internal consistency with the source thermochemistry, whereas the wider spread against independent calculations and experiments can reflect differences in underlying datasets, phase assumptions and reference-state choices. The separation is important: a pooled statistic would present the consistency check as evidence of predictive accuracy. No claim of predictive agreement within 25 K is made here, and none is supported by these data.

The magnesite reference state illustrates why the source must be identified. The NBS anchor is 15.9 kJ mol^−1^ less negative than the adopted value; substituting it lowers the turnover temperatures of the seven magnesite-forming Class I reactions by 24–93 K at 1 bar and the brucite value by 333 K at *p*_CO_2__ = 0.1 bar. The adopted anchor is supported by the Koziol–Newton reversal assessment and the independent aqueous determination, with the qualifications set out in Section 4.3. Disagreement with the assessed-database column traced to the NBS anchor is attributed to that anchor; the separately calculated first-principles columns are not described as sharing it. Phase structures, entropies and heat capacities also differ, so this is not a finding that those calculations are otherwise erroneous.

Available experimental comparisons are at elevated pressures or in pure CO_2_ at 1 bar, and none is at a dilute, near-atmospheric feed like the conditions used in the target-conversion maps. Excluding liquid carbonate can reduce a discrepancy without reproducing all structures assumed in a first-principles study.

Agreement between the two formulations of Section 2.4 is within numerical tolerance. Across 368 equilibrium states spanning all 23 reactions, 500–1300 K, total pressures of 1 and 20 bar and initial CO_2_ mole fractions of 1.0, 0.5 and 0.2, the largest discrepancy in the equilibrium extent was max|Δ*ξ*_eq_| = 4.44 × 10^−7^ and in the total Gibbs energy 4.4 × 10^−6^ kJ on values of order 10^3^ kJ. Turnover temperatures agreed to max|Δ*T*_turn_| = 2.13 × 10^−9^ K over the 89 reaction–pressure combinations possessing a turnover in 300–2000 K, the remaining 3 of 92 returning no root by either method. A separate affinity-based reconstruction reproduces the 1495-state grid within 2.16 × 10^−7^ in extent. These are computed maxima; detailed checks are given in SI Table S4.1. Because both formulations read the same thermochemical database, this verifies the numerical consistency of the implementation and of the reaction-coordinate parameterisation to the reported tolerance, and is not a test of the accuracy of the underlying data, which is addressed separately through the source sensitivities and benchmark comparisons.

## 6 Discussion

### 6.1 What changes for screening practice

The two regimes call for different design variables. In a Class I reaction with pure CO_2_ the turnover temperature defines a step boundary, conversion being complete below it and zero above it; with a dilute feed the turnover evaluated at the initial *p*_CO_2__ marks the upper edge of a graded transition, and the shape of the conversion curve inside that band sets the operating point for a specified target. No unique extent is selected exactly at the pure-feed step. A screening calculation carried out in pure CO_2_ and then applied to a dilute charge overstates the accessible window at the 50% conversion point by a median of 93.8 K over the 16 reactions in which CO_2_ is the only gaseous participant and the inputs are located, and by up to 210.4 K. R6 and R7 remain provisional and are excluded from that aggregate.

Five working consequences follow for anyone screening sorbents. Establish first which species occupy the gas phase along the reaction coordinate, because that decides whether an undiluted calculation is a pure-CO_2_ calculation at all and whether a turnover temperature is defined by *P*_CO_2__ alone. Compute the equilibrium conversion at the actual gas composition and total pressure rather than inferring feasibility from the sign of Δ*G*°. For CaO at 15% CO_2_ and 1 bar, the 90% boundary lies 71.8 K below the 50% point. Do not rank candidates on Δ*G*° at 298 K, which reorders the set relative to a ranking on equilibrium conversion at the intended operating temperature. Carry the validity range of the heat-capacity data alongside every temperature quoted, because several of the most attractive-looking turnover temperatures in Table 1 lie outside it. And keep self-consistency separate from independent evidence when quoting agreement with the literature.

### 6.2 When the turnover temperature is ill conditioned

Differentiating eqn (11) at fixed feed and pressure gives the local sensitivity in eqn (13). At a root, its denominator reduces to −Δ_r_*H*°(*T*_*c*_)/*T*_*c*_: small reaction enthalpy, not zero net gas stoichiometry alone, makes a temperature boundary weakly conditioned. For comparison, the standard reaction enthalpies at 298.15 K are −33.2, −35.5 and −69.1 kJ mol^−1^ for R11, R9 and R3; the sensitivity uses the temperature-dependent enthalpy at each root. A separate sensitivity attaches to the reporting convention rather than to the data. The humidity comparisons and equilibrium pressure ratios in Section 5.5 distinguish these effects without assigning a unique intrinsic temperature to a water-dependent equilibrium.

### 6.3 What the benchmark comparison establishes

Two statements are supported: the implementation is numerically consistent within its own formalism to the reported tolerance; and the located thermochemical inputs reproduce their cited source functions within the recorded fit residuals. The magnesium comparisons support the selected anchor range with different qualifications: the Koziol–Newton reversal is at high pressure and shares thermochemical information, while the independent aqueous comparison distinguishes the directly reported properties from a reconstruction using CODATA ions (Section 4.3).

Three are not supported and are not made. There is no demonstration of predictive agreement within 25 K against independent sources. There is no experimental validation at flue-gas conditions: the available experimental comparisons concern elevated pressures or pure CO_2_, rather than dilute, near-atmospheric feeds. And no comparison is described as independent when it shares a data lineage with the inputs. A further open comparison is with a condition-matched FactSage, HSC Chemistry or Thermo-Calc equilibrium calculation; the fourteen benchmark rows drawn from those codes contain standard 300 K properties rather than equilibrium temperatures.

### 6.4 Relation to established equilibrium solvers

The calculation reported here is narrow by design. General solvers such as FactSage,^20^ HSC Chemistry,^21^ Cantera^23^ and OpenCalphad^24^ minimize the Gibbs energy over the admitted species in a multi-phase system and remain the right choice when solution phases, competing reactions or unusual chemistries are involved. The extent-of-reaction treatment provides interpretability for one question: for a single stoichiometric solid–gas reaction it produces the entire *G*(*ξ*) profile, so the reason a reaction stalls at partial conversion, and the class it belongs to, can be read off the curve, with each parameter carrying its source and validity qualification.

### 6.5 Limitations

(1) The gas phase is treated as ideal; representative dry-gas fugacity checks support the tested screening boundaries, but do not establish a uniform error bound over the complete temperature range or a calibrated description of humid mixtures. (2) Condensed phases are pure and at unit activity, so departures from stoichiometry and impurity effects in real feedstocks are not represented. (3) Solid solutions are not represented, which matters for mixed-metal sorbents and for natural olivines and plagioclases. (4) Only the equilibrium state is computed; no rate law is applied. (5) Diffusion and mass transfer resistance, including product-layer diffusion, are outside the treatment. (6) Sintering and pore closure, which reduce cyclic capacity in real sorbents,^55^ are not modeled. (7) One reaction coordinate is solved at a time, so competing or sequential equilibria, such as simultaneous carbonation and dehydroxylation under humid conditions, are not resolved. Water is solved within that reaction's inventory, not across all competing pathways. (8) No condition-matched low-pressure experimental equilibrium benchmark exists for most of the set, so for the majority of the 23 reactions the comparison rests on first-principles values or on shared-lineage compilations rather than on measurement at the conditions of interest.

The restriction is quantified in Section 5.6. At fixed *p*_H_2_O_ = 1 bar, the dehydroxylation crossovers of brucite and portlandite lie below their restricted carbonation turnovers under the tabulated gas conventions. Replacing a calcite/magnesite product pair by dolomite moves three turnover temperatures by 7–17 K at 10 bar and 7–15 K at 1 bar. These are competing-reaction diagnostics, not global equilibrium calculations. Thermochemical sensitivities are also conditional because complete input uncertainties and covariances are unavailable; R6 and R7 remain provisional.

### 6.6 Further work

Coupling the equilibrium extent to a rate expression would turn the conversion maps into combined thermodynamic and kinetic screens, and a vector of extents in place of a single extent variable would let the competing carbonation and dehydroxylation equilibria of the Class II and Class III sorbents be solved together over the expanded species set. Measurements would remove named uncertainties: a heat capacity for nahcolite would admit the carbonate-looping route excluded here, traceable formation properties and heat capacities would admit Li_8_SiO_6_, and a modern reversal of a low-pressure carbonation equilibrium would supply the condition-relevant experimental anchor the comparison set lacks.^56,57^ A finite-charge screen does not reproduce a continuously replenished reactor: kinetic and flow models must retain the intended gas supply and residence-time basis.

## 7 Conclusions

The extent-of-reaction form of Gibbs energy minimization separates equilibrium regimes that a table of standard Gibbs energies cannot distinguish, and gas-phase stoichiometry decides which regime a reaction is in for a stated initial charge. Where CO_2_ is the only gaseous participant, 18 of the 23 reactions here, an undiluted feed makes the total Gibbs energy linear in the extent of reaction and equilibrium binary, full conversion below the turnover temperature and none above it, except at the transition itself; with a diluent present CO_2_ is depleted as the reaction advances, the gas-phase mixing entropy curves the profile and equilibrium can settle at partial conversion. The four reactions that release H_2_O(g) are curved with no diluent present; the two whose net change in the number of gas moles is zero do not respond to inert dilution, their equilibrium being exactly independent of inert loading and of total pressure under ideal-gas assumptions and unchanged reactive inventories, while humidity remains consequential. The one that consumes H_2_O(g) is a humid-gas case throughout. Standard-state favorability and equilibrium conversion are therefore not interchangeable in a dilute charge, and a screening temperature carries no meaning apart from the gas composition it belongs to.

Over the 16 Class I reactions with located reference-state sources, screening in pure CO_2_ overstates the feasible window at 4% CO_2_ and 1 bar by a median of 93.8 K and by as much as 210.4 K. This compares a pure-feed step with a dilute-feed 50% conversion point. Conditional selected-source calculations give 93.8–94.3 K; the wider JANAF magnesite enthalpy interval gives 92.4–95.2 K with other inputs fixed. R6 and R7 remain provisional and do not determine this aggregate. For CaO at 15% CO_2_ and 1 bar, the 90% boundary is 941.6 K. The thermochemistry is traceable at parameter level: each of the 104 entries describing the 32 species carries a source or an unresolved-source flag, a validity range and a method flag; no heat capacity is estimated by oxide additivity. Only three Class I reactions have printed formation-enthalpy uncertainties for every participating species, giving conditional propagated contributions of 3.4–13.8 K; the other fifteen have incomplete coverage.

Verification and benchmarking answer different questions and are reported separately. An independently formulated element-potential minimization, sharing the database but no part of the reaction-coordinate machinery, reproduces the equilibrium extents to 4.44 × 10^−7^ and the turnover temperatures to 2.13 × 10^−9^ K, which verifies the numerical consistency of the implementation and of the reaction-coordinate parameterization to the reported tolerance and says nothing about the data. Source sensitivities, competing-reaction crossovers and physical comparisons expose larger, separate limitations. No claim of complete experimental validation is made. The study is restricted to pure-phase, single-reaction equilibria and excludes the Na_2_CO_3_/NaHCO_3_ route and Li_8_SiO_6_ because sufficiently complete, traceable inputs were not assembled.

For practice, the recommendation is to screen at the actual gas composition and pressure, with the gaseous species the reaction itself produces or consumes included in that composition, and with inputs whose sources and validity ranges are carried through to the answer. The framework returns restricted equilibrium limits rather than rates, cyclic capacities or reactor productivities, and those limits support candidate and condition selection for subsequent experiments.

## Author contributions

Abdulaziz A. Alturki: conceptualization, methodology, software, validation, formal analysis, investigation, data curation, visualization, writing (original draft), writing (review and editing).

## Conflicts of interest

There are no conflicts to declare.

## Supplementary Material

RA-016-D6RA07742K-s001

## Data Availability

The thermochemical provenance data, reaction definitions, equilibrium results, benchmark comparisons, numerical verification and sensitivity results supporting this article are provided in the supplementary information (SI) and accompanying CSV tables. The manuscript and SI document the equations and numerical procedures for independent implementation and verification. The Python scripts used for the reported calculations will be provided upon request. Supplementary information: parameter provenance, reaction definitions, equilibrium states and turnover conventions (Sections S1 and S2); benchmark records and numerical cross-checks (Sections S3 and S4); SI figures (Fig. S1–S7); and source sensitivity, fugacity comparisons, humidity cases and target-conversion results (Section S7). Tabulated inputs and numerical outputs are also supplied in machine-readable CSV files. See DOI: https://doi.org/10.1039/d6ra07742k.
