# Supplementary material for: Thermodynamic screening of solid–gas CO2 mineral carbonation: a provenance-traceable Gibbs-energy framework across 23 reactions
Source: RSC Adv. 2026 Oct 21;16(48):65080–97. doi: 10.1039/d6ra07742k (PMC13685540; doi:10.1039/d6ra07742k)
Supplement: Supplementary file 1 [file RA-016-D6RA07742K-s001.pdf]

Supplementary Information (SI) for RSC Advances.
This journal is © The Royal Society of Chemistry

Electronic Supplementary Information

Thermodynamic screening of solid–gas CO_2 mineral carbonation:
a provenance-traceable Gibbs-energy framework across 23 reactions

Abdulaziz A. Alturki
Department of Chemical and Materials Engineering, Faculty of Engineering, King Abdulaziz University, Jeddah 21589, Saudi Arabia
Center of Research Excellence in Renewable Energy and Power Systems, King Abdulaziz University, Jeddah 21589, Saudi Arabia
E-mail: aaaaltrki@kau.edu.sa ORCID: 0000-0001-5901-7323

Contents

Item	Description
S1	The thermochemical database and its provenance. Table S1: 104 parameters across 32 species, each with its value, validity interval, method flag, source and the table or page within that source; Table S1(b): the source key; Table S1(c): the full-precision calibrated Shomate coefficients; and Table S1(d): scientifically material qualifications carried with the inputs.
S2	The complete computed data set. Table S2.1 the 23 reactions, their heat-capacity validity windows and their gas-stoichiometry class; Table S2.2 the full sweep, 23 reactions $\times$ 13 temperatures $\times$ 5 gas conditions; Table S2.3 the 500 K conversion selection table; Table S2.4 turnover temperatures at four CO_2 partial pressures on two bases; Table S2.5 the five reactions involving H_2O(g) under five stated water conventions; Table S2.6 the 50% conversion temperatures used for the dilution-shift statistics.
S3	The benchmark atlas. Table S3.1 all 112 published values, one row each, with the computed value, the deviation and the benchmark class; Table S3.2 the 13 comparison rules and source qualifications; Table S3.3 the per-class statistics.
S4	Numerical cross-check against an independently formulated element-potential minimization. Tables S4.1 to S4.3.
S5	Supporting figures. Fig. S1 $G(\xi)$ profiles for all 23 reactions; Fig. S2 the concentration and pressure effect reaction by reaction; Fig. S3 standard reaction properties against temperature; Fig. S4 turnover temperatures against their validity windows; Fig. S5 conversion maps.
S6	Candidate reactions and phases outside the admitted data set.
S7	Target-conversion temperatures, matched humidity, paired thermochemical source sensitivities and gas-fugacity sensitivities; Tables S7.1–S7.7 and Figs. S6–S7.

The tables, figures and accompanying supplementary CSV files document the thermochemical inputs, reaction definitions, computed equilibria, literature comparisons and numerical verification checks. Section S7 gives the target-conversion and sensitivity calculations with their feed definitions and validity flags.

S1. The thermochemical database and its provenance

The database holds 32 species, two gaseous (CO_2, H_2O) and 30 condensed. Each entry carries a standard formation enthalpy $\Delta_f H^\circ$(298.15 K), a standard entropy S°(298.15 K), a phase tag and one or more Shomate intervals, in which

$$C_p^\circ(T) = A + Bt + Ct^2 + Dt^3 + E/t^2, \qquad t = T(\mathrm{K})/1000,$$

with the integration constants F and G calibrated to the specified enthalpy increment and absolute entropy at 298.15 K. Enthalpy and entropy are continuous at ordinary coefficient boundaries. At phase transitions their jumps satisfy $\Delta H_{\mathrm{tr}} = T_{\mathrm{tr}}\Delta S_{\mathrm{tr}}$, so Gibbs energy remains continuous. Table S1(c) gives the calibrated coefficients. The species quantity used in numerical minimization is

$$g^\circ_{i,\mathrm{ref}}(T) = \Delta_f H^\circ_i(298.15) + [H^\circ_i(T) - H^\circ_i(298.15)] - TS^\circ_i(T). \tag{1}$$

It differs from the standard Gibbs energy of formation at T by the elemental reference contributions; these cancel in each element-balanced reaction. Accordingly, $\sum_i \nu_i g^\circ_{i,\mathrm{ref}} = \Delta_r G^\circ$.

The magnesite input combines JANAF enthalpy and heat capacity with the later calorimetric entropy, rather than using a single internally assessed data set. The JANAF entry was last reviewed in December 1966 and gives $\Delta_f H^\circ = -1111.7 \pm 8$ kJ mol^{-1}. The numerical anchor is -1111.69 kJ mol^{-1} and the entropy is 65.09 J mol^{-1} K^{-1}. Source alternatives and the interpretation of the reported uncertainty are evaluated separately in Section S7.3.

Table S1 is the provenance table itself: one row per parameter, 104 rows in all. Each row states the parameter, its value, the temperature interval over which it is valid, the method by which it entered the database, the source, the table or page within that source, and, for coefficients obtained by fitting, the largest residual against the source data. The five method flags are `JANAF-tabulated` (25 rows), `assessed-compilation` (53), `fitted-to-published-Cp` (20), `calorimetry` (3) and `unlocated` (3). To avoid introducing estimated heat-capacity trends into a screening analysis, heat-capacity functions were taken from measured, assessed or fitted source data, so the database contains no Neumann–Kopp oxide-additivity estimates. The source-verification criteria and the excluded candidates are described in Section S6.

Source attribution and provisional inputs. The 64 formation-enthalpy and entropy entries are attributed individually to the compilation that prints the adopted value. The three unlocated entries are the Li_4SiO_4 formation enthalpy and entropy and the Li_2ZrO_3 entropy. They are explicit provisional assumptions used only for R6 and R7, which are excluded from the primary screening aggregate. Alternative reaction-level anchors and competing compilation values are evaluated in Section S7.3. Table S7.8 separates the 16-reaction primary Class I population from the complete 18-reaction diagnostic population.

The fit residuals in the last numeric column are the largest absolute percentage deviation of the fitted heat capacity from the source data over the stated interval. The largest of them is 0.652% for talc, against a gate of 1%. Where a source prints both a heat-capacity table and a heat-capacity equation, fitting the table and then comparing with the printed equation checks the extraction chain independently. The 0.0014% brucite residual is the fit-versus-table residual already reported in the Fit dev. column, not an equation-versus-fit agreement. The separate water-vapor check is a different construction again: the 500–1700 K H_2O(g) range, extrapolated below its lower limit, reproduces the tabulated heat capacity to 0.0041% at 300 K and 0.0033% at 400 K, which is the basis for the ten `XL` flags of Table S2.4.

Table S1 Per-parameter provenance of the 32-species thermodynamic database: 104 parameters, each with its value or coefficient-table cross-reference, validity interval, method flag, source and source locator. Source keys and identifiers are defined in Table S1(b), the full-precision calibrated Shomate coefficients are given in Table S1(c), and scientifically material qualifications are retained in Table S1(d). Locators beginning UNLOCATED mark the three anchors that none of NIST-JANAF, Robie and Hemingway, the NBS Tables or CODATA prints; they are retained because no better sourced value exists for those phases.

Species	Phase	Parameter	Value	Units	Validity / K	Method flag	Src	Fit dev. / %	Locator within the source
CO_2	g	$\Delta_f H^\circ(298.15)$	-393.5220	kJ/mol	298.15	JANAF-tabulated	S1	–	NIST-JANAF table C-095, CO2(g), 298.15 K row: dfH(298.15) = -393.522 kJ/mol, printed to the adopted digits. CODATA 1989 gives -393.51 +/- 0.13
CO_2	g	$S^\circ(298.15)$	213.7850	J/(mol K)	298.15	assessed-compilation	S8	–	CODATA Key Values table, CO2(g): S(298.15) = 213.785 +/- 0.010 J/(mol K); this is the printed source of the adopted 213.785. NIST-JANAF (C-095) prints 213.795 and the NIST WebBook summary 213.79
CO_2	g	Shomate range 1	Table S1(c)	Cp in J/(mol K), T/1000	298–1200	JANAF-tabulated	S1	–	Gas Phase Heat Capacity (Shomate Equation), 298-1200 K
CO_2	g	Shomate range 2	Table S1(c)	Cp in J/(mol K), T/1000	1200–6000	JANAF-tabulated	S1	–	Gas Phase Heat Capacity (Shomate Equation), 1200-6000 K
H_2O_g	g	$\Delta_f H^\circ(298.15)$	-241.8260	kJ/mol	298.15	assessed-compilation	S8	–	CODATA Key Values table, H2O(g): dfH(298.15) = -241.826 +/- 0.040 kJ/mol, printed to the adopted digits. NIST-JANAF (H-064) prints the same value
H_2O_g	g	$S^\circ(298.15)$	188.8350	J/(mol K)	298.15	assessed-compilation	S8	–	CODATA Key Values table, H2O(g): S(298.15) = 188.835 +/- 0.010 J/(mol K); this is the printed source of the adopted 188.835. NIST-JANAF prints 188.834
H_2O_g	g	Shomate range 1	Table S1(c)	Cp in J/(mol K), T/1000	500–1700	JANAF-tabulated	S1	–	Gas Phase Heat Capacity (Shomate Equation), 500-1700 K
H_2O_g	g	Shomate range 2	Table S1(c)	Cp in J/(mol K), T/1000	1700–6000	JANAF-tabulated	S1	–	Gas Phase Heat Capacity (Shomate Equation), 1700-6000 K
MgO	s	$\Delta_f H^\circ(298.15)$	-601.6000	kJ/mol	298.15	assessed-compilation	S8	–	CODATA Key Values table, MgO(cr): dfH(298.15) = -601.60 +/- 0.30 kJ/mol. Robie & Hemingway (1995) p. 197 print the same -601.6; NIST-JANAF prints -601.24 and NBS Wagman (1982) -601.70

continued on the next page

Table S1 (continued)

Species	Phase	Parameter	Value	Units	Validity / K	Method flag	Src	Fit dev. / %	Locator within the source
MgO	s	$S^\circ(298.15)$	26.9500	J/(mol K)	298.15	assessed-compilation	S8	–	CODATA Key Values table, MgO(cr): S(298.15) = 26.95 +/- 0.15 J/(mol K), the printed source of the adopted 26.95. The NIST WebBook condensed-phase summary gives 26.85 and the NIST-JANAF table row 26.924, NBS Wagman (1982) prints 26.94 and Robie & Hemingway (1995) 26.90; all lie inside the CODATA uncertainty
MgO	s	Shomate range 1	Table S1(c)	Cp in J/(mol K), T/1000	298–3105	JANAF-tabulated	S1	–	Solid Phase Heat Capacity (Shomate Equation), 298-3105 K
CaO	s	$\Delta_f H^\circ(298.15)$	-634.9200	kJ/mol	298.15	assessed-compilation	S8	–	CODATA Key Values table, CaO(cr): dfH(298.15) = -634.92 +/- 0.90 kJ/mol, the printed source of the adopted -634.92. NIST-JANAF and NBS Wagman (1982) both print -635.09, inside the CODATA uncertainty
CaO	s	$S^\circ(298.15)$	38.2100	J/(mol K)	298.15	JANAF-tabulated	S1	–	NIST-JANAF table Ca-027, CaO(cr), 298.15 K row: S(298.15) = 38.212 J/(mol K), rounded to the adopted 38.21 (effect below 0.02 K on any turnover temperature). CODATA gives 38.1 +/- 0.4 and NBS Wagman (1982) 39.75. The NIST WebBook lists both the CODATA 38.1 +/- 0.4 and a Chase-1998 condensed-phase summary 38.19; the adopted 38.21 is the JANAF table row value 38.212
CaO	s	Shomate range 1	Table S1(c)	Cp in J/(mol K), T/1000	298–3200	JANAF-tabulated	S1	–	Solid Phase Heat Capacity (Shomate Equation), 298-3200 K
$Ca(OH)_2$	s	$\Delta_f H^\circ(298.15)$	-986.0900	kJ/mol	298.15	JANAF-tabulated	S1	–	NIST-JANAF portlandite condensed-phase entry, dfH(solid) = -986.09 kJ/mol.
$Ca(OH)_2$	s	$S^\circ(298.15)$	83.3600	J/(mol K)	298.15	JANAF-tabulated	S1	–	NIST WebBook condensed-phase summary for portlandite (Chase 1998), S(solid) = 83.36 J/(mol K).
$Ca(OH)_2$	s	Shomate range 1	Table S1(c)	Cp in J/(mol K), T/1000	298–1000	JANAF-tabulated	S1	–	Solid Phase Heat Capacity (Shomate Equation), 298-1000 K

continued on the next page

Table S1 (continued)

Species	Phase	Parameter	Value	Units	Validity / K	Method flag	Src	Fit dev. / %	Locator within the source
$CaSiO_3$	s	$\Delta_f H^\circ(298.15)$	-1634.9400	kJ/mol	298.15	assessed-compilation	S9	–	NBS Tables, Table 94:Ca (p. 2-273), CaO.SiO2 wollastonite (cr): dfH(298.15) = -1634.94 kJ/mol. See ALTERNATIVES['CaSiO3_RobieHemingway1995']
$CaSiO_3$	s	$S^\circ(298.15)$	81.9200	J/(mol K)	298.15	assessed-compilation	S9	–	NBS Tables, Table 94:Ca (p. 2-273), CaO.SiO2 wollastonite (cr): S(298.15) = 81.92 J/(mol K), the printed source of the adopted value. Robie & Hemingway (1995) p. 342 print 81.69 +/- 0.1, so the two compilations differ by 0.23 J/(mol K), beyond that stated uncertainty; the alternative is carried as a scenario
$CaSiO_3$	s	Shomate range 1	Table S1(c)	Cp in J/(mol K), T/1000	298–1398	fitted-to-published-Cp	S2	0.260	p. 61, COEFFICIENTS FOR HEAT CAPACITY EQUATIONS FOR CHAIN STRUCTURE SILICATE MINERALS, row WOLLASTONITE CaSiO3: Cp = 200.78 - 0.02589 T - 157900/T^2 - 1826.0/sqrt(T) + 7.434e-6 T^2, valid 298-1400 K
Mg_2SiO_4	s	$\Delta_f H^\circ(298.15)$	-2174.0000	kJ/mol	298.15	assessed-compilation	S9	–	NBS Tables, Table 93:Mg (p. 2-265), Mg2SiO4 forsterite (cr): dfH(298.15) = -2174.0 kJ/mol, the printed source of the adopted value. NIST-JANAF prints -2176.94 and Robie & Hemingway (1995) p. 335 prints -2173.0. See ALTERNATIVES['Mg2SiO4_Chase1998']
Mg_2SiO_4	s	$S^\circ(298.15)$	94.1100	J/(mol K)	298.15	assessed-compilation	S2	–	USGS Bull. 2131 p. 335, high-temperature table for FORSTERITE, 298.15 K row: S(298.15) = 94.11 J/(mol K), the printed source of the adopted value. NIST-JANAF table Mg-028 prints 95.140 and the NIST WebBook condensed-phase summary 94.96; NBS Wagman (1982) prints 95.14
Mg_2SiO_4	s	Shomate range 1	Table S1(c)	Cp in J/(mol K), T/1000	298–2171	JANAF-tabulated	S1	–	Solid Phase Heat Capacity (Shomate Equation), 298-2171 K

continued on the next page

Table S1 (continued)

Species	Phase	Parameter	Value	Units	Validity / K	Method flag	Src	Fit dev. / %	Locator within the source
Li_4SiO_4	s	$\Delta_f H^\circ(298.15)$	-2326.2000	kJ/mol	298.15	unlocated	S10	–	No printed source was located in NIST-JANAF, Robie and Hemingway, the NBS Tables or CODATA. This value is a provisional assumption for R6 or R7, excluded from the primary aggregate; published reaction-level alternatives are evaluated in Section S7.3.
Li_4SiO_4	s	$S^\circ(298.15)$	121.7400	J/(mol K)	298.15	unlocated	S10	–	No printed source was located in NIST-JANAF, Robie and Hemingway, the NBS Tables or CODATA. This value is a provisional assumption for R6 or R7, excluded from the primary aggregate; published reaction-level alternatives are evaluated in Section S7.3.
Li_4SiO_4	s	Shomate range 1	Table S1(c)	Cp in J/(mol K), T/1000	298–1300	fitted-to-published-Cp	S4	0.000	smoothed enthalpy equation, H(T)-H(298) = -17156 + 73.694 T + 0.103210 T^2 - 4163115/T J/mol, valid 298-1300 K (measurements 363-1300 K, isothermal drop calorimetry)
Li_2ZrO_3	s	$\Delta_f H^\circ(298.15)$	-1760.2000	kJ/mol	298.15	assessed-compilation	S9	–	NBS Tables, Table 98:Li (p. 2-298), Li2ZrO3 (cr): dfH(298.15) = -1760.2 kJ/mol. Li2ZrO3 has no NIST-JANAF table and no Robie & Hemingway (1995) entry
Li_2ZrO_3	s	$S^\circ(298.15)$	91.9800	J/(mol K)	298.15	unlocated	S10	–	No printed source was located in NIST-JANAF, Robie and Hemingway, the NBS Tables or CODATA. This value is a provisional assumption for R6 or R7, excluded from the primary aggregate; published reaction-level alternatives are evaluated in Section S7.3.
Li_2ZrO_3	s	Shomate range 1	Table S1(c)	Cp in J/(mol K), T/1000	298–1400	fitted-to-published-Cp	S5	0.000	smoothed enthalpy equation, H(T)-H(298) = -46453 + 134.857 T + 0.00740 T^2 + 1671251/T J/mol, valid 298-1400 K (measurements 368-1414 K, isoperibol drop calorimetry, Setaram HTC 1800)

continued on the next page

Table S1 (continued)

Species	Phase	Parameter	Value	Units	Validity / K	Method flag	Src	Fit dev. / %	Locator within the source
Na_2ZrO_3	s	$\Delta_f H^\circ(298.15)$	-1686.3800	kJ/mol	298.15	calorimetry	S6	–	hydrofluoric-acid solution calorimetry; the published abstract prints dfH(298.15) = -(1686.3 +/- 2.0) kJ/mol and locates the tabulation in Table 6. The article is not open access, so the table locator has not been checked against the full text in this work
Na_2ZrO_3	s	$S^\circ(298.15)$	125.4000	J/(mol K)	298.15	calorimetry	S6	–	adiabatic calorimetry 5-305 K, tabulated in Table 3 as S(298.15) - S(0) = 125.497 J/(mol K). The article is not open access, so the table locator has not been checked against the full text in this work
Na_2ZrO_3	s	Shomate range 1	Table S1(c)	Cp in J/(mol K), T/1000	298–1170	fitted-to-published-Cp	S6	0.211	Table 3, 'Molar thermodynamic functions of Na2ZrO3', Cp,m column, 25 smoothed points from 298.15 to 1170 K (adiabatic calorimetry 5-305 K joined to d.s.c. 438-1168 K)
$MgCO_3$	s	$\Delta_f H^\circ(298.15)$	-1111.6900	kJ/mol	298.15	JANAF-tabulated	S1	–	MgCO3 condensed phase, dfH(solid) = -1111.69 kJ/mol. Corroborated to 0.01 kJ/mol by the independent reversed phase-equilibrium determination of Koziol & Newton (1995), -1111.68 +/- 0.47 kJ/mol, and to 0.1 kJ/mol in dfG298 by the magnesite solubility product of Benezeth et al. (2011) combined with CODATA ion values. Remaining modern spread -1111.7 to -1114.5 kJ/mol; see ALTERNATIVES['MgCO3_RobieHemingway1995'] for the sensitivity case
$MgCO_3$	s	$S^\circ(298.15)$	65.0900	J/(mol K)	298.15	calorimetry	S7	–	adiabatic calorimetry on magnesite, 13-380 K; S(298.15) = 65.09 +/- 0.1 J/(mol K), as tabulated by Robie & Hemingway (1995) USGS Bull. 2131 p. 271 (the 298.15 K summary on p. 25 prints the same value rounded to 65.1) and adopted by Koziol & Newton (1995) Table 5.
$MgCO_3$	s	Shomate range 1	Table S1(c)	Cp in J/(mol K), T/1000	298–1000	JANAF-tabulated	S1	–	Solid Phase Heat Capacity (Shomate Equation), 298-1000 K

continued on the next page

Table S1 (continued)

Species	Phase	Parameter	Value	Units	Validity / K	Method flag	Src	Fit dev. / %	Locator within the source
$CaCO_3$	s	$\Delta_f H^\circ(298.15)$	-1206.9000	kJ/mol	298.15	assessed-compilation	S9	–	NBS Tables, Table 94:Ca (p. 2-272), CaCO3 calcite (cr): dfH(298.15) = -1206.92 kJ/mol, rounded to the adopted -1206.9. This is the printed source of the adopted value; Robie & Hemingway (1995) p. 25 print -1207.4 +/- 1.3, which is carried as ALTERNATIVES['CaCO3_RobieHemingway1995']
$CaCO_3$	s	$S^\circ(298.15)$	91.7100	J/(mol K)	298.15	assessed-compilation	S2	–	USGS Bull. 2131 p. 268, high-temperature table for CALCITE, 298.15 K row: S(298.15) = 91.71 J/(mol K); the 298.15 K summary on p. 25 prints the same value rounded to 91.7. NBS Wagman (1982) prints 92.9
$CaCO_3$	s	Shomate range 1	Table S1(c)	Cp in J/(mol K), T/1000	298–1200	fitted-to-published-Cp	S2	0.000	p. 55, COEFFICIENTS FOR HEAT CAPACITY EQUATIONS FOR CARBONATE AND NITRATE MINERALS, row CALCITE CaCO3: Cp = 99.72 + 0.02692 T - 2158000/T^2, valid 298-1200 K
SiO_2	s	$\Delta_f H^\circ(298.15)$	-910.7000	kJ/mol	298.15	assessed-compilation	S8	–	CODATA Key Values table, SiO2(cr, alpha-quartz): dfH(298.15) = -910.7 +/- 1.0 kJ/mol, the printed source of the adopted -910.7. NIST-JANAF prints -910.86 (well inside the CODATA uncertainty)
SiO_2	s	$S^\circ(298.15)$	41.4600	J/(mol K)	298.15	assessed-compilation	S8	–	CODATA Key Values table, SiO2(cr, alpha-quartz): S(298.15) = 41.46 +/- 0.20 J/(mol K). NIST-JANAF prints 41.460 from the same assessment, so the two agree exactly at the adopted precision
SiO_2	s	Shomate range 1	Table S1(c)	Cp in J/(mol K), T/1000	298–847	JANAF-tabulated	S1	–	Solid Phase Heat Capacity (Shomate Equation), 298-847 K
SiO_2	s	Shomate range 2	Table S1(c)	Cp in J/(mol K), T/1000	847–1996	JANAF-tabulated	S1	–	Solid Phase Heat Capacity (Shomate Equation), 847-1996 K
Li_2CO_3	s	$\Delta_f H^\circ(298.15)$	-1215.9000	kJ/mol	298.15	assessed-compilation	S9	–	NBS Tables, Table 98:Li (p. 2-296), Li2CO3 (cr): dfH(298.15) = -1215.9 kJ/mol, the printed source of the adopted value. NIST-JANAF prints -1216.04

continued on the next page

Table S1 (continued)

Species	Phase	Parameter	Value	Units	Validity / K	Method flag	Src	Fit dev. / %	Locator within the source
Li_2CO_3	s	$S^\circ(298.15)$	90.3700	J/(mol K)	298.15	assessed-compilation	S9	–	NBS Tables, Table 98:Li (p. 2-296), Li2CO3 (cr): S(298.15) = 90.37 J/(mol K), the printed source of the adopted value. NIST-JANAF prints 90.171 (WebBook summary 90.31). See ALTERNATIVES['Li2CO3_Chase1998']
Li_2CO_3	s	Shomate range 1	Table S1(c)	Cp in J/(mol K), T/1000	298–683	JANAF-tabulated	S1	–	Solid Phase Heat Capacity (Shomate Equation), 298-683 K
Li_2CO_3	s	Shomate range 2	Table S1(c)	Cp in J/(mol K), T/1000	683–993	JANAF-tabulated	S1	–	Solid Phase Heat Capacity (Shomate Equation), 683-993 K
Li_2CO_3	s	Shomate range 3	Table S1(c)	Cp in J/(mol K), T/1000	993–1500	JANAF-tabulated	S1	–	Liquid Phase Heat Capacity (Shomate Equation), 993-2000 K
Na_2CO_3	s	$\Delta_f H^\circ(298.15)$	-1130.7000	kJ/mol	298.15	assessed-compilation	S9	–	NBS Tables, Table 99:Na (p. 2-313), Na2CO3 (cr): dfH(298.15) = -1130.68 kJ/mol, rounded to the adopted -1130.7. NIST-JANAF prints -1130.77
Na_2CO_3	s	$S^\circ(298.15)$	134.9800	J/(mol K)	298.15	assessed-compilation	S9	–	NBS Tables, Table 99:Na (p. 2-313), Na2CO3 (cr): S(298.15) = 134.98 J/(mol K). NIST-JANAF prints 138.79, a 3.81 J/(mol K) disagreement worth about 30 K on R8; the NBS entry itself carries the note that its G-H-S constraint has been relaxed. See ALTERNATIVES['Na2CO3_Chase1998']
Na_2CO_3	s	Shomate range 1	Table S1(c)	Cp in J/(mol K), T/1000	298–723	JANAF-tabulated	S1	–	Solid Phase Heat Capacity (Shomate Equation), 298-723 K
Na_2CO_3	s	Shomate range 2	Table S1(c)	Cp in J/(mol K), T/1000	723–1123	JANAF-tabulated	S1	–	Solid Phase Heat Capacity (Shomate Equation), 723-1123 K
Na_2CO_3	s	Shomate range 3	Table S1(c)	Cp in J/(mol K), T/1000	1123–1500	JANAF-tabulated	S1	–	Liquid Phase Heat Capacity (Shomate Equation), 1123-2500 K
Li_2SiO_3	s	$\Delta_f H^\circ(298.15)$	-1648.1000	kJ/mol	298.15	assessed-compilation	S9	–	NBS Tables, Table 98:Li (p. 2-296), Li2SiO3 (cr): dfH(298.15) = -1648.1 kJ/mol, the printed source of the adopted value. NIST-JANAF table Li-020 prints -1649.500

continued on the next page

Table S1 (continued)

Species	Phase	Parameter	Value	Units	Validity / K	Method flag	Src	Fit dev. / %	Locator within the source
Li_2SiO_3	s	$S^\circ(298.15)$	79.8300	J/(mol K)	298.15	assessed-compilation	S9	–	NBS Tables, Table 98:Li (p. 2-296), Li2SiO3 (cr): S(298.15) = 79.83 J/(mol K). NIST-JANAF table Li-020 prints 80.295 (WebBook summary 80.24). See ALTERNATIVES['Li2SiO3_Chase1998_anchors']
Li_2SiO_3	s	Shomate range 1	Table S1(c)	Cp in J/(mol K), T/1000	298–1474	JANAF-tabulated	S1	–	Solid Phase Heat Capacity (Shomate Equation), 298-1474 K
ZrO_2	s	$\Delta_f H^\circ(298.15)$	-1100.6000	kJ/mol	298.15	assessed-compilation	S2	–	USGS Bull. 2131 p. 228, high-temperature table for BADDELEYITE (ZrO2, monoclinic), 298.15 K row: dfH(298.15) = -1100.6 kJ/mol. NBS Wagman (1982) prints -1100.56; NIST-JANAF prints -1097.46
ZrO_2	s	$S^\circ(298.15)$	50.4000	J/(mol K)	298.15	assessed-compilation	S2	–	USGS Bull. 2131 p. 228, BADDELEYITE 298.15 K row: S(298.15) = 50.40 J/(mol K). NBS Wagman (1982) prints 50.38 and the NIST WebBook condensed-phase summary 50.34; the JANAF pair is carried as a scenario, see ALTERNATIVES['ZrO2_Chase1998_anchors']
ZrO_2	s	Shomate range 1	Table S1(c)	Cp in J/(mol K), T/1000	298–1478	JANAF-tabulated	S1	–	Solid Phase Heat Capacity (Shomate Equation), 298-1478 K
ZrO_2	s	Shomate range 2	Table S1(c)	Cp in J/(mol K), T/1000	1478–2950	JANAF-tabulated	S1	–	Solid Phase Heat Capacity (Shomate Equation), 1478-2950 K
$Mg(OH)_2$	s	$\Delta_f H^\circ(298.15)$	-924.5000	kJ/mol	298.15	assessed-compilation	S2	–	high-temperature table for BRUCITE (brucite, Mg(OH)2), USGS Bull. 2131 p. 198; 298.15 K row. Source reference codes given by the bulletin for S / dfH,dfG / Cp: 419 / 419 / 228, dfH298 = -924.5 kJ/mol
$Mg(OH)_2$	s	$S^\circ(298.15)$	63.2000	J/(mol K)	298.15	assessed-compilation	S2	–	high-temperature table for BRUCITE (brucite, Mg(OH)2), USGS Bull. 2131 p. 198; 298.15 K row. Source reference codes given by the bulletin for S / dfH,dfG / Cp: 419 / 419 / 228, S298 = 63.20 J/(mol K)

continued on the next page

Table S1 (continued)

Species	Phase	Parameter	Value	Units	Validity / K	Method flag	Src	Fit dev. / %	Locator within the source
$Mg(OH)_2$	s	Shomate range 1	Table S1(c)	Cp in J/(mol K), T/1000	298–900	fitted-to-published-Cp	S2	0.001	high-temperature table for BRUCITE (brucite, Mg(OH)2), USGS Bull. 2131 p. 198; 298.15 K row. Source reference codes given by the bulletin for S / dfH,dfG / Cp: 419 / 419 / 228, Cp column
$MgSiO_3$	s	$\Delta_f H^\circ(298.15)$	-1545.6000	kJ/mol	298.15	assessed-compilation	S2	–	high-temperature table for ENSTATITE (enstatite, MgSiO3), USGS Bull. 2131 p. 352; 298.15 K row. Source reference codes given by the bulletin for S / dfH,dfG / Cp: 259 / 37 / 257, dfH298 = -1545.6 kJ/mol
$MgSiO_3$	s	$S^\circ(298.15)$	66.2700	J/(mol K)	298.15	assessed-compilation	S2	–	high-temperature table for ENSTATITE (enstatite, MgSiO3), USGS Bull. 2131 p. 352; 298.15 K row. Source reference codes given by the bulletin for S / dfH,dfG / Cp: 259 / 37 / 257, S298 = 66.27 J/(mol K)
$MgSiO_3$	s	Shomate range 1	Table S1(c)	Cp in J/(mol K), T/1000	298–1000	fitted-to-published-Cp	S2	0.066	high-temperature table for ENSTATITE (enstatite, MgSiO3), USGS Bull. 2131 p. 352; 298.15 K row. Source reference codes given by the bulletin for S / dfH,dfG / Cp: 259 / 37 / 257, Cp column
Ca_2SiO_4	s	$\Delta_f H^\circ(298.15)$	-2306.7000	kJ/mol	298.15	assessed-compilation	S2	–	high-temperature table for LARNITE (larnite (beta-Ca2SiO4), Ca2SiO4), USGS Bull. 2131 p. 324; 298.15 K row. Source reference codes given by the bulletin for S / dfH,dfG / Cp: 225 / 149 / 84, dfH298 = -2306.7 kJ/mol
Ca_2SiO_4	s	$S^\circ(298.15)$	127.6000	J/(mol K)	298.15	assessed-compilation	S2	–	high-temperature table for LARNITE (larnite (beta-Ca2SiO4), Ca2SiO4), USGS Bull. 2131 p. 324; 298.15 K row. Source reference codes given by the bulletin for S / dfH,dfG / Cp: 225 / 149 / 84, S298 = 127.60 J/(mol K)
Ca_2SiO_4	s	Shomate range 1	Table S1(c)	Cp in J/(mol K), T/1000	298–970	fitted-to-published-Cp	S2	0.209	high-temperature table for LARNITE (larnite (beta-Ca2SiO4), Ca2SiO4), USGS Bull. 2131 p. 324; 298.15 K row. Source reference codes given by the bulletin for S / dfH,dfG / Cp: 225 / 149 / 84, Cp column

continued on the next page

Table S1 (continued)

Species	Phase	Parameter	Value	Units	Validity / K	Method flag	Src	Fit dev. / %	Locator within the source
$CaMgSi_2O_6$	s	$\Delta_f H^\circ(298.15)$	-3201.5000	kJ/mol	298.15	assessed-compilation	S2	–	high-temperature table for DIOPSIDE (diopside, CaMgSi2O6), USGS Bull. 2131 p. 347; 298.15 K row. Source reference codes given by the bulletin for S / dfH,dfG / Cp: 259 / 165 / 257, dfH298 = -3201.5 kJ/mol
$CaMgSi_2O_6$	s	$S^\circ(298.15)$	142.7000	J/(mol K)	298.15	assessed-compilation	S2	–	high-temperature table for DIOPSIDE (diopside, CaMgSi2O6), USGS Bull. 2131 p. 347; 298.15 K row. Source reference codes given by the bulletin for S / dfH,dfG / Cp: 259 / 165 / 257, S298 = 142.70 J/(mol K)
$CaMgSi_2O_6$	s	Shomate range 1	Table S1(c)	Cp in J/(mol K), T/1000	298–1600	fitted-to-published-Cp	S2	0.257	high-temperature table for DIOPSIDE (diopside, CaMgSi2O6), USGS Bull. 2131 p. 347; 298.15 K row. Source reference codes given by the bulletin for S / dfH,dfG / Cp: 259 / 165 / 257, Cp column
$CaAl_2Si_2O_8$	s	$\Delta_f H^\circ(298.15)$	-4234.0000	kJ/mol	298.15	assessed-compilation	S2	–	high-temperature table for ANORTHITE (anorthite, CaAl2Si2O8), USGS Bull. 2131 p. 362; 298.15 K row. Source reference codes given by the bulletin for S / dfH,dfG / Cp: 366 / 150 / 446, dfH298 = -4234.0 kJ/mol
$CaAl_2Si_2O_8$	s	$S^\circ(298.15)$	199.3000	J/(mol K)	298.15	assessed-compilation	S2	–	high-temperature table for ANORTHITE (anorthite, CaAl2Si2O8), USGS Bull. 2131 p. 362; 298.15 K row. Source reference codes given by the bulletin for S / dfH,dfG / Cp: 366 / 150 / 446, S298 = 199.30 J/(mol K)
$CaAl_2Si_2O_8$	s	Shomate range 1	Table S1(c)	Cp in J/(mol K), T/1000	298–1600	fitted-to-published-Cp	S2	0.195	high-temperature table for ANORTHITE (anorthite, CaAl2Si2O8), USGS Bull. 2131 p. 362; 298.15 K row. Source reference codes given by the bulletin for S / dfH,dfG / Cp: 366 / 150 / 446, Cp column

continued on the next page

Table S1 (continued)

Species	Phase	Parameter	Value	Units	Validity / K	Method flag	Src	Fit dev. / %	Locator within the source
Al_2SiO_5	s	$\Delta_f H^\circ(298.15)$	-2593.8000	kJ/mol	298.15	assessed-compilation	S2	–	high-temperature table for KYANITE (kyanite, Al2SiO5), USGS Bull. 2131 p. 304; 298.15 K row. Source reference codes given by the bulletin for S / dfH,dfG / Cp: 355 / 177 / 177, dfH298 = -2593.8 kJ/mol
Al_2SiO_5	s	$S^\circ(298.15)$	82.8000	J/(mol K)	298.15	assessed-compilation	S2	–	high-temperature table for KYANITE (kyanite, Al2SiO5), USGS Bull. 2131 p. 304; 298.15 K row. Source reference codes given by the bulletin for S / dfH,dfG / Cp: 355 / 177 / 177, S298 = 82.80 J/(mol K)
Al_2SiO_5	s	Shomate range 1	Table S1(c)	Cp in J/(mol K), T/1000	298–1600	fitted-to-published-Cp	S2	0.162	high-temperature table for KYANITE (kyanite, Al2SiO5), USGS Bull. 2131 p. 304; 298.15 K row. Source reference codes given by the bulletin for S / dfH,dfG / Cp: 355 / 177 / 177, Cp column
$CaMg(CO_3)_2$	s	$\Delta_f H^\circ(298.15)$	-2324.5000	kJ/mol	298.15	assessed-compilation	S2	–	high-temperature table for DOLOMITE (dolomite, CaMg(CO3)2), USGS Bull. 2131 p. 269; 298.15 K row. Source reference codes given by the bulletin for S / dfH,dfG / Cp: 396 / 175 / 257, dfH298 = -2324.5 kJ/mol
$CaMg(CO_3)_2$	s	$S^\circ(298.15)$	155.1800	J/(mol K)	298.15	assessed-compilation	S2	–	high-temperature table for DOLOMITE (dolomite, CaMg(CO3)2), USGS Bull. 2131 p. 269; 298.15 K row. Source reference codes given by the bulletin for S / dfH,dfG / Cp: 396 / 175 / 257, S298 = 155.18 J/(mol K)
$CaMg(CO_3)_2$	s	Shomate range 1	Table S1(c)	Cp in J/(mol K), T/1000	298–1100	fitted-to-published-Cp	S2	0.071	high-temperature table for DOLOMITE (dolomite, CaMg(CO3)2), USGS Bull. 2131 p. 269; 298.15 K row. Source reference codes given by the bulletin for S / dfH,dfG / Cp: 396 / 175 / 257, Cp column

continued on the next page

Table S1 (continued)

Species	Phase	Parameter	Value	Units	Validity / K	Method flag	Src	Fit dev. / %	Locator within the source
$Mg_3Si_4O_{10}(OH)_2$	s	$\Delta_f H^\circ(298.15)$	-5900.0000	kJ/mol	298.15	assessed-compilation	S2	–	high-temperature table for TALC (talc, Mg3Si4O10(OH)2), USGS Bull. 2131 p. 393; 298.15 K row. Source reference codes given by the bulletin for S / dfH,dfG / Cp: 370 / 162 / 257, dfH298 = -5900.0 kJ/mol
$Mg_3Si_4O_{10}(OH)_2$	s	$S^\circ(298.15)$	260.8000	J/(mol K)	298.15	assessed-compilation	S2	–	high-temperature table for TALC (talc, Mg3Si4O10(OH)2), USGS Bull. 2131 p. 393; 298.15 K row. Source reference codes given by the bulletin for S / dfH,dfG / Cp: 370 / 162 / 257, S298 = 260.80 J/(mol K)
$Mg_3Si_4O_{10}(OH)_2$	s	Shomate range 1	Table S1(c)	Cp in J/(mol K), T/1000	298–800	fitted-to-published-Cp	S2	0.652	high-temperature table for TALC (talc, Mg3Si4O10(OH)2), USGS Bull. 2131 p. 393; 298.15 K row. Source reference codes given by the bulletin for S / dfH,dfG / Cp: 370 / 162 / 257, Cp column
$Ca_3Al_2Si_3O_{12}$	s	$\Delta_f H^\circ(298.15)$	-6640.0000	kJ/mol	298.15	assessed-compilation	S2	–	high-temperature table for GROSSULAR (grossular, Ca3Al2Si3O12), USGS Bull. 2131 p. 316; 298.15 K row. Source reference codes given by the bulletin for S / dfH,dfG / Cp: 157 / 68 / 258, dfH298 = -6640.0 kJ/mol
$Ca_3Al_2Si_3O_{12}$	s	$S^\circ(298.15)$	260.1200	J/(mol K)	298.15	assessed-compilation	S2	–	high-temperature table for GROSSULAR (grossular, Ca3Al2Si3O12), USGS Bull. 2131 p. 316; 298.15 K row. Source reference codes given by the bulletin for S / dfH,dfG / Cp: 157 / 68 / 258, S298 = 260.12 J/(mol K)
$Ca_3Al_2Si_3O_{12}$	s	Shomate range 1	Table S1(c)	Cp in J/(mol K), T/1000	298–1200	fitted-to-published-Cp	S2	0.156	high-temperature table for GROSSULAR (grossular, Ca3Al2Si3O12), USGS Bull. 2131 p. 316; 298.15 K row. Source reference codes given by the bulletin for S / dfH,dfG / Cp: 157 / 68 / 258, Cp column

continued on the next page

Table S1 (continued)

Species	Phase	Parameter	Value	Units	Validity / K	Method flag	Src	Fit dev. / %	Locator within the source
$Mg_3Al_2Si_3O_{12}$	s	$\Delta_f H^\circ(298.15)$	-6285.0000	kJ/mol	298.15	assessed-compilation	S2	–	high-temperature table for PYROPE (pyrope, Mg3Al2Si3O12), USGS Bull. 2131 p. 336; 298.15 K row. Source reference codes given by the bulletin for S / dfH,dfG / Cp: 157 / 188 / 453, dfH298 = -6285.0 kJ/mol
$Mg_3Al_2Si_3O_{12}$	s	$S^\circ(298.15)$	266.2700	J/(mol K)	298.15	assessed-compilation	S2	–	high-temperature table for PYROPE (pyrope, Mg3Al2Si3O12), USGS Bull. 2131 p. 336; 298.15 K row. Source reference codes given by the bulletin for S / dfH,dfG / Cp: 157 / 188 / 453, S298 = 266.27 J/(mol K)
$Mg_3Al_2Si_3O_{12}$	s	Shomate range 1	Table S1(c)	Cp in J/(mol K), T/1000	298–1500	fitted-to-published-Cp	S2	0.192	high-temperature table for PYROPE (pyrope, Mg3Al2Si3O12), USGS Bull. 2131 p. 336; 298.15 K row. Source reference codes given by the bulletin for S / dfH,dfG / Cp: 157 / 188 / 453, Cp column
$Al_2Si_2O_5(OH)_4$	s	$\Delta_f H^\circ(298.15)$	-4119.0000	kJ/mol	298.15	assessed-compilation	S2	–	high-temperature table for KAOLINITE (kaolinite, Al2Si2O5(OH)4), USGS Bull. 2131 p. 382; 298.15 K row. Source reference codes given by the bulletin for S / dfH,dfG / Cp: 358 / 179 / 179, dfH298 = -4119.0 kJ/mol
$Al_2Si_2O_5(OH)_4$	s	$S^\circ(298.15)$	200.4000	J/(mol K)	298.15	assessed-compilation	S2	–	high-temperature table for KAOLINITE (kaolinite, Al2Si2O5(OH)4), USGS Bull. 2131 p. 382; 298.15 K row. Source reference codes given by the bulletin for S / dfH,dfG / Cp: 358 / 179 / 179, S298 = 200.40 J/(mol K)
$Al_2Si_2O_5(OH)_4$	s	Shomate range 1	Table S1(c)	Cp in J/(mol K), T/1000	298–800	fitted-to-published-Cp	S2	0.209	high-temperature table for KAOLINITE (kaolinite, Al2Si2O5(OH)4), USGS Bull. 2131 p. 382; 298.15 K row. Source reference codes given by the bulletin for S / dfH,dfG / Cp: 358 / 179 / 179, Cp column

continued on the next page

Table S1 (continued)

Species	Phase	Parameter	Value	Units	Validity / K	Method flag	Src	Fit dev. / %	Locator within the source
$Ca_2MgSi_2O_7$	s	$\Delta_f H^\circ(298.15)$	-3864.8000	kJ/mol	298.15	assessed-compilation	S2	–	high-temperature table for AKERMANITE (akermanite, Ca2MgSi2O7), USGS Bull. 2131, 'THERMODYNAMIC PROPERTIES AT HIGH TEMPERATURE OF INDIVIDUAL PHASES'; 298.15 K row. Source reference codes given by the bulletin for S / dfH,dfG / Cp: 165 / 165 / 165, dfH298 = -3864.8 kJ/mol
$Ca_2MgSi_2O_7$	s	$S^\circ(298.15)$	212.5000	J/(mol K)	298.15	assessed-compilation	S2	–	high-temperature table for AKERMANITE (akermanite, Ca2MgSi2O7), USGS Bull. 2131, 'THERMODYNAMIC PROPERTIES AT HIGH TEMPERATURE OF INDIVIDUAL PHASES'; 298.15 K row. Source reference codes given by the bulletin for S / dfH,dfG / Cp: 165 / 165 / 165, S298 = 212.50 J/(mol K)
$Ca_2MgSi_2O_7$	s	Shomate range 1	Table S1(c)	Cp in J/(mol K), T/1000	298–1600	fitted-to-published-Cp	S2	0.126	high-temperature table for AKERMANITE (akermanite, Ca2MgSi2O7), USGS Bull. 2131, 'THERMODYNAMIC PROPERTIES AT HIGH TEMPERATURE OF INDIVIDUAL PHASES'; 298.15 K row. Source reference codes given by the bulletin for S / dfH,dfG / Cp: 165 / 165 / 165, Cp column
$Ca_3Mg(SiO_4)_2$	s	$\Delta_f H^\circ(298.15)$	-4536.2000	kJ/mol	298.15	assessed-compilation	S2	–	high-temperature table for MERWINITE (merwinite, Ca3Mg(SiO4)2), USGS Bull. 2131, 'THERMODYNAMIC PROPERTIES AT HIGH TEMPERATURE OF INDIVIDUAL PHASES'; 298.15 K row. Source reference codes given by the bulletin for S / dfH,dfG / Cp: 427 / 49 / 323, dfH298 = -4536.2 kJ/mol

continued on the next page

Table S1 (continued)

Species	Phase	Parameter	Value	Units	Validity / K	Method flag	Src	Fit dev. / %	Locator within the source
$Ca_3Mg(SiO_4)_2$	s	$S^\circ(298.15)$	253.1000	J/(mol K)	298.15	assessed-compilation	S2	–	high-temperature table for MERWINITE (merwinite, Ca3Mg(SiO4)2), USGS Bull. 2131, 'THERMODYNAMIC PROPERTIES AT HIGH TEMPERATURE OF INDIVIDUAL PHASES'; 298.15 K row. Source reference codes given by the bulletin for S / dfH,dfG / Cp: 427 / 49 / 323, S298 = 253.10 J/(mol K)
$Ca_3Mg(SiO_4)_2$	s	Shomate range 1	Table S1(c)	Cp in J/(mol K), T/1000	298–1600	fitted-to-published-Cp	S2	0.214	high-temperature table for MERWINITE (merwinite, Ca3Mg(SiO4)2), USGS Bull. 2131, 'THERMODYNAMIC PROPERTIES AT HIGH TEMPERATURE OF INDIVIDUAL PHASES'; 298.15 K row. Source reference codes given by the bulletin for S / dfH,dfG / Cp: 427 / 49 / 323, Cp column
$Ca_3Si_2O_7$	s	$\Delta_f H^\circ(298.15)$	-3949.0000	kJ/mol	298.15	assessed-compilation	S2	–	high-temperature table for RANKINITE (rankinite, Ca3Si2O7), USGS Bull. 2131 p. 327; 298.15 K row. Source reference codes given by the bulletin for S / dfH,dfG / Cp: 149 / 410 / 149, dfH298 = -3949.0 kJ/mol
$Ca_3Si_2O_7$	s	$S^\circ(298.15)$	210.6000	J/(mol K)	298.15	assessed-compilation	S2	–	high-temperature table for RANKINITE (rankinite, Ca3Si2O7), USGS Bull. 2131 p. 327; 298.15 K row. Source reference codes given by the bulletin for S / dfH,dfG / Cp: 149 / 410 / 149, S298 = 210.60 J/(mol K)
$Ca_3Si_2O_7$	s	Shomate range 1	Table S1(c)	Cp in J/(mol K), T/1000	298–1400	fitted-to-published-Cp	S2	0.112	high-temperature table for RANKINITE (rankinite, Ca3Si2O7), USGS Bull. 2131 p. 327; 298.15 K row. Source reference codes given by the bulletin for S / dfH,dfG / Cp: 149 / 410 / 149, Cp column

continued on the next page

Table S1 (continued)

Species	Phase	Parameter	Value	Units	Validity / K	Method flag	Src	Fit dev. / %	Locator within the source
$Mg_3Si_2O_5(OH)_4$	s	$\Delta_f H^\circ(298.15)$	-4360.0000	kJ/mol	298.15	assessed-compilation	S2	–	298.15 K summary table for SHEET STRUCTURE SILICATE MINERALS, row CHRYSOTILE Mg3Si2O5(OH)4, USGS Bull. 2131 p. 39 (the phase is also listed at p. 394); source reference codes for S / dfH,dfG / Cp: 226 / 171 / 226, dfH298 = -4360.0 +/- 3.0 kJ/mol
$Mg_3Si_2O_5(OH)_4$	s	$S^\circ(298.15)$	221.3000	J/(mol K)	298.15	assessed-compilation	S2	–	298.15 K summary table for SHEET STRUCTURE SILICATE MINERALS, row CHRYSOTILE Mg3Si2O5(OH)4, USGS Bull. 2131 p. 39 (the phase is also listed at p. 394); source reference codes for S / dfH,dfG / Cp: 226 / 171 / 226, S298 = 221.3 +/- 0.8 J/(mol K)
$Mg_3Si_2O_5(OH)_4$	s	Shomate range 1	Table S1(c)	Cp in J/(mol K), T/1000	298–900	fitted-to-published-Cp	S2	0.111	298.15 K summary table for SHEET STRUCTURE SILICATE MINERALS, row CHRYSOTILE Mg3Si2O5(OH)4, USGS Bull. 2131 p. 39 (the phase is also listed at p. 394); source reference codes for S / dfH,dfG / Cp: 226 / 171 / 226, Cp equation p. 66

Table S1(b) Source key for Table S1. Source identifiers are retained as stable keys; S3 is unused.

Key	Source
S1	Chase, M. W., Jr., NIST-JANAF Thermochemical Tables, 4th ed., J. Phys. Chem. Ref. Data, Monograph 9, 1998, 1-1951. ISBN 1-56396-831-2
S2	Robie, R. A. and Hemingway, B. S., Thermodynamic properties of minerals and related substances at 298.15 K and 1 bar (10^5 pascals) pressure and at higher temperatures, U.S. Geological Survey Bulletin 2131, 1995, 461 pp. doi:10.3133/b2131
S4	Kleykamp, H., Enthalpy, heat capacity, second-order transitions and enthalpy of fusion of Li4SiO4 by high-temperature calorimetry, Thermochim. Acta, 1996, 287, 191-201. doi:10.1016/S0040-6031(96)02996-6
S5	Kleykamp, H., Enthalpies and heat capacities of Li2SiO3 and Li2ZrO3 between 298 and 1400 K by drop calorimetry, Thermochim. Acta, 1994, 237, 1-12. doi:10.1016/0040-6031(94)85178-6
S6	Beyer, R. P., Bennington, K. O. and Brown, R. R., Sodium zirconium oxide: molar enthalpy of formation at 298.15 K and molar heat capacity from 5 to 1168 K, J. Chem. Thermodyn., 1985, 17, 11-17. doi:10.1016/0021-9614(85)90026-6
S7	Hemingway, B. S., Robie, R. A., Fisher, J. R. and Wilson, W. H., The heat capacities of gibbsite, Al(OH)3, between 13 and 480 K and magnesite, MgCO3, between 13 and 380 K and their standard entropies at 298.15 K, and the heat capacities of Calorimetry Conference benzoic acid between 12 and 316 K, J. Res. U.S. Geol. Surv., 1977, 5, 797-806. OCLC 3073949 (USGS Journal of Research, v. 5, no. 6)
S8	Cox, J. D., Wagman, D. D. and Medvedev, V. A., CODATA Key Values for Thermodynamics, Hemisphere Publishing Corporation, New York, 1989, 271 pp. ISBN 0-89116-758-7

Key	Source
S9	Wagman, D. D., Evans, W. H., Parker, V. B., Schumm, R. H., Halow, I., Bailey, S. M., Churney, K. L. and Nuttall, R. L., The NBS tables of chemical thermodynamic properties, J. Phys. Chem. Ref. Data, 1982, 11, Supplement 2. NBS, now NIST
S10	No primary or assessed compilation prints this value. It is treated as a provisional assumption and excluded from the primary aggregate. The four compilations searched were NIST-JANAF (S1), Robie and Hemingway (S2), the NBS Tables (S9) and the CODATA Key Values (S8); the reaction-level alternatives evaluated in Section S7.3 in place of a sourced anchor are Duan, J. Renew. Sustain. Energy, 2011, **3**, 013102, Table VI and note a at 300 K, doi:10.1063/1.3529427, and Duan, Pfeiffer, Li, Romero-Ibarra, Sorescu, Luebke and Halley, Phys. Chem. Chem. Phys., 2013, **15**, 13538, Table 5 at 300 K, doi:10.1039/c3cp51659h. no locator exists

Table S1(c) Full-precision calibrated Shomate coefficients used in the calculations. The temperature variable is $t = T/1000$ in the equation given in Section S1. Coefficients A–E and G are on the standard J mol^{-1} K^{-1} Shomate basis; F and H are on the kJ mol^{-1} basis.

Species	Range	Validity / K	A	B	C	D	E	F	G	H
CO_2	1	298–1200	24.997350	55.186960	-33.691370	7.948387	-0.136638	-403.604180	228.240466	-393.522000
CO_2	2	1200–6000	58.166390	2.720074	-0.492289	0.038844	-6.447293	-425.912113	263.614657	-393.522000
H_2O(g)	1	500–1700	30.092000	6.832514	6.793435	-2.534480	0.082139	-250.881127	223.396431	-241.826000
H_2O(g)	2	1700–6000	41.964260	8.622053	-1.499780	0.098119	-11.157640	-272.176898	219.782236	-241.826000
MgO	1	298–3105	47.259950	5.681621	-0.872665	0.104300	-1.053955	-619.470562	76.557729	-601.600000
CaO	1	298–3200	49.954030	4.887916	-0.352056	0.046187	-0.825097	-652.795416	92.579271	-634.920000
$Ca(OH)_2$	1	298–1000	130.825300	-82.692160	122.769000	-50.392100	-2.513146	-1030.834357	247.186811	-986.090000
$CaSiO_3$	1	298–1398	83.622570	87.487302	-61.915861	16.923430	-1.660590	-1668.816673	150.294334	-1634.940000
Mg_2SiO_4	1	298–2171	140.874900	47.152010	-12.227700	1.721771	-3.147210	-2228.548773	233.358711	-2174.000000
Li_4SiO_4	1	298–1300	73.694000	206.420000	0.000000	0.000000	4.163115	-2343.383400	172.793630	-2326.200000
Li_2ZrO_3	1	298–1400	134.857000	14.800000	0.000000	0.000000	-1.671251	-1806.670829	241.365426	-1760.200000
Na_2ZrO_3	1	298–1170	109.516619	177.835522	-182.480665	61.619621	-1.982859	-1732.096726	201.324086	-1686.380000
$MgCO_3$	1	298–1000	44.937000	149.708500	-74.182740	11.976700	-0.629261	-1133.220860	74.487264	-1111.690000
$CaCO_3$	1	298–1200	99.720000	26.920000	0.000000	0.000000	-2.158000	-1245.065991	192.222683	-1206.900000
SiO_2	1	298–847	-6.076591	251.675500	-324.796400	168.560400	0.002548	-917.529438	-27.969376	-910.700000
SiO_2	2	847–1996	58.753400	10.279250	-0.131384	0.025210	0.025601	-929.169338	105.813209	-910.700000
Li_2CO_3	1	298–683	68.332300	146.639000	-162.573000	248.026000	-0.702297	-1244.200135	130.426924	-1215.900000
Li_2CO_3	2	683–993	-6.394740	261.466000	-102.005000	42.472300	-0.168328	-1211.650160	35.829636	-1215.900000
Li_2CO_3	3	993–1500	185.435000	-0.000051	0.000030	-0.000006	-0.000003	-1251.340161	305.471549	-1215.900000
Na_2CO_3	1	298–723	175.201000	-348.058000	743.072000	-305.551000	-1.634221	-1178.908418	411.254750	-1130.700000
Na_2CO_3	2	723–1123	-1067.000000	2469.340000	-1829.060000	505.748000	100.182000	-607.052392	-1360.242266	-1130.700000
Na_2CO_3	3	1123–1500	189.535000	-0.000007	0.000002	-0.000000	-0.000003	-1182.988490	339.170644	-1130.700000
Li_2SiO_3	1	298–1474	119.837700	41.993970	-7.954956	0.878038	-2.756776	-1694.873832	197.171857	-1648.100000
ZrO_2	1	298–1478	69.200010	8.548290	-0.862921	0.246374	-1.382767	-1126.242613	123.852822	-1100.600000
ZrO_2	2	1478–2950	74.475200	0.000000	0.000000	0.000000	0.000000	-1118.460613	138.085077	-1100.600000
$Mg(OH)_2$	1	298–900	102.203249	15.108377	0.000000	0.000000	-2.616845	-964.420357	167.658572	-924.500000
$MgSiO_3$	1	298–1000	50.673272	202.563490	-204.895248	80.419286	-1.058407	-1571.610148	69.641608	-1545.600000
Ca_2SiO_4	1	298–970	143.127550	19.063954	78.741670	-57.857596	-2.286178	-2358.470035	279.275353	-2306.700000
$CaMgSi_2O_6$	1	298–1600	168.681607	173.777211	-124.425548	33.577544	-3.880020	-3271.496990	278.429481	-3201.500000
$CaAl_2Si_2O_8$	1	298–1600	229.826960	166.816825	-103.370830	31.975522	-5.332223	-4326.971667	402.010408	-4234.000000
Al_2SiO_5	1	298–1600	136.343607	121.978100	-72.225190	15.880898	-4.016503	-2652.737087	191.907808	-2593.800000
$CaMg(CO_3)_2$	1	298–1100	100.369113	328.936693	-275.717127	101.133635	-1.700983	-2372.514291	180.363777	-2324.500000
$Mg_3Si_4O_{10}(OH)_2$	1	298–800	775.436945	-1005.316315	916.121537	0.000000	-20.968570	-6164.935948	1340.275938	-5900.000000
$Ca_3Al_2Si_3O_{12}$	1	298–1200	261.245470	643.228271	-650.482506	243.943392	-6.362454	-6762.554702	375.459639	-6640.000000
$Mg_3Al_2Si_3O_{12}$	1	298–1500	314.575511	385.602313	-272.094871	70.471557	-7.254940	-6417.998020	502.653102	-6285.000000
$Al_2Si_2O_5(OH)_4$	1	298–800	255.883575	238.545325	-132.950506	0.000000	-6.395397	-4226.169955	408.874350	-4119.000000
$Ca_2MgSi_2O_7$	1	298–1600	257.254814	3.954824	57.149220	-19.937132	-4.340234	-3956.699018	495.863454	-3864.800000
$Ca_3Mg(SiO_4)_2$	1	298–1600	271.434124	163.017192	-123.891931	43.038877	-5.150259	-4640.638213	509.132385	-4536.200000
$Ca_3Si_2O_7$	1	298–1400	194.320552	229.468145	-165.938860	40.243230	-3.089712	-4026.112232	366.983916	-3949.000000
$Mg_3Si_2O_5(OH)_4$	1	298–900	114.813490	831.667664	-793.030230	264.092547	-2.269551	-4432.324318	132.429694	-4360.000000

Table S1(d) Scientifically material qualifications carried with the thermochemical inputs. Routine source-confirmation and fit-residual notes are already represented by the source locator and fit-deviation columns of Table S1 and are not repeated.

Species	Interval	Qualification
$H_2O(g)$	500–1700 K	The published Shomate range begins at 500 K. Sub-500 K values are extrapolated and explicitly flagged; the extrapolated C_p agrees with the tabulated value to 0.0041% at 300 K and 0.0033% at 400 K.
$Ca(OH)_2$	298–1000 K	No published range is available above 1000 K for portlandite, which decomposes. Values above 1000 K are extrapolations and are flagged.
Li_4SiO_4	298–1300 K	The coefficient set is an exact algebraic re-expression of the source enthalpy equation. It absorbs second-order transitions at 938 and 996 K, so the value above about 880 K is an effective transition-inclusive quantity suitable for H and S, not a lattice heat capacity.
Na_2ZrO_3	298–1170 K	The source enthalpy column contains a non-isothermal transition of about 1.0 kJ mol^{-1} between 1025 and 1110 K that is not resolved by the smoothed heat-capacity table and is therefore not represented. The R8 turnover at 1 bar, 1032.2 K, lies inside that interval; carrying the full 1.0 kJ mol^{-1} as a first-order step would move it by about 7.5 K. This is the one first-order transition inside a modeled window that the database does not carry, and it is flagged in the footnote of Table 1 of the article.
$MgCO_3$	298–1000 K	The NIST-JANAF table prints $C_p(298.15) = 76.21$ J mol^{-1} K^{-1} and the adopted Shomate block reproduces it; measured magnesite is approximately 75.5 J mol^{-1} K^{-1}, a gap of approximately 0.7 J mol^{-1} K^{-1}.
SiO_2	298–847 K	The adopted coefficients give $C_p(298.15) = 44.58$ J mol^{-1} K^{-1} for α-quartz.
SiO_2	847–1996 K	α to β quartz transition at 847 K. The source coefficient sets give $H(847\ \text{K}) = 34.196$ and 34.925 kJ mol^{-1}, so $\Delta H_{\text{trans}} = 0.7291$ kJ mol^{-1} and $\Delta S_{\text{trans}} = 0.8608$ J mol^{-1} K^{-1}, both now carried. The submitted database set this transition enthalpy to zero without a qualification.
Li_2CO_3	683–993 K	The transition enthalpy at 683 K is 2.7622 kJ mol^{-1} from the source coefficient sets.
Li_2CO_3	993–1500 K	Molten phase above 993 K; the source coefficient sets give $\Delta H_{\text{fus}} = 44.6870$ kJ mol^{-1}. A solid-only comparison basis is reported alongside the turnover temperatures.
Na_2CO_3	723–1123 K	The transition enthalpy at 723 K is 0.6776 kJ mol^{-1} from the source coefficient sets.
Na_2CO_3	1123–1500 K	Molten phase above 1123 K; the source coefficient sets give $\Delta H_{\text{fus}} = 29.6600$ kJ mol^{-1}.
ZrO_2	1478–2950 K	Monoclinic-to-tetragonal transition at 1478 K. The source coefficient sets give $H(1478\ \text{K}) = 86.263$ and 92.204 kJ mol^{-1} on the two sides, so $\Delta H_{\text{trans}} = 5.9411$ kJ mol^{-1} and $\Delta S_{\text{trans}} = 4.0197$ J mol^{-1} K^{-1}, with both increments included.
Li_2SiO_3	298–1474 K	JANAF gives $C_p(298) = 100.66$ J mol^{-1} K^{-1}; Kleykamp reports 95.4 ± 3.1 J mol^{-1} K^{-1}. The latter is retained as a named sensitivity alternative in Section S6.3.
$MgSiO_3$	298–1000 K	The fit to nine printed C_p values has a maximum deviation of 0.066%. A reversed-equilibrium check gives $\Delta_f H^\circ(298) \approx -1545.7$ kJ mol^{-1}, compared with -1545.6 kJ mol^{-1} adopted here. The older JANAF value, -1548.92 kJ mol^{-1}, is retained only as an alternative.
Ca_2SiO_4	298–970 K	Validity is capped at 970 K because bredigite is the stable Ca_2SiO_4 modification from 970 to 1710 K; the larnite table above 970 K describes a metastable phase.
Al_2SiO_5	298–1600 K	Kyanite is adopted because the three aluminosilicate reactions inherit their remaining anchors from the same bulletin page set ; the effect of the polymorph choice is evaluated separately. Andalusite is the stable polymorph at 1 bar over much of the temperature range; its assessed anchors are carried as scenario `Al2SiO5_andalusite` of Section S7.3, which moves no Class I turnover by more than 1.3 K. The polymorph is named here rather than in the Table 1 reaction strings, which write only Al_2SiO_5. The Lackner benchmark rows B070, B074 and B076 use Al_2O_3 as the source product oxide rather than Al_2SiO_5; their deviations therefore include this comparison-basis difference.
$Mg_3Si_2O_5(OH)_4$	298–900 K	The source value is $C_p(298.15) = 274.05$ and the adopted coefficients give 273.75 J mol^{-1} K^{-1}. The source high-temperature table is on p. 394 of the bulletin (Section S6.2).

S2. The complete computed data set

Table S2.1 lists the 23 reactions with the temperature interval over which every participating species has heat-capacity data and with the gas-stoichiometry class of Section 3.2 of the article. Reactions are normalized to one mole of CO_2 consumed, so that reaction properties are comparable across sorbents; the element balances close to a worst residual of 2.2×10^{-16} mol.

The class records reactive-gas stoichiometry, separately from the initial feed. In Class I (18 reactions), CO_2 is the sole reactive gas. For a dry undiluted charge, $y_{CO_2} = 1$ and $G(\xi)$ is linear: its minimum is an endpoint, except at the transition condition where every feasible extent minimizes G. In Class II (four reactions), H_2O(g) is produced, allowing mixing curvature without an added inert; curvature alone does not guarantee an interior minimum. In Class III (R24), water is consumed. An undiluted stoichiometric 1:2 CO_2:H_2O charge retains this ratio, making the ideal-gas quotient constant and $G(\xi)$ linear. Added inert changes the combined reactive-gas fraction with extent and introduces curvature; a non-stoichiometric water charge can also change that geometry. These feed cases do not change the chemical class.

Only R3 and R9 have $\Delta\nu_{gas} = 0$. At fixed initial reactive-gas amounts in the ideal-gas model, total pressure and an added inert cancel from their reaction quotient. They change $G(\xi)$ by an extent-independent term and leave the equilibrium extent unchanged; the five-condition numerical agreement is 6.4×10^{-8}. This cancellation does not apply to changes in the initial water-to-CO_2 ratio or, in general, to nonideal fugacity coefficients.

Table S2.1 The 23 reactions, each written per mole of CO_2 consumed, with the temperature interval over which every participating species has heat-capacity data, and the gas-stoichiometry class of Section 3.2 of the article. Class I: CO_2 is the only gaseous species. Class II: H_2O(g) is a coproduct. Class III: H_2O(g) is a co-reactant. $\Delta\nu_{gas}$ is the net change in the number of gas moles per mole of CO_2 consumed; where it is zero, these quantities are independent of inert loading and total pressure in the ideal-gas model at fixed initial reactive-gas amounts. Changing initial humidity or introducing composition-dependent fugacity coefficients does not satisfy that condition.

ID	Family	Sorbent	Reaction	Cp validity / K low	Cp validity / K high	Class	$\Delta\nu_{gas}$
R1	F1	MgO	$MgO + CO_2 \rightarrow MgCO_3$	298	1000	I	-1
R2	F1	CaO	$CaO + CO_2 \rightarrow CaCO_3$	298	1200	I	-1
R3	F1	$Ca(OH)_2$	$Ca(OH)_2 + CO_2 \rightarrow CaCO_3 + H_2O(g)$	500	1000	II	0
R4	F3	$CaSiO_3$	$CaSiO_3 + CO_2 \rightarrow CaCO_3 + SiO_2$	298	1200	I	-1
R5	F2	Mg_2SiO_4	$\frac{1}{2}Mg_2SiO_4 + CO_2 \rightarrow MgCO_3 + \frac{1}{2}SiO_2$	298	1000	I	-1
R6	F5	Li_4SiO_4	$Li_4SiO_4 + CO_2 \rightarrow Li_2CO_3 + Li_2SiO_3$	298	1300	I	-1
R7	F5	Li_2ZrO_3	$Li_2ZrO_3 + CO_2 \rightarrow Li_2CO_3 + ZrO_2$	298	1400	I	-1
R8	F5	Na_2ZrO_3	$Na_2ZrO_3 + CO_2 \rightarrow Na_2CO_3 + ZrO_2$	298	1170	I	-1
R9	F1	$Mg(OH)_2$	$Mg(OH)_2 + CO_2 \rightarrow MgCO_3 + H_2O(g)$	500	900	II	0
R10	F2	$MgSiO_3$	$MgSiO_3 + CO_2 \rightarrow MgCO_3 + SiO_2$	298	1000	I	-1
R11	F2	$Mg_3Si_2O_5(OH)_4$	$\frac{1}{3}Mg_3Si_2O_5(OH)_4 + CO_2 \rightarrow MgCO_3 + \frac{2}{3}SiO_2 + \frac{2}{3}H_2O(g)$	500	900	II	$-\frac{1}{3}$
R12	F3	Ca_2SiO_4	$\frac{1}{2}Ca_2SiO_4 + CO_2 \rightarrow CaCO_3 + \frac{1}{2}SiO_2$	298	970	I	-1
R13	F3	$CaMgSi_2O_6$	$\frac{1}{2}CaMgSi_2O_6 + CO_2 \rightarrow \frac{1}{2}CaCO_3 + \frac{1}{2}MgCO_3 + SiO_2$	298	1000	I	-1
R14	F4	$CaAl_2Si_2O_8$	$CaAl_2Si_2O_8 + CO_2 \rightarrow CaCO_3 + Al_2SiO_5 + SiO_2$	298	1200	I	-1
R15	F6	$CaCO_3$	$CaCO_3 + MgO + CO_2 \rightarrow CaMg(CO_3)_2$	298	1100	I	-1
R17	F5	Li_2SiO_3	$Li_2SiO_3 + CO_2 \rightarrow Li_2CO_3 + SiO_2$	298	1474	I	-1
R18	F2	$Mg_3Si_4O_{10}(OH)_2$	$\frac{1}{3}Mg_3Si_4O_{10}(OH)_2 + CO_2 \rightarrow MgCO_3 + \frac{4}{3}SiO_2 + \frac{1}{3}H_2O(g)$	500	800	II	$-\frac{2}{3}$
R19	F4	$Ca_3Al_2Si_3O_{12}$	$\frac{1}{3}Ca_3Al_2Si_3O_{12} + CO_2 \rightarrow CaCO_3 + \frac{1}{3}Al_2SiO_5 + \frac{2}{3}SiO_2$	298	1200	I	-1
R20	F4	$Mg_3Al_2Si_3O_{12}$	$\frac{1}{3}Mg_3Al_2Si_3O_{12} + CO_2 \rightarrow MgCO_3 + \frac{1}{3}Al_2SiO_5 + \frac{2}{3}SiO_2$	298	1000	I	-1
R21	F3	$Ca_2MgSi_2O_7$	$\frac{1}{3}Ca_2MgSi_2O_7 + CO_2 \rightarrow \frac{2}{3}CaCO_3 + \frac{1}{3}MgCO_3 + \frac{2}{3}SiO_2$	298	1000	I	-1

ID	Family	Sorbent	Reaction	Cp validity / K low	high	Class	$\Delta\nu_{gas}$
R22	F3	$Ca_3Mg(SiO_4)_2$	$\frac{1}{4}Ca_3Mg(SiO_4)_2 + CO_2 \rightarrow \frac{3}{4}CaCO_3 + \frac{1}{4}MgCO_3 + \frac{1}{2}SiO_2$	298	1000	I	-1
R23	F3	$Ca_3Si_2O_7$	$\frac{1}{3}Ca_3Si_2O_7 + CO_2 \rightarrow CaCO_3 + \frac{2}{3}SiO_2$	298	1200	I	-1
R24	F4	$CaAl_2Si_2O_8$	$CaAl_2Si_2O_8 + CO_2 + 2H_2O(g) \rightarrow CaCO_3 + Al_2Si_2O_5(OH)_4$	500	800	III	-3

Families: F1 Alkaline-earth oxides and hydroxides; F2 Magnesium silicates and the serpentine group; F3 Calcium and calcium-magnesium silicates, including slag phases; F4 Aluminosilicates; F5 Alkali ceramic and carbonate sorbents; F6 Carbonate to double carbonate. Class census: I, 18 reactions; II, 4 (R3, R9, R11, R18); III, 1 (R24). $\Delta\nu_{gas} = 0$ for R3 and R9 only.

Table S2.2 is the full sweep: 23 reactions at 13 temperatures from 298.15 to 1500 K, with standard reaction properties and the equilibrium extent under each of five gas conditions (pure CO_2; 15%, 10% and 4% CO_2 in N_2 at 1 bar total; and 40% CO_2 at 20 bar total). For R24 these labels specify the nominal CO_2:N_2 loading before the stoichiometric water charge is included; the no-inert inlet is $(y_{CO_2}, y_{H_2O}, y_{N_2}) = (1/3, 2/3, 0)$. That is 1495 equilibrium states. The validity flag in the last column states whether the temperature of the row lies inside the interval of Table S2.1. These flags concern heat-capacity coverage, not global phase stability. The restricted gas phase does not allow water condensation; low-temperature hydrous extensions require this additional qualification.

Table S2.2 The complete computed sweep: 23 reactions × 13 temperatures, with the equilibrium extent under each of the five gas conditions, giving 1495 states at the stated reporting precision. ξ_{eq} is the extent at which $G(T,P,\xi)$ is minimum on $0 \leq \xi \leq 1$; the inert loading is $n_{inert} = (1-y_{CO_2})/y_{CO_2}$ per mole of CO_2. For R24 the condition labels specify the nominal CO_2:N_2 loading before the stoichiometric water charge is included; the no-inert inlet is $(y_{CO_2}, y_{H_2O}, y_{N_2}) = (1/3, 2/3, 0)$. The validity flag states whether the temperature lies inside the interval in which every participating species has heat-capacity data. Turnover temperatures are tabulated once per reaction in Tables S2.4 and S2.5.

Rxn	T / K	ΔH° kJ mol^{-1}	ΔS° J mol^{-1}K^{-1}	ΔG° kJ mol^{-1}	$\ln K_{eq}$	ξ_{eq} 100%	15%	10%	4%	40%/20 bar	Validity flag
R1	298.15	-116.57	-175.65	-64.20	25.8992	1.000000	1.000000	1.000000	1.000000	1.000000	IN-RANGE
R1	400.00	-116.19	-174.58	-46.36	13.9397	1.000000	0.999995	0.999992	0.999979	1.000000	IN-RANGE
R1	500.00	-115.41	-172.87	-28.98	6.9715	1.000000	0.994678	0.991548	0.977462	0.999930	IN-RANGE
R1	600.00	-114.21	-170.68	-11.80	2.3656	1.000000	0.412780	0.067356	0.000000	0.992925	IN-RANGE
R1	700.00	-112.60	-168.21	5.15	-0.8842	0.000000	0.000000	0.000000	0.000000	0.793415	IN-RANGE
R1	800.00	-110.64	-165.59	21.84	-3.2831	0.000000	0.000000	0.000000	0.000000	0.000000	IN-RANGE
R1	900.00	-108.36	-162.92	38.26	-5.1135	0.000000	0.000000	0.000000	0.000000	0.000000	IN-RANGE
R1	1000.00	-105.83	-160.25	54.42	-6.5456	0.000000	0.000000	0.000000	0.000000	0.000000	IN-RANGE
R1	1100.00	-103.10	-157.65	70.31	-7.6885	0.000000	0.000000	0.000000	0.000000	0.000000	EXTRAPOLATED-HIGH
R1	1200.00	-100.22	-155.14	85.95	-8.6153	0.000000	0.000000	0.000000	0.000000	0.000000	EXTRAPOLATED-HIGH
R1	1300.00	-97.23	-152.75	101.35	-9.3769	0.000000	0.000000	0.000000	0.000000	0.000000	EXTRAPOLATED-HIGH
R1	1400.00	-94.19	-150.50	116.51	-10.0097	0.000000	0.000000	0.000000	0.000000	0.000000	EXTRAPOLATED-HIGH
R1	1500.00	-91.13	-148.39	131.45	-10.5406	0.000000	0.000000	0.000000	0.000000	0.000000	EXTRAPOLATED-HIGH
R2	298.15	-178.46	-160.29	-130.67	52.7142	1.000000	1.000000	1.000000	1.000000	1.000000	IN-RANGE
R2	400.00	-177.75	-158.26	-114.44	34.4119	1.000000	1.000000	1.000000	1.000000	1.000000	IN-RANGE
R2	500.00	-176.74	-156.03	-98.73	23.7494	1.000000	1.000000	1.000000	1.000000	1.000000	IN-RANGE
R2	600.00	-175.59	-153.93	-83.23	16.6847	1.000000	1.000000	0.999999	0.999999	1.000000	IN-RANGE
R2	700.00	-174.33	-151.99	-67.94	11.6731	1.000000	0.999952	0.999923	0.999796	0.999999	IN-RANGE
R2	800.00	-172.97	-150.18	-52.83	7.9425	1.000000	0.997986	0.996801	0.991469	0.999973	IN-RANGE
R2	900.00	-171.51	-148.46	-37.90	5.0645	1.000000	0.963975	0.942784	0.847423	0.999526	IN-RANGE
R2	1000.00	-169.93	-146.80	-23.13	2.7824	1.000000	0.626152	0.406242	0.000000	0.995344	IN-RANGE
R2	1100.00	-168.21	-145.16	-8.54	0.9333	1.000000	0.000000	0.000000	0.000000	0.969913	IN-RANGE
R2	1200.00	-166.35	-143.55	5.90	-0.5914	0.000000	0.000000	0.000000	0.000000	0.851058	IN-RANGE
R2	1300.00	-164.34	-141.93	20.17	-1.8666	0.000000	0.000000	0.000000	0.000000	0.283362	EXTRAPOLATED-HIGH
R2	1400.00	-162.16	-140.32	34.29	-2.9457	0.000000	0.000000	0.000000	0.000000	0.000000	EXTRAPOLATED-HIGH
R2	1500.00	-159.80	-138.69	48.24	-3.8679	0.000000	0.000000	0.000000	0.000000	0.000000	EXTRAPOLATED-HIGH
R3	298.15	-69.11	-16.60	-64.16	25.8852	1.000000	1.000000	1.000000	1.000000	1.000000	EXTRAPOLATED-LOW
R3	400.00	-69.95	-19.00	-62.35	18.7480	1.000000	1.000000	1.000000	1.000000	1.000000	EXTRAPOLATED-LOW
R3	500.00	-70.81	-20.92	-60.35	14.5175	1.000000	1.000000	1.000000	1.000000	1.000000	IN-RANGE
R3	600.00	-71.68	-22.50	-58.18	11.6622	0.999991	0.999991	0.999991	0.999991	0.999991	IN-RANGE
R3	700.00	-72.54	-23.83	-55.86	9.5978	0.999932	0.999932	0.999932	0.999932	0.999932	IN-RANGE
R3	800.00	-73.39	-24.97	-53.42	8.0310	0.999675	0.999675	0.999675	0.999675	0.999675	IN-RANGE
R3	900.00	-74.20	-25.92	-50.87	6.7984	0.998886	0.998886	0.998886	0.998886	0.998886	IN-RANGE
R3	1000.00	-74.92	-26.68	-48.24	5.8021	0.996988	0.996988	0.996988	0.996988	0.996988	IN-RANGE
R3	1100.00	-75.48	-27.22	-45.54	4.9798	0.993171	0.993171	0.993171	0.993171	0.993171	EXTRAPOLATED-HIGH
R3	1200.00	-75.80	-27.50	-42.80	4.2903	0.986485	0.986485	0.986485	0.986485	0.986485	EXTRAPOLATED-HIGH
R3	1300.00	-75.76	-27.47	-40.05	3.7058	0.976009	0.976009	0.976009	0.976009	0.976009	EXTRAPOLATED-HIGH
R3	1400.00	-75.22	-27.07	-37.32	3.2065	0.961079	0.961079	0.961079	0.961079	0.961079	EXTRAPOLATED-HIGH

continued on the next page

Table S2.2 (continued)

Rxn	T / K	ΔH° kJ mol^{-1}	ΔS° J mol^{-1}K^{-1}	ΔG° kJ mol^{-1}	$\ln K_{eq}$	ξ_{eq} 100%	15%	10%	4%	40%/20 bar	Validity flag
R3	1500.00	-74.02	-26.24	-34.65	2.7787	0.941513	0.941513	0.941513	0.941513	0.941513	EXTRAPOLATED-HIGH
R4	298.15	-89.14	-162.54	-40.68	16.4103	1.000000	1.000000	0.999999	0.999998	1.000000	IN-RANGE
R4	400.00	-88.37	-160.34	-24.23	7.2857	1.000000	0.996114	0.993829	0.983543	0.999949	IN-RANGE
R4	500.00	-87.26	-157.89	-8.32	2.0010	1.000000	0.114073	0.000000	0.000000	0.989791	IN-RANGE
R4	600.00	-85.94	-155.49	7.35	-1.4734	0.000000	0.000000	0.000000	0.000000	0.581351	IN-RANGE
R4	700.00	-84.42	-153.15	22.78	-3.9145	0.000000	0.000000	0.000000	0.000000	0.000000	IN-RANGE
R4	800.00	-82.61	-150.74	37.98	-5.7099	0.000000	0.000000	0.000000	0.000000	0.000000	IN-RANGE
R4	900.00	-80.17	-147.84	52.89	-7.0686	0.000000	0.000000	0.000000	0.000000	0.000000	IN-RANGE
R4	1000.00	-78.75	-146.35	67.60	-8.1310	0.000000	0.000000	0.000000	0.000000	0.000000	IN-RANGE
R4	1100.00	-77.22	-144.89	82.16	-8.9841	0.000000	0.000000	0.000000	0.000000	0.000000	IN-RANGE
R4	1200.00	-75.54	-143.43	96.58	-9.6804	0.000000	0.000000	0.000000	0.000000	0.000000	IN-RANGE
R4	1300.00	-73.69	-141.95	110.85	-10.2560	0.000000	0.000000	0.000000	0.000000	0.000000	EXTRAPOLATED-HIGH
R4	1400.00	-71.67	-140.46	124.97	-10.7366	0.000000	0.000000	0.000000	0.000000	0.000000	EXTRAPOLATED-HIGH
R4	1500.00	-69.47	-138.94	138.94	-11.1410	0.000000	0.000000	0.000000	0.000000	0.000000	EXTRAPOLATED-HIGH
R5	298.15	-86.52	-175.02	-34.34	13.8517	1.000000	0.999995	0.999991	0.999977	1.000000	IN-RANGE
R5	400.00	-86.12	-173.91	-16.56	4.9794	1.000000	0.960754	0.937668	0.833781	0.999484	IN-RANGE
R5	500.00	-85.27	-172.04	0.74	-0.1788	0.000000	0.000000	0.000000	0.000000	0.904614	IN-RANGE
R5	600.00	-83.95	-169.63	17.83	-3.5742	0.000000	0.000000	0.000000	0.000000	0.000000	IN-RANGE
R5	700.00	-82.17	-166.89	34.66	-5.9551	0.000000	0.000000	0.000000	0.000000	0.000000	IN-RANGE
R5	800.00	-79.95	-163.93	51.20	-7.6979	0.000000	0.000000	0.000000	0.000000	0.000000	IN-RANGE
R5	900.00	-77.18	-160.68	67.42	-9.0107	0.000000	0.000000	0.000000	0.000000	0.000000	IN-RANGE
R5	1000.00	-74.79	-158.15	83.36	-10.0269	0.000000	0.000000	0.000000	0.000000	0.000000	IN-RANGE
R5	1100.00	-72.24	-155.72	99.06	-10.8313	0.000000	0.000000	0.000000	0.000000	0.000000	EXTRAPOLATED-HIGH
R5	1200.00	-69.59	-153.42	114.51	-11.4779	0.000000	0.000000	0.000000	0.000000	0.000000	EXTRAPOLATED-HIGH
R5	1300.00	-66.88	-151.25	129.75	-12.0044	0.000000	0.000000	0.000000	0.000000	0.000000	EXTRAPOLATED-HIGH
R5	1400.00	-64.15	-149.23	144.77	-12.4376	0.000000	0.000000	0.000000	0.000000	0.000000	EXTRAPOLATED-HIGH
R5	1500.00	-61.43	-147.35	159.60	-12.7974	0.000000	0.000000	0.000000	0.000000	0.000000	EXTRAPOLATED-HIGH
R6	298.15	-144.28	-165.32	-94.99	38.3192	1.000000	1.000000	1.000000	1.000000	1.000000	IN-RANGE
R6	400.00	-144.80	-167.03	-77.99	23.4499	1.000000	1.000000	1.000000	1.000000	1.000000	IN-RANGE
R6	500.00	-143.43	-164.04	-61.42	14.7740	1.000000	0.999998	0.999997	0.999991	1.000000	IN-RANGE
R6	600.00	-141.10	-159.81	-45.22	9.0643	1.000000	0.999344	0.998958	0.997222	0.999991	IN-RANGE
R6	700.00	-135.62	-151.54	-29.54	5.0760	1.000000	0.964390	0.943443	0.849181	0.999531	IN-RANGE
R6	800.00	-135.04	-150.78	-14.42	2.1686	1.000000	0.268471	0.000000	0.000000	0.991376	IN-RANGE
R6	900.00	-134.31	-149.91	0.61	-0.0816	0.000000	0.000000	0.000000	0.000000	0.913953	IN-RANGE
R6	1000.00	-88.86	-104.10	15.24	-1.8335	0.000000	0.000000	0.000000	0.000000	0.317289	IN-RANGE
R6	1100.00	-89.86	-105.04	25.69	-2.8088	0.000000	0.000000	0.000000	0.000000	0.000000	IN-RANGE
R6	1200.00	-92.64	-107.45	36.30	-3.6385	0.000000	0.000000	0.000000	0.000000	0.000000	IN-RANGE
R6	1300.00	-97.22	-111.11	47.22	-4.3688	0.000000	0.000000	0.000000	0.000000	0.000000	IN-RANGE
R6	1400.00	-103.63	-115.85	58.56	-5.0309	0.000000	0.000000	0.000000	0.000000	0.000000	EXTRAPOLATED-HIGH
R6	1500.00	-111.86	-121.52	70.42	-5.6466	0.000000	0.000000	0.000000	0.000000	0.000000	EXTRAPOLATED-HIGH
R7	298.15	-162.78	-165.00	-113.58	45.8221	1.000000	1.000000	1.000000	1.000000	1.000000	IN-RANGE
R7	400.00	-162.82	-165.18	-96.75	29.0912	1.000000	1.000000	1.000000	1.000000	1.000000	IN-RANGE
R7	500.00	-161.78	-162.90	-80.32	19.3225	1.000000	1.000000	1.000000	1.000000	1.000000	IN-RANGE
R7	600.00	-159.32	-158.47	-64.24	12.8772	1.000000	0.999986	0.999977	0.999939	1.000000	IN-RANGE
R7	700.00	-152.73	-148.52	-48.77	8.3796	1.000000	0.998699	0.997934	0.994491	0.999983	IN-RANGE

continued on the next page

Table S2.2 (continued)

Rxn	T / K	ΔH° kJ mol^{-1}	ΔS° J mol^{-1}K^{-1}	ΔG° kJ mol^{-1}	$\ln K_{eq}$	ξ_{eq} 100%	15%	10%	4%	40%/20 bar	Validity flag
R7	800.00	-149.81	-144.65	-34.10	5.1263	1.000000	0.966147	0.946234	0.856623	0.999554	IN-RANGE
R7	900.00	-145.34	-139.40	-19.88	2.6573	1.000000	0.572565	0.321132	0.000000	0.994721	IN-RANGE
R7	1000.00	-94.65	-88.09	-6.57	0.7900	1.000000	0.000000	0.000000	0.000000	0.965173	IN-RANGE
R7	1100.00	-88.86	-82.56	1.96	-0.2141	0.000000	0.000000	0.000000	0.000000	0.900958	IN-RANGE
R7	1200.00	-83.24	-77.67	9.97	-0.9988	0.000000	0.000000	0.000000	0.000000	0.764378	IN-RANGE
R7	1300.00	-77.79	-73.31	17.51	-1.6201	0.000000	0.000000	0.000000	0.000000	0.492824	IN-RANGE
R7	1400.00	-72.49	-69.38	24.64	-2.1170	0.000000	0.000000	0.000000	0.000000	0.000000	IN-RANGE
R7	1500.00	-61.51	-61.88	31.31	-2.5107	0.000000	0.000000	0.000000	0.000000	0.000000	EXTRAPOLATED-HIGH
R8	298.15	-151.40	-153.81	-105.54	42.5771	1.000000	1.000000	1.000000	1.000000	1.000000	IN-RANGE
R8	400.00	-151.01	-152.68	-89.94	27.0434	1.000000	1.000000	1.000000	1.000000	1.000000	IN-RANGE
R8	500.00	-150.20	-150.92	-74.74	17.9803	1.000000	1.000000	1.000000	1.000000	1.000000	IN-RANGE
R8	600.00	-148.22	-147.35	-59.81	11.9905	1.000000	0.999965	0.999944	0.999851	1.000000	IN-RANGE
R8	700.00	-144.44	-141.56	-45.35	7.7922	1.000000	0.997659	0.996282	0.990085	0.999969	IN-RANGE
R8	800.00	-141.96	-138.15	-31.44	4.7267	1.000000	0.949369	0.919586	0.785562	0.999336	IN-RANGE
R8	900.00	-140.19	-136.09	-17.72	2.3677	1.000000	0.414172	0.069567	0.000000	0.992940	IN-RANGE
R8	1000.00	-137.20	-132.95	-4.26	0.5120	1.000000	0.000000	0.000000	0.000000	0.953664	IN-RANGE
R8	1100.00	-132.96	-128.92	8.84	-0.9670	0.000000	0.000000	0.000000	0.000000	0.772891	IN-RANGE
R8	1200.00	-98.62	-98.42	19.49	-1.9536	0.000000	0.000000	0.000000	0.000000	0.182666	EXTRAPOLATED-HIGH
R8	1300.00	-94.11	-94.81	29.15	-2.6970	0.000000	0.000000	0.000000	0.000000	0.000000	EXTRAPOLATED-HIGH
R8	1400.00	-89.76	-91.59	38.47	-3.3048	0.000000	0.000000	0.000000	0.000000	0.000000	EXTRAPOLATED-HIGH
R8	1500.00	-79.89	-84.85	47.39	-3.8003	0.000000	0.000000	0.000000	0.000000	0.000000	EXTRAPOLATED-HIGH
R9	298.15	-35.49	-23.06	-28.62	11.5453	0.999990	0.999990	0.999990	0.999990	0.999990	EXTRAPOLATED-LOW
R9	400.00	-36.27	-25.27	-26.16	7.8672	0.999617	0.999617	0.999617	0.999617	0.999617	EXTRAPOLATED-LOW
R9	500.00	-37.18	-27.29	-23.53	5.6603	0.996530	0.996530	0.996530	0.996530	0.996530	IN-RANGE
R9	600.00	-37.91	-28.64	-20.73	4.1551	0.984558	0.984558	0.984558	0.984558	0.984558	IN-RANGE
R9	700.00	-38.38	-29.37	-17.82	3.0624	0.955313	0.955313	0.955313	0.955313	0.955313	IN-RANGE
R9	800.00	-38.57	-29.63	-14.87	2.2355	0.903394	0.903394	0.903394	0.903394	0.903394	IN-RANGE
R9	900.00	-38.50	-29.54	-11.91	1.5914	0.830814	0.830814	0.830814	0.830814	0.830814	IN-RANGE
R9	1000.00	-38.19	-29.22	-8.97	1.0786	0.746238	0.746238	0.746238	0.746238	0.746238	EXTRAPOLATED-HIGH
R9	1100.00	-37.70	-28.75	-6.07	0.6635	0.660048	0.660048	0.660048	0.660048	0.660048	EXTRAPOLATED-HIGH
R9	1200.00	-37.06	-28.20	-3.22	0.3227	0.579991	0.579991	0.579991	0.579991	0.579991	EXTRAPOLATED-HIGH
R9	1300.00	-36.33	-27.61	-0.43	0.0397	0.509922	0.509923	0.509923	0.509922	0.509922	EXTRAPOLATED-HIGH
R9	1400.00	-35.55	-27.04	2.30	-0.1979	0.450688	0.450688	0.450688	0.450688	0.450688	EXTRAPOLATED-HIGH
R9	1500.00	-34.76	-26.50	4.98	-0.3993	0.401480	0.401480	0.401480	0.401480	0.401480	EXTRAPOLATED-HIGH
R10	298.15	-83.27	-173.50	-31.54	12.7228	1.000000	0.999983	0.999973	0.999928	1.000000	IN-RANGE
R10	400.00	-83.02	-172.82	-13.89	4.1775	1.000000	0.911741	0.859823	0.626195	0.998849	IN-RANGE
R10	500.00	-82.37	-171.38	3.32	-0.7993	0.000000	0.000000	0.000000	0.000000	0.812342	IN-RANGE
R10	600.00	-81.28	-169.40	20.37	-4.0825	0.000000	0.000000	0.000000	0.000000	0.000000	IN-RANGE
R10	700.00	-79.73	-167.03	37.19	-6.3903	0.000000	0.000000	0.000000	0.000000	0.000000	IN-RANGE
R10	800.00	-77.67	-164.28	53.76	-8.0826	0.000000	0.000000	0.000000	0.000000	0.000000	IN-RANGE
R10	900.00	-74.82	-160.91	70.00	-9.3556	0.000000	0.000000	0.000000	0.000000	0.000000	IN-RANGE
R10	1000.00	-72.96	-158.96	86.00	-10.3435	0.000000	0.000000	0.000000	0.000000	0.000000	IN-RANGE
R10	1100.00	-71.11	-157.19	101.80	-11.1315	0.000000	0.000000	0.000000	0.000000	0.000000	EXTRAPOLATED-HIGH
R10	1200.00	-69.40	-155.70	117.44	-11.7717	0.000000	0.000000	0.000000	0.000000	0.000000	EXTRAPOLATED-HIGH
R10	1300.00	-68.04	-154.61	132.96	-12.3014	0.000000	0.000000	0.000000	0.000000	0.000000	EXTRAPOLATED-HIGH
R10	1400.00	-67.25	-154.03	148.38	-12.7481	0.000000	0.000000	0.000000	0.000000	0.000000	EXTRAPOLATED-HIGH

continued on the next page

Table S2.2 (continued)

Rxn	T / K	ΔH° kJ mol^{-1}	ΔS° J mol^{-1}K^{-1}	ΔG° kJ mol^{-1}	$\ln K_{eq}$	ξ_{eq} 100%	15%	10%	4%	40%/20 bar	Validity flag
R10	1500.00	-67.33	-154.08	163.78	-13.1330	0.000000	0.000000	0.000000	0.000000	0.000000	EXTRAPOLATED-HIGH
R11	298.15	-33.19	-68.93	-12.63	5.0965	0.995930	0.991410	0.990119	0.986531	0.997777	EXTRAPOLATED-LOW
R11	400.00	-33.25	-69.11	-5.60	1.6854	0.884012	0.777782	0.750433	0.680843	0.935291	EXTRAPOLATED-LOW
R11	500.00	-33.30	-69.23	1.31	-0.3162	0.431970	0.240683	0.208576	0.147188	0.627388	IN-RANGE
R11	600.00	-33.19	-69.03	8.23	-1.6500	0.108281	0.045639	0.037710	0.024338	0.240321	IN-RANGE
R11	700.00	-32.78	-68.41	15.11	-2.5958	0.029364	0.011632	0.009527	0.006056	0.077031	IN-RANGE
R11	800.00	-31.93	-67.29	21.90	-3.2921	0.010601	0.004139	0.003383	0.002144	0.029149	IN-RANGE
R11	900.00	-30.39	-65.46	28.53	-3.8124	0.004895	0.001903	0.001554	0.000984	0.013663	IN-RANGE
R11	1000.00	-29.28	-64.30	35.02	-4.2117	0.002697	0.001047	0.000855	0.000541	0.007572	EXTRAPOLATED-HIGH
R11	1100.00	-27.90	-62.98	41.38	-4.5248	0.001688	0.000655	0.000535	0.000338	0.004754	EXTRAPOLATED-HIGH
R11	1200.00	-26.33	-61.61	47.61	-4.7721	0.001166	0.000452	0.000369	0.000233	0.003287	EXTRAPOLATED-HIGH
R11	1300.00	-24.69	-60.31	53.71	-4.9689	0.000868	0.000336	0.000275	0.000174	0.002450	EXTRAPOLATED-HIGH
R11	1400.00	-23.18	-59.19	59.68	-5.1271	0.000685	0.000265	0.000217	0.000137	0.001934	EXTRAPOLATED-HIGH
R11	1500.00	-22.02	-58.39	65.55	-5.2564	0.000564	0.000219	0.000179	0.000113	0.001594	EXTRAPOLATED-HIGH
R12	298.15	-115.38	-165.15	-66.14	26.6821	1.000000	1.000000	1.000000	1.000000	1.000000	IN-RANGE
R12	400.00	-114.62	-163.00	-49.42	14.8610	1.000000	0.999998	0.999997	0.999992	1.000000	IN-RANGE
R12	500.00	-113.53	-160.57	-33.24	7.9971	1.000000	0.998093	0.996971	0.991923	0.999975	IN-RANGE
R12	600.00	-112.30	-158.34	-17.30	3.4681	1.000000	0.817651	0.710386	0.227697	0.997658	IN-RANGE
R12	700.00	-110.98	-156.31	-1.57	0.2697	1.000000	0.000000	0.000000	0.000000	0.940456	IN-RANGE
R12	800.00	-109.53	-154.37	13.96	-2.0995	0.000000	0.000000	0.000000	0.000000	0.000000	IN-RANGE
R12	900.00	-107.75	-152.26	29.29	-3.9141	0.000000	0.000000	0.000000	0.000000	0.000000	IN-RANGE
R12	1000.00	-106.41	-150.85	44.44	-5.3456	0.000000	0.000000	0.000000	0.000000	0.000000	EXTRAPOLATED-HIGH
R12	1100.00	-104.86	-149.38	59.46	-6.5012	0.000000	0.000000	0.000000	0.000000	0.000000	EXTRAPOLATED-HIGH
R12	1200.00	-102.99	-147.75	74.31	-7.4487	0.000000	0.000000	0.000000	0.000000	0.000000	EXTRAPOLATED-HIGH
R12	1300.00	-100.66	-145.89	89.00	-8.2343	0.000000	0.000000	0.000000	0.000000	0.000000	EXTRAPOLATED-HIGH
R12	1400.00	-97.73	-143.72	103.48	-8.8905	0.000000	0.000000	0.000000	0.000000	0.000000	EXTRAPOLATED-HIGH
R12	1500.00	-94.03	-141.18	117.73	-9.4403	0.000000	0.000000	0.000000	0.000000	0.000000	EXTRAPOLATED-HIGH
R13	298.15	-75.72	-165.28	-26.45	10.6689	1.000000	0.999868	0.999791	0.999442	0.999998	IN-RANGE
R13	400.00	-75.15	-163.65	-9.69	2.9141	1.000000	0.674937	0.483723	0.000000	0.995920	IN-RANGE
R13	500.00	-74.24	-161.62	6.58	-1.5817	0.000000	0.000000	0.000000	0.000000	0.518068	IN-RANGE
R13	600.00	-72.99	-159.35	22.63	-4.5357	0.000000	0.000000	0.000000	0.000000	0.000000	IN-RANGE
R13	700.00	-71.39	-156.90	38.44	-6.6050	0.000000	0.000000	0.000000	0.000000	0.000000	IN-RANGE
R13	800.00	-69.37	-154.21	54.00	-8.1185	0.000000	0.000000	0.000000	0.000000	0.000000	IN-RANGE
R13	900.00	-66.60	-150.93	69.24	-9.2535	0.000000	0.000000	0.000000	0.000000	0.000000	IN-RANGE
R13	1000.00	-64.79	-149.02	84.24	-10.1320	0.000000	0.000000	0.000000	0.000000	0.000000	IN-RANGE
R13	1100.00	-62.81	-147.14	99.05	-10.8301	0.000000	0.000000	0.000000	0.000000	0.000000	EXTRAPOLATED-HIGH
R13	1200.00	-60.67	-145.28	113.67	-11.3931	0.000000	0.000000	0.000000	0.000000	0.000000	EXTRAPOLATED-HIGH
R13	1300.00	-58.37	-143.44	128.10	-11.8524	0.000000	0.000000	0.000000	0.000000	0.000000	EXTRAPOLATED-HIGH
R13	1400.00	-55.93	-141.64	142.36	-12.2303	0.000000	0.000000	0.000000	0.000000	0.000000	EXTRAPOLATED-HIGH
R13	1500.00	-53.38	-139.87	156.43	-12.5436	0.000000	0.000000	0.000000	0.000000	0.000000	EXTRAPOLATED-HIGH
R14	298.15	-83.88	-197.12	-25.11	10.1291	1.000000	0.999774	0.999641	0.999042	0.999997	IN-RANGE
R14	400.00	-83.27	-195.42	-5.10	1.5339	1.000000	0.000000	0.000000	0.000000	0.983646	IN-RANGE
R14	500.00	-82.02	-192.66	14.31	-3.4416	0.000000	0.000000	0.000000	0.000000	0.000000	IN-RANGE
R14	600.00	-80.38	-189.67	33.42	-6.7002	0.000000	0.000000	0.000000	0.000000	0.000000	IN-RANGE
R14	700.00	-78.44	-186.68	52.24	-8.9764	0.000000	0.000000	0.000000	0.000000	0.000000	IN-RANGE

continued on the next page

Table S2.2 (continued)

Rxn	T / K	ΔH° kJ mol^{-1}	ΔS° J mol^{-1}K^{-1}	ΔG° kJ mol^{-1}	$\ln K_{eq}$	ξ_{eq} 100%	15%	10%	4%	40%/20 bar	Validity flag
R14	800.00	-76.17	-183.67	70.76	-10.6385	0.000000	0.000000	0.000000	0.000000	0.000000	IN-RANGE
R14	900.00	-73.31	-180.28	88.94	-11.8862	0.000000	0.000000	0.000000	0.000000	0.000000	IN-RANGE
R14	1000.00	-71.57	-178.45	106.87	-12.8547	0.000000	0.000000	0.000000	0.000000	0.000000	IN-RANGE
R14	1100.00	-69.88	-176.83	124.64	-13.6283	0.000000	0.000000	0.000000	0.000000	0.000000	IN-RANGE
R14	1200.00	-68.25	-175.42	142.25	-14.2578	0.000000	0.000000	0.000000	0.000000	0.000000	IN-RANGE
R14	1300.00	-66.73	-174.20	159.73	-14.7782	0.000000	0.000000	0.000000	0.000000	0.000000	EXTRAPOLATED-HIGH
R14	1400.00	-65.36	-173.18	177.09	-15.2147	0.000000	0.000000	0.000000	0.000000	0.000000	EXTRAPOLATED-HIGH
R14	1500.00	-64.20	-172.38	194.37	-15.5857	0.000000	0.000000	0.000000	0.000000	0.000000	EXTRAPOLATED-HIGH
R15	298.15	-122.48	-177.26	-69.63	28.0886	1.000000	1.000000	1.000000	1.000000	1.000000	IN-RANGE
R15	400.00	-122.39	-177.04	-51.58	15.5091	1.000000	0.999999	0.999998	0.999996	1.000000	IN-RANGE
R15	500.00	-121.92	-176.01	-33.92	8.1594	1.000000	0.998379	0.997425	0.993133	0.999979	IN-RANGE
R15	600.00	-121.03	-174.38	-16.40	3.2867	1.000000	0.779966	0.650534	0.068091	0.997191	IN-RANGE
R15	700.00	-119.76	-172.43	0.95	-0.1628	0.000000	0.000000	0.000000	0.000000	0.906222	IN-RANGE
R15	800.00	-118.18	-170.33	18.09	-2.7193	0.000000	0.000000	0.000000	0.000000	0.000000	IN-RANGE
R15	900.00	-116.32	-168.14	35.01	-4.6790	0.000000	0.000000	0.000000	0.000000	0.000000	IN-RANGE
R15	1000.00	-114.17	-165.88	51.71	-6.2200	0.000000	0.000000	0.000000	0.000000	0.000000	IN-RANGE
R15	1100.00	-111.67	-163.50	68.18	-7.4555	0.000000	0.000000	0.000000	0.000000	0.000000	IN-RANGE
R15	1200.00	-108.70	-160.92	84.41	-8.4603	0.000000	0.000000	0.000000	0.000000	0.000000	EXTRAPOLATED-HIGH
R15	1300.00	-105.10	-158.04	100.36	-9.2853	0.000000	0.000000	0.000000	0.000000	0.000000	EXTRAPOLATED-HIGH
R15	1400.00	-100.65	-154.75	116.00	-9.9661	0.000000	0.000000	0.000000	0.000000	0.000000	EXTRAPOLATED-HIGH
R15	1500.00	-95.05	-150.89	131.29	-10.5275	0.000000	0.000000	0.000000	0.000000	0.000000	EXTRAPOLATED-HIGH
R17	298.15	-84.98	-161.78	-36.74	14.8223	1.000000	0.999998	0.999997	0.999991	1.000000	IN-RANGE
R17	400.00	-84.58	-160.65	-20.32	6.1091	1.000000	0.987377	0.979952	0.946539	0.999833	IN-RANGE
R17	500.00	-83.49	-158.26	-4.36	1.0481	1.000000	0.000000	0.000000	0.000000	0.973235	IN-RANGE
R17	600.00	-81.13	-154.01	11.27	-2.2600	0.000000	0.000000	0.000000	0.000000	0.000000	IN-RANGE
R17	700.00	-74.70	-144.30	26.31	-4.5206	0.000000	0.000000	0.000000	0.000000	0.000000	IN-RANGE
R17	800.00	-71.88	-140.55	40.57	-6.0990	0.000000	0.000000	0.000000	0.000000	0.000000	IN-RANGE
R17	900.00	-67.10	-134.93	54.34	-7.2615	0.000000	0.000000	0.000000	0.000000	0.000000	IN-RANGE
R17	1000.00	-17.41	-84.67	67.26	-8.0894	0.000000	0.000000	0.000000	0.000000	0.000000	IN-RANGE
R17	1100.00	-12.77	-80.24	75.49	-8.2549	0.000000	0.000000	0.000000	0.000000	0.000000	IN-RANGE
R17	1200.00	-8.46	-76.49	83.33	-8.3520	0.000000	0.000000	0.000000	0.000000	0.000000	IN-RANGE
R17	1300.00	-4.43	-73.26	90.81	-8.4019	0.000000	0.000000	0.000000	0.000000	0.000000	IN-RANGE
R17	1400.00	-0.65	-70.46	97.99	-8.4189	0.000000	0.000000	0.000000	0.000000	0.000000	IN-RANGE
R17	1500.00	2.89	-68.02	104.91	-8.4126	0.000000	0.000000	0.000000	0.000000	0.000000	EXTRAPOLATED-HIGH
R18	298.15	-46.38	-117.40	-11.37	4.5880	0.996597	0.976851	0.969013	0.941879	0.998566	EXTRAPOLATED-LOW
R18	400.00	-46.31	-117.22	0.57	-0.1722	0.501106	0.036302	0.017036	0.002840	0.836043	EXTRAPOLATED-LOW
R18	500.00	-45.86	-116.24	12.26	-2.9480	0.000432	0.000010	0.000004	0.000001	0.025949	IN-RANGE
R18	600.00	-44.87	-114.44	23.79	-4.7696	0.000002	0.000000	0.000000	0.000000	0.000117	IN-RANGE
R18	700.00	-43.68	-112.60	35.14	-6.0384	0.000000	0.000000	0.000000	0.000000	0.000003	IN-RANGE
R18	800.00	-42.71	-111.29	46.33	-6.9658	0.000000	0.000000	0.000000	0.000000	0.000000	IN-RANGE
R18	900.00	-42.14	-110.59	57.39	-7.6701	0.000000	0.000000	0.000000	0.000000	0.000000	EXTRAPOLATED-HIGH
R18	1000.00	-44.87	-113.45	68.58	-8.2484	0.000000	0.000000	0.000000	0.000000	0.000000	EXTRAPOLATED-HIGH
R18	1100.00	-50.06	-118.37	80.15	-8.7640	0.000000	0.000000	0.000000	0.000000	0.000000	EXTRAPOLATED-HIGH
R18	1200.00	-58.34	-125.55	92.33	-9.2542	0.000000	0.000000	0.000000	0.000000	0.000000	EXTRAPOLATED-HIGH
R18	1300.00	-70.34	-135.14	105.34	-9.7464	0.000000	0.000000	0.000000	0.000000	0.000000	EXTRAPOLATED-HIGH
R18	1400.00	-86.72	-147.26	119.44	-10.2615	0.000000	0.000000	0.000000	0.000000	0.000000	EXTRAPOLATED-HIGH

continued on the next page

Table S2.2 (continued)

Rxn	T / K	ΔH° kJ mol^{-1}	ΔS° J mol^{-1}K^{-1}	ΔG° kJ mol^{-1}	$\ln K_{eq}$	ξ_{eq} 100%	15%	10%	4%	40%/20 bar	Validity flag
R18	1500.00	-108.12	-162.00	134.88	-10.8155	0.000000	0.000000	0.000000	0.000000	0.000000	EXTRAPOLATED-HIGH
R19	298.15	-71.78	-153.54	-26.00	10.4887	1.000000	0.999842	0.999749	0.999332	0.999998	IN-RANGE
R19	400.00	-70.85	-150.88	-10.49	3.1551	1.000000	0.747636	0.599187	0.000000	0.996795	IN-RANGE
R19	500.00	-69.64	-148.19	4.46	-1.0729	0.000000	0.000000	0.000000	0.000000	0.743169	IN-RANGE
R19	600.00	-68.25	-145.67	19.15	-3.8393	0.000000	0.000000	0.000000	0.000000	0.000000	IN-RANGE
R19	700.00	-66.66	-143.23	33.60	-5.7729	0.000000	0.000000	0.000000	0.000000	0.000000	IN-RANGE
R19	800.00	-64.79	-140.73	47.80	-7.1861	0.000000	0.000000	0.000000	0.000000	0.000000	IN-RANGE
R19	900.00	-62.39	-137.89	61.72	-8.2480	0.000000	0.000000	0.000000	0.000000	0.000000	IN-RANGE
R19	1000.00	-60.61	-136.02	75.41	-9.0704	0.000000	0.000000	0.000000	0.000000	0.000000	IN-RANGE
R19	1100.00	-58.75	-134.25	88.92	-9.7234	0.000000	0.000000	0.000000	0.000000	0.000000	IN-RANGE
R19	1200.00	-56.90	-132.64	102.27	-10.2505	0.000000	0.000000	0.000000	0.000000	0.000000	IN-RANGE
R19	1300.00	-55.18	-131.26	115.46	-10.6826	0.000000	0.000000	0.000000	0.000000	0.000000	EXTRAPOLATED-HIGH
R19	1400.00	-53.77	-130.22	128.53	-11.0425	0.000000	0.000000	0.000000	0.000000	0.000000	EXTRAPOLATED-HIGH
R19	1500.00	-52.89	-129.61	141.52	-11.3477	0.000000	0.000000	0.000000	0.000000	0.000000	EXTRAPOLATED-HIGH
R20	298.15	-94.90	-182.21	-40.57	16.3687	1.000000	1.000000	0.999999	0.999998	1.000000	IN-RANGE
R20	400.00	-94.58	-181.31	-22.05	6.6312	1.000000	0.992518	0.988117	0.968311	0.999901	IN-RANGE
R20	500.00	-93.75	-179.48	-4.01	0.9640	1.000000	0.000000	0.000000	0.000000	0.970841	IN-RANGE
R20	600.00	-92.44	-177.11	13.83	-2.7716	0.000000	0.000000	0.000000	0.000000	0.000000	IN-RANGE
R20	700.00	-90.66	-174.38	31.40	-5.3959	0.000000	0.000000	0.000000	0.000000	0.000000	IN-RANGE
R20	800.00	-88.39	-171.35	48.69	-7.3207	0.000000	0.000000	0.000000	0.000000	0.000000	IN-RANGE
R20	900.00	-85.44	-167.88	65.64	-8.7728	0.000000	0.000000	0.000000	0.000000	0.000000	IN-RANGE
R20	1000.00	-82.99	-165.29	82.30	-9.8992	0.000000	0.000000	0.000000	0.000000	0.000000	IN-RANGE
R20	1100.00	-80.31	-162.74	98.70	-10.7926	0.000000	0.000000	0.000000	0.000000	0.000000	EXTRAPOLATED-HIGH
R20	1200.00	-77.46	-160.26	114.85	-11.5119	0.000000	0.000000	0.000000	0.000000	0.000000	EXTRAPOLATED-HIGH
R20	1300.00	-74.45	-157.86	130.76	-12.0980	0.000000	0.000000	0.000000	0.000000	0.000000	EXTRAPOLATED-HIGH
R20	1400.00	-71.36	-155.56	146.43	-12.5801	0.000000	0.000000	0.000000	0.000000	0.000000	EXTRAPOLATED-HIGH
R20	1500.00	-68.22	-153.40	161.87	-12.9800	0.000000	0.000000	0.000000	0.000000	0.000000	EXTRAPOLATED-HIGH
R21	298.15	-100.51	-174.14	-48.59	19.6011	1.000000	1.000000	1.000000	1.000000	1.000000	IN-RANGE
R21	400.00	-99.91	-172.47	-30.92	9.2987	1.000000	0.999481	0.999176	0.997803	0.999993	IN-RANGE
R21	500.00	-98.76	-169.93	-13.80	3.3196	1.000000	0.787363	0.662282	0.099419	0.997283	IN-RANGE
R21	600.00	-97.21	-167.11	3.05	-0.6121	0.000000	0.000000	0.000000	0.000000	0.847630	IN-RANGE
R21	700.00	-95.33	-164.22	19.62	-3.3712	0.000000	0.000000	0.000000	0.000000	0.000000	IN-RANGE
R21	800.00	-93.13	-161.29	35.90	-5.3968	0.000000	0.000000	0.000000	0.000000	0.000000	IN-RANGE
R21	900.00	-90.44	-158.11	51.85	-6.9299	0.000000	0.000000	0.000000	0.000000	0.000000	IN-RANGE
R21	1000.00	-88.42	-155.97	67.56	-8.1256	0.000000	0.000000	0.000000	0.000000	0.000000	IN-RANGE
R21	1100.00	-86.33	-153.98	83.05	-9.0814	0.000000	0.000000	0.000000	0.000000	0.000000	EXTRAPOLATED-HIGH
R21	1200.00	-84.19	-152.12	98.36	-9.8586	0.000000	0.000000	0.000000	0.000000	0.000000	EXTRAPOLATED-HIGH
R21	1300.00	-81.99	-150.36	113.48	-10.4995	0.000000	0.000000	0.000000	0.000000	0.000000	EXTRAPOLATED-HIGH
R21	1400.00	-79.72	-148.68	128.43	-11.0341	0.000000	0.000000	0.000000	0.000000	0.000000	EXTRAPOLATED-HIGH
R21	1500.00	-77.39	-147.07	143.22	-11.4842	0.000000	0.000000	0.000000	0.000000	0.000000	EXTRAPOLATED-HIGH
R22	298.15	-110.88	-171.28	-59.81	24.1284	1.000000	1.000000	1.000000	1.000000	1.000000	IN-RANGE
R22	400.00	-110.22	-169.42	-42.45	12.7654	1.000000	0.999984	0.999974	0.999931	1.000000	IN-RANGE
R22	500.00	-109.17	-167.10	-25.63	6.1643	1.000000	0.988057	0.981032	0.949418	0.999842	IN-RANGE
R22	600.00	-107.84	-164.68	-9.04	1.8114	1.000000	0.000000	0.000000	0.000000	0.987642	IN-RANGE
R22	700.00	-106.25	-162.23	7.31	-1.2560	0.000000	0.000000	0.000000	0.000000	0.680576	IN-RANGE

continued on the next page

Table S2.2 (continued)

Rxn	T / K	ΔH° kJ mol^{-1}	ΔS° J mol^{-1}K^{-1}	ΔG° kJ mol^{-1}	$\ln K_{eq}$	ξ_{eq} 100%	15%	10%	4%	40%/20 bar	Validity flag
R22	800.00	-104.38	-159.74	23.41	-3.5195	0.000000	0.000000	0.000000	0.000000	0.000000	IN-RANGE
R22	900.00	-102.08	-157.02	39.24	-5.2440	0.000000	0.000000	0.000000	0.000000	0.000000	IN-RANGE
R22	1000.00	-100.21	-155.05	54.84	-6.5964	0.000000	0.000000	0.000000	0.000000	0.000000	IN-RANGE
R22	1100.00	-98.21	-153.15	70.25	-7.6816	0.000000	0.000000	0.000000	0.000000	0.000000	EXTRAPOLATED-HIGH
R22	1200.00	-96.10	-151.31	85.47	-8.5672	0.000000	0.000000	0.000000	0.000000	0.000000	EXTRAPOLATED-HIGH
R22	1300.00	-93.88	-149.53	100.52	-9.2999	0.000000	0.000000	0.000000	0.000000	0.000000	EXTRAPOLATED-HIGH
R22	1400.00	-91.59	-147.84	115.38	-9.9130	0.000000	0.000000	0.000000	0.000000	0.000000	EXTRAPOLATED-HIGH
R22	1500.00	-89.24	-146.22	130.08	-10.4310	0.000000	0.000000	0.000000	0.000000	0.000000	EXTRAPOLATED-HIGH
R23	298.15	-104.18	-164.64	-55.09	22.2251	1.000000	1.000000	1.000000	1.000000	1.000000	IN-RANGE
R23	400.00	-103.37	-162.35	-38.43	11.5563	1.000000	0.999946	0.999914	0.999770	0.999999	IN-RANGE
R23	500.00	-102.20	-159.74	-22.33	5.3710	1.000000	0.973531	0.957961	0.887895	0.999651	IN-RANGE
R23	600.00	-100.81	-157.22	-6.48	1.2992	1.000000	0.000000	0.000000	0.000000	0.979261	IN-RANGE
R23	700.00	-99.24	-154.81	9.12	-1.5670	0.000000	0.000000	0.000000	0.000000	0.527333	IN-RANGE
R23	800.00	-97.43	-152.39	24.48	-3.6805	0.000000	0.000000	0.000000	0.000000	0.000000	IN-RANGE
R23	900.00	-95.13	-149.67	39.57	-5.2886	0.000000	0.000000	0.000000	0.000000	0.000000	IN-RANGE
R23	1000.00	-93.43	-147.88	54.45	-6.5491	0.000000	0.000000	0.000000	0.000000	0.000000	IN-RANGE
R23	1100.00	-91.55	-146.09	69.15	-7.5609	0.000000	0.000000	0.000000	0.000000	0.000000	IN-RANGE
R23	1200.00	-89.46	-144.27	83.67	-8.3861	0.000000	0.000000	0.000000	0.000000	0.000000	IN-RANGE
R23	1300.00	-87.12	-142.40	98.00	-9.0672	0.000000	0.000000	0.000000	0.000000	0.000000	EXTRAPOLATED-HIGH
R23	1400.00	-84.51	-140.47	112.14	-9.6347	0.000000	0.000000	0.000000	0.000000	0.000000	EXTRAPOLATED-HIGH
R23	1500.00	-81.64	-138.49	126.09	-10.1108	0.000000	0.000000	0.000000	0.000000	0.000000	EXTRAPOLATED-HIGH
R24	298.15	-214.73	-498.65	-66.05	26.6478	1.000000	0.999504	0.999213	0.997901	0.999993	EXTRAPOLATED-LOW
R24	400.00	-212.58	-492.57	-15.55	4.6757	1.000000	0.000000	0.000000	0.000000	0.989856	EXTRAPOLATED-LOW
R24	500.00	-209.32	-485.33	33.35	-8.0215	0.000000	0.000000	0.000000	0.000000	0.000000	IN-RANGE
R24	600.00	-205.57	-478.51	81.53	-16.3441	0.000000	0.000000	0.000000	0.000000	0.000000	IN-RANGE
R24	700.00	-201.72	-472.56	129.08	-22.1789	0.000000	0.000000	0.000000	0.000000	0.000000	IN-RANGE
R24	800.00	-198.01	-467.61	176.08	-26.4729	0.000000	0.000000	0.000000	0.000000	0.000000	IN-RANGE
R24	900.00	-194.70	-463.71	222.63	-29.7535	0.000000	0.000000	0.000000	0.000000	0.000000	EXTRAPOLATED-HIGH
R24	1000.00	-192.03	-460.88	268.85	-32.3375	0.000000	0.000000	0.000000	0.000000	0.000000	EXTRAPOLATED-HIGH
R24	1100.00	-190.22	-459.16	314.85	-34.4268	0.000000	0.000000	0.000000	0.000000	0.000000	EXTRAPOLATED-HIGH
R24	1200.00	-189.55	-458.56	360.72	-36.1562	0.000000	0.000000	0.000000	0.000000	0.000000	EXTRAPOLATED-HIGH
R24	1300.00	-190.27	-459.13	406.60	-37.6193	0.000000	0.000000	0.000000	0.000000	0.000000	EXTRAPOLATED-HIGH
R24	1400.00	-192.67	-460.90	452.59	-38.8835	0.000000	0.000000	0.000000	0.000000	0.000000	EXTRAPOLATED-HIGH
R24	1500.00	-197.06	-463.92	498.82	-39.9983	0.000000	0.000000	0.000000	0.000000	0.000000	EXTRAPOLATED-HIGH

Table S2.3 is the 500 K slice of the same sweep, expressed as equilibrium CO_2 conversion. At 15% CO_2 nine of the 23 reactions reach at least 99.5% conversion and nine are inactive; the intermediate cases are the informative ones, and none of them can be read off a table of ΔG°.

Table S2.3 Equilibrium CO_2 conversion (%) at 500 K for all 23 reactions under the five gas conditions. This is the single-temperature slice of Table S2.2 that Section 5.3 of the article refers to.

ID	Fam.	Sorbent	Reaction	100%	15%	10%	4%	40%/20 bar	Validity flag
					conversion at 500 K / %				
R1	F1	MgO	$MgO + CO_2 \rightarrow MgCO_3$	100.0	99.5	99.2	97.7	100.0	IN-RANGE
R2	F1	CaO	$CaO + CO_2 \rightarrow CaCO_3$	100.0	100.0	100.0	100.0	100.0	IN-RANGE
R3	F1	$Ca(OH)_2$	$Ca(OH)_2 + CO_2 \rightarrow CaCO_3 + H_2O(g)$	100.0	100.0	100.0	100.0	100.0	IN-RANGE
R4	F3	$CaSiO_3$	$CaSiO_3 + CO_2 \rightarrow CaCO_3 + SiO_2$	100.0	11.4	0.0	0.0	99.0	IN-RANGE
R5	F2	Mg_2SiO_4	$\frac{1}{2}Mg_2SiO_4 + CO_2 \rightarrow MgCO_3 + \frac{1}{2}SiO_2$	0.0	0.0	0.0	0.0	90.5	IN-RANGE
R6	F5	Li_4SiO_4	$Li_4SiO_4 + CO_2 \rightarrow Li_2CO_3 + Li_2SiO_3$	100.0	100.0	100.0	100.0	100.0	IN-RANGE
R7	F5	Li_2ZrO_3	$Li_2ZrO_3 + CO_2 \rightarrow Li_2CO_3 + ZrO_2$	100.0	100.0	100.0	100.0	100.0	IN-RANGE
R8	F5	Na_2ZrO_3	$Na_2ZrO_3 + CO_2 \rightarrow Na_2CO_3 + ZrO_2$	100.0	100.0	100.0	100.0	100.0	IN-RANGE
R9	F1	$Mg(OH)_2$	$Mg(OH)_2 + CO_2 \rightarrow MgCO_3 + H_2O(g)$	99.7	99.7	99.7	99.7	99.7	IN-RANGE
R10	F2	$MgSiO_3$	$MgSiO_3 + CO_2 \rightarrow MgCO_3 + SiO_2$	0.0	0.0	0.0	0.0	81.2	IN-RANGE
R11	F2	$Mg_3Si_2O_5(OH)_4$	$\frac{1}{3}Mg_3Si_2O_5(OH)_4 + CO_2 \rightarrow MgCO_3 + \frac{2}{3}SiO_2 + \frac{2}{3}H_2O(g)$	43.2	24.1	20.9	14.7	62.7	IN-RANGE
R12	F3	Ca_2SiO_4	$\frac{1}{2}Ca_2SiO_4 + CO_2 \rightarrow CaCO_3 + \frac{1}{2}SiO_2$	100.0	99.8	99.7	99.2	100.0	IN-RANGE
R13	F3	$CaMgSi_2O_6$	$\frac{1}{2}CaMgSi_2O_6 + CO_2 \rightarrow \frac{1}{2}CaCO_3 + \frac{1}{2}MgCO_3 + SiO_2$	0.0	0.0	0.0	0.0	51.8	IN-RANGE
R14	F4	$CaAl_2Si_2O_8$	$CaAl_2Si_2O_8 + CO_2 \rightarrow CaCO_3 + Al_2SiO_5 + SiO_2$	0.0	0.0	0.0	0.0	0.0	IN-RANGE
R15	F6	$CaCO_3$	$CaCO_3 + MgO + CO_2 \rightarrow CaMg(CO_3)_2$	100.0	99.8	99.7	99.3	100.0	IN-RANGE
R17	F5	Li_2SiO_3	$Li_2SiO_3 + CO_2 \rightarrow Li_2CO_3 + SiO_2$	100.0	0.0	0.0	0.0	97.3	IN-RANGE
R18	F2	$Mg_3Si_4O_{10}(OH)_2$	$\frac{1}{3}Mg_3Si_4O_{10}(OH)_2 + CO_2 \rightarrow MgCO_3 + \frac{4}{3}SiO_2 + \frac{1}{3}H_2O(g)$	0.0	0.0	0.0	0.0	2.6	IN-RANGE
R19	F4	$Ca_3Al_2Si_3O_{12}$	$\frac{1}{3}Ca_3Al_2Si_3O_{12} + CO_2 \rightarrow CaCO_3 + \frac{1}{3}Al_2SiO_5 + \frac{2}{3}SiO_2$	0.0	0.0	0.0	0.0	74.3	IN-RANGE
R20	F4	$Mg_3Al_2Si_3O_{12}$	$\frac{1}{3}Mg_3Al_2Si_3O_{12} + CO_2 \rightarrow MgCO_3 + \frac{1}{3}Al_2SiO_5 + \frac{2}{3}SiO_2$	100.0	0.0	0.0	0.0	97.1	IN-RANGE
R21	F3	$Ca_2MgSi_2O_7$	$\frac{1}{3}Ca_2MgSi_2O_7 + CO_2 \rightarrow \frac{2}{3}CaCO_3 + \frac{1}{3}MgCO_3 + \frac{2}{3}SiO_2$	100.0	78.7	66.2	9.9	99.7	IN-RANGE
R22	F3	$Ca_3Mg(SiO_4)_2$	$\frac{1}{4}Ca_3Mg(SiO_4)_2 + CO_2 \rightarrow \frac{3}{4}CaCO_3 + \frac{1}{4}MgCO_3 + \frac{1}{2}SiO_2$	100.0	98.8	98.1	94.9	100.0	IN-RANGE
R23	F3	$Ca_3Si_2O_7$	$\frac{1}{3}Ca_3Si_2O_7 + CO_2 \rightarrow CaCO_3 + \frac{2}{3}SiO_2$	100.0	97.4	95.8	88.8	100.0	IN-RANGE
R24	F4	$CaAl_2Si_2O_8$	$CaAl_2Si_2O_8 + CO_2 + 2H_2O(g) \rightarrow CaCO_3 + Al_2Si_2O_5(OH)_4$	0.0	0.0	0.0	0.0	0.0	IN-RANGE

Table S2.4 gives the turnover temperature at four CO_2 partial pressures. For the 18 Class I reactions it solves $\Delta G^\circ(T) = RT\ln(P_{CO_2}/P^\circ)$ and is a function of P_{CO_2} alone. For the four Class II and the one Class III reaction the reaction quotient also contains $H_2O(g)$, the turnover temperature is undefined without a specified water activity, and the 17 rooted values listed for them are the $p_{H_2O} = 1$ bar members of $\Delta G^\circ + RT\ln Q = 0$; Table S2.5 gives the same values under four further water conventions. Two bases are reported for every value. The primary basis carries molten Li_2CO_3 above 993 K and molten Na_2CO_3 above 1123 K, as the source data do.

The solid-only basis suppresses both melts as a metastable phase diagnostic. It isolates part of the phase contribution to comparison differences, but does not reproduce every structure or thermochemical assumption in a first-principles calculation.

Every temperature in Table S2.4 is displayed to 0.1 K, which is the resolution of the root-finding and not a statement about how well the temperature is known. The $u(T_{turn})$ column of Table 1 of the article propagates the standard formation-enthalpy uncertainty recorded for each adopted anchor, giving conditional enthalpy-uncertainty contributions of 3.4–13.8 K for the three reactions with complete species coverage (R1, R2 and R4). The remaining fifteen reactions have missing uncertainty inputs. Their partial contributions span 0.9–15.2 K and are not complete uncertainty estimates; no pooled uncertainty median is used. Entropy, heat-capacity and covariance uncertainties are not included. One basis is used per species: for magnesite it is the ± 0.47 kJ mol^{-1} of the Koziol–Newton reversed-equilibrium determination that corroborates the adopted anchor, while the ± 8 kJ mol^{-1} of the 1966 JANAF assessment is carried separately as a scenario in Section S7.3. Three reactions, R1, R2 and R4, have a printed uncertainty for every participating species. For the other fifteen at least one species carries none, so those entries are lower bounds. The species without a printed uncertainty are Mg_2SiO_4, $MgSiO_3$, Ca_2SiO_4, $CaMgSi_2O_6$, $CaAl_2Si_2O_8$, Al_2SiO_5, $Ca_3Al_2Si_3O_{12}$, $Mg_3Al_2Si_3O_{12}$, $Ca_2MgSi_2O_7$, $Ca_3Mg(SiO_4)_2$, $Ca_3Si_2O_7$, $CaMg(CO_3)_2$, Li_4SiO_4, Li_2ZrO_3, Li_2SiO_3, Li_2CO_3, Na_2CO_3 and ZrO_2.

Sixteen of the 92 turnover values lie outside the common heat-capacity interval: six are flagged XH and ten XL. These are conditional extrapolations, not predictions validated over the displayed temperature range. The portlandite values at 0.1 and 0.15 bar, 1633.4 and 1775.9 K, extrapolate a 298–1000 K fit by 633 and 776 K. The XL flag marks values below 500 K, where the adopted NIST-JANAF $H_2O(g)$ interval begins. Agreement of the extrapolated heat capacity with the tabulated values to 0.0041% at 300 K and 0.0033% at 400 K is a local consistency check and does not establish the validity of the complete low-temperature phase model.

For the full reaction set, including the provisional cases, at $P_{CO_2} = 1$ bar the Class I turnover temperatures span 426.1 K for anorthite to 1159.0 K for lime. The family medians are 914.2 K for the alkaline-earth oxides and hydroxides (F1), 488.1 K for the magnesium silicates (F2), 611.6 K for the calcium and calcium-magnesium silicates (F3), 470.0 K for the aluminosilicates (F4), 964.0 K for the alkali ceramics (F5) and 694.5 K for dolomite formation (F6). Over the same 18 reactions the 0.15 to 20 bar turnover span has a median of 165 K, a minimum of 94.7 K for anorthite and a maximum of 738.5 K for Li_2ZrO_3. The largest value involves a high-temperature extrapolation and is a diagnostic, not an operating recommendation.

Table S2.4 Turnover temperatures T_{turn} at four CO_2 partial pressures, on the primary basis (which carries molten Li_2CO_3 above 993 K and molten Na_2CO_3 above 1123 K) and on the solid-only basis, which is the like-for-like basis against solid-phase first-principles screening values. For the 18 Class I reactions T_{turn} solves $\Delta G^\circ(T) = RT\ln(P_{CO_2}/P^\circ)$ and is a function of P_{CO_2} alone. For the four Class II and the one Class III reaction $H_2O(g)$ enters the reaction quotient, the turnover temperature is undefined without a specified water activity, and every value listed here is the $p_{H_2O} = 1$ bar member of $\Delta G^\circ + RT\ln Q = 0$; Table S2.5 gives the same reactions under four other water conventions. Flags: IR inside the heat-capacity validity window and quotable; XH extrapolated above the data and *not* quotable as a result; XL below 500 K where the NIST-JANAF $H_2O(g)$ range begins, where the extrapolated heat capacity agrees with the tabulated value to 0.0041% at 300 K and 0.0033% at 400 K; "–" no root in 300–2000 K.

ID	Sorbent	Class	0.1 bar			0.15 bar			1 bar			20 bar		
			primary	flag	solid	primary	flag	solid	primary	flag	solid	primary	flag	solid
R1	MgO	I	601.7	IR	601.7	612.5	IR	612.5	669.5	IR	669.5	786.4	IR	786.4
R2	CaO	I	1024.1	IR	1024.1	1045.4	IR	1045.4	1159.0	IR	1159.0	1405.0	XH	1405.0
R3	$Ca(OH)_2$	II	1633.4	XH	1633.4	1775.9	XH	1775.9	–	NO-ROOT-300-2000K	–	–	NO-ROOT-300-2000K	–
R4	$CaSiO_3$	I	492.9	IR	492.9	502.5	IR	502.5	552.9	IR	552.9	658.5	IR	658.5
R5	Mg_2SiO_4	I	446.2	IR	446.2	454.2	IR	454.2	495.7	IR	495.7	580.1	IR	580.1
R6	Li_4SiO_4	I	794.8	IR	794.8	810.8	IR	810.8	895.9	IR	895.9	1121.3	IR	1075.5
R7	Li_2ZrO_3	I	916.8	IR	916.8	936.9	IR	936.9	1076.5	IR	1047.6	1675.4	XH	1327.1
R8	Na_2ZrO_3	I	903.1	IR	903.1	923.2	IR	923.2	1032.2	IR	1032.2	1346.7	XH	1286.6
R9	$Mg(OH)_2$	II	790.9	IR	790.9	849.6	IR	849.6	1315.6	XH	1315.6	–	NO-ROOT-300-2000K	–
R10	$MgSiO_3$	I	432.5	IR	432.5	440.3	IR	440.3	480.6	IR	480.6	562.6	IR	562.6
R11	$Mg_3Si_2O_5(OH)_4$	II	376.7	XL	376.7	391.7	XL	391.7	481.0	XL	481.0	753.8	IR	753.8
R12	Ca_2SiO_4	I	632.8	IR	632.8	645.1	IR	645.1	710.0	IR	710.0	846.2	IR	846.2
R13	$CaMgSi_2O_6$	I	411.1	IR	411.1	418.9	IR	418.9	459.4	IR	459.4	543.1	IR	543.1
R14	$CaAl_2Si_2O_8$	I	388.1	IR	388.1	394.3	IR	394.3	426.1	IR	426.1	489.0	IR	489.0
R15	$CaCO_3$	I	625.4	IR	625.4	636.5	IR	636.5	694.5	IR	694.5	812.7	IR	812.7
R17	Li_2SiO_3	I	470.7	IR	470.7	479.8	IR	479.8	527.6	IR	527.6	628.6	IR	628.6
R18	$Mg_3Si_4O_{10}(OH)_2$	II	339.7	XL	339.7	348.3	XL	348.3	395.1	XL	395.1	502.2	IR	502.2
R19	$Ca_3Al_2Si_3O_{12}$	I	416.7	IR	416.7	425.2	IR	425.2	470.0	IR	470.0	565.3	IR	565.3
R20	$Mg_3Al_2Si_3O_{12}$	I	472.0	IR	472.0	480.2	IR	480.2	522.4	IR	522.4	607.3	IR	607.3
R21	$Ca_2MgSi_2O_7$	I	522.4	IR	522.4	531.9	IR	531.9	581.8	IR	581.8	684.3	IR	684.3
R22	$Ca_3Mg(SiO_4)_2$	I	586.7	IR	586.7	597.6	IR	597.6	655.1	IR	655.1	774.2	IR	774.2
R23	$Ca_3Si_2O_7$	I	571.7	IR	571.7	582.8	IR	582.8	641.4	IR	641.4	764.4	IR	764.4
R24	$CaAl_2Si_2O_8$	III	415.4	XL	415.4	418.2	XL	418.2	431.6	XL	431.6	454.8	XL	454.8

Table S2.5 collects the five reactions that produce or consume $H_2O(g)$ and gives the turnover temperature of each under five stated water conventions. The point of the table is that no single number exists to be quoted for these reactions. The unit-water-pressure convention included in Table 2 of the article and in Table S2.4, $p_{H_2O} = 1$ bar, is the one recorded in the source locators of the published values compared against in Section S3, so those comparisons remain matched. It is nevertheless a choice. The spread across the conventions is 524.7 K for brucite at $P_{CO_2} = 0.1$ bar, 555.6 K for chrysotile at 20 bar and as much as 653.7 K for brucite at $P_{CO_2} = 0.15$ bar. Portlandite has a root below 2000 K only in the steam-rich case in which water is held at 1 bar while CO_2 sits at 0.1 or 0.15 bar. For the two reactions with $\Delta\nu_{gas} = 0$ the unit-ratio condition $p_{H_2O} = p_{CO_2}$ is pressure independent within the ideal-gas model: 1315.6 K for brucite at every CO_2 pressure, coinciding with T_{50} of an initially dry closed charge. This value exceeds the 900 K upper limit of the brucite heat-capacity data.

Table S2.5 The five reactions that produce or consume $H_2O(g)$, with the turnover temperature under five stated water conventions. A turnover temperature is not defined for these reactions by P_{CO_2} alone: each column is a different member of the general condition $\Delta G^\circ(T) + RT \ln Q = 0$. Columns: $p_{H_2O} = 1$ bar, the convention of the values quoted in Table 2 of the article and in Table S2.4 and also the convention recorded in the locators of the published sources compared against; $p_{H_2O} = 0.1$ and 0.01 bar, fixed water partial pressures representing comparatively humid and dry conditions; $p_{H_2O} = p_{CO_2}$, the unit-ratio condition, which for $\Delta\nu_{gas} = 0$ is independent of pressure; and the self-generated atmosphere, the 50% conversion temperature of an initially dry closed CO_2 charge at the listed total pressure; the CO_2 partial pressure then changes with conversion. This feed cannot convert a reaction that consumes water. Superscripts L and H flag extrapolation below or above the common heat-capacity interval, respectively, and W marks an entry whose stated water partial pressure exceeds the saturation pressure at that temperature, so that the root lies inside the liquid-water field. "–" means no root in 300–2000 K, and "> 2000" means the 50% crossing lies above 2000 K. The spread column is left blank where fewer than two conventions give a root.

ID	Class	P_{CO_2} / bar	T_{turn} / K under the stated water convention: p_{H_2O} = 1 bar	p_{H_2O} = 0.1 bar	p_{H_2O} = 0.01 bar	$p_{H_2O} = p_{CO_2}$	self-generated	spread / K
R3	II	0.1	1633.4^{H}	–	–	–	> 2000	–
R3	II	0.15	1775.9^{H}	–	–	–	> 2000	–
R3	II	1	–	–	–	–	> 2000	–
R3	II	20	–	–	–	–	> 2000	–
R9	II	0.1	790.9	1315.6^{H}	–	1315.6^{H}	1315.56^{H}	524.7
R9	II	0.15	849.6	1503.3^{H}	–	1315.6^{H}	1315.56^{H}	653.7
R9	II	1	1315.6^{H}	–	–	1315.6^{H}	1315.56^{H}	0.0
R9	II	20	–	–	–	1315.6^{H}	1315.56^{H}	0.0
R11	II	0.1	376.7^{L}	440.4^{L}	529.9	440.4^{L}	445.31^{L}	153.2
R11	II	0.15	391.7^{L}	461.0^{L}	559.9	447.1^{L}	452.11^{L}	168.2
R11	II	1	481.0^{L}	589.8	764.8	481.0^{L}	486.87^{L}	283.8
R11	II	20	753.8	1102.2^{H}	–	546.6	554.14	555.6
R18	II	0.1	339.7^{LW}	356.3^{L}	374.7^{L}	356.3^{L}	360.35^{L}	35.0
R18	II	0.15	348.3^{LW}	365.8^{L}	385.2^{L}	362.6^{L}	366.76^{L}	36.9
R18	II	1	395.1^{L}	417.9^{L}	443.4^{L}	395.1^{L}	400.06^{L}	48.3
R18	II	20	502.2	540.0	584.5	460.4^{LW}	467.20^{L}	124.1
R24	III	0.1	415.4^{L}	386.5^{L}	361.4^{L}	386.5^{L}	–	54.0
R24	III	0.15	418.2^{L}	388.9^{L}	363.5^{L}	393.7^{L}	–	54.7
R24	III	1	431.6^{L}	400.4^{L}	373.5^{L}	431.6^{L}	–	58.1
R24	III	20	454.8^{L}	420.2^{L}	390.6^{L}	509.8	–	119.2

R3 and R9 have $\Delta\nu_{gas} = 0$: their unit-ratio column is independent of P_{CO_2}, and for brucite it coincides with the initially dry closed-charge value of 1315.6 K, above the brucite heat-capacity limit. The two R3 entries that exist at $p_{H_2O} = 1$ bar do so only because water is held at 1 bar while CO_2 sits at 0.1 or 0.15 bar, that is, in a steam-rich atmosphere; under every other convention tried portlandite has no turnover below 2000 K. Brucite at $P_{CO_2} = 0.1$ bar moves by 524.7 K between the first two columns, and its largest spread over the five conventions, 653.7 K, occurs at $P_{CO_2} = 0.15$ bar. Both hydroxides dehydroxylate below the temperatures tabulated here, at 537.6 K for brucite and 795.3 K for portlandite at $p_{H_2O} = 1$ bar (Section 5.6 of the article). Values shown to one decimal place, except the self-generated column, which retains two decimal places from the numerical calculation.

Table S2.6 gives the 50% conversion temperatures used to construct Fig. S2 and the full-set diagnostic dilution statistics. R6 and R7 are provisional and excluded from the primary aggregate of Table S7.8. The full-set median 100.5 K and maximum 229.8 K shifts for the 18 Class I reactions can be checked directly. Table S2.6 is generated from the same target grid and rounding rule as Table S7.1, so the two agree cell by cell. Pure-feed entries denote the degenerate transition boundary. Section S7.3 tests the dependence of these statistics on thermochemical sources and carbonate phases.

Table S2.6 Temperature at which equilibrium conversion passes 50% under the five gas conditions, used for Fig. S2 and the dilution-shift statistics. Superscripts L and H flag extrapolation below or above the common heat-capacity interval. Dry pure-feed Class I entries and the undiluted stoichiometric R24 entry denote a degenerate phase boundary, not a uniquely selected 50% conversion. For R24 the condition labels specify the nominal CO_2:N_2 loading before the stoichiometric water charge is added; the actual three-component inlet compositions are stated in the article. "> 2000" means that the 50% crossing lies above 2000 K, so equilibrium conversion stays above 50% throughout the tabulated window.

ID	Sorbent	Family	Pure, 1 bar	15%, 1 bar	10%, 1 bar	4%, 1 bar	40%, 20 bar
					T_{50} / K		
R1	MgO	F1	669.5	596.2	585.2	562.6	727.3
R2	CaO	F1	1159.0	1013.4	992.1	948.6	1278.4^{H}
R3	$Ca(OH)_2$	F1	> 2000	> 2000	> 2000	> 2000	> 2000
R4	$CaSiO_3$	F3	552.9	488.1	478.5	458.8	604.8
R5	Mg_2SiO_4	F2	495.7	442.2	434.2	417.5	537.6
R6	Li_4SiO_4	F5	895.9	786.7	770.6	737.5	984.0
R7	Li_2ZrO_3	F5	1076.5	906.8	886.9	846.7	1298.1
R8	Na_2ZrO_3	F5	1032.2	893.1	873.2	832.6	1160.0
R9	$Mg(OH)_2$	F1	1315.6^{H}	1315.6^{H}	1315.6^{H}	1315.6^{H}	1315.6^{H}
R10	$MgSiO_3$	F2	480.6	428.6	420.8	404.6	521.3
R11	$Mg_3Si_2O_5(OH)_4$	F2	486.9^{L}	449.5^{L}	442.6^{L}	428.0^{L}	529.0
R12	Ca_2SiO_4	F3	710.0	626.6	614.3	588.9	776.9
R13	$CaMgSi_2O_6$	F3	459.4	407.2	399.5	383.4	500.8
R14	$CaAl_2Si_2O_8$	F4	426.1	385.0	378.7	365.6	457.7
R15	$CaCO_3 + MgO$	F6	694.5	619.8	608.6	585.4	753.1
R17	Li_2SiO_3	F5	527.6	466.1	457.0	438.3	577.0
R18	$Mg_3Si_4O_{10}(OH)_2$	F2	400.1^{L}	361.2^{L}	354.7^{L}	341.1^{L}	438.2^{L}
R19	$Ca_3Al_2Si_3O_{12}$	F4	470.0	412.5	404.0	386.6	516.6
R20	$Mg_3Al_2Si_3O_{12}$	F4	522.4	467.9	459.7	442.7	564.7
R21	$Ca_2MgSi_2O_7$	F3	581.8	517.6	508.0	488.3	632.5
R22	$Ca_3Mg(SiO_4)_2$	F3	655.1	581.2	570.2	547.5	713.8
R23	$Ca_3Si_2O_7$	F3	641.4	566.1	555.0	532.1	701.7
R24	$CaAl_2Si_2O_8$	F4	418.1^{L}	388.3^{L}	381.7^{L}	367.1^{L}	471.7^{L}

S3. The benchmark atlas

The atlas joins every published turnover temperature, decomposition temperature (the term some cited sources use for the same zero crossing) and standard reaction property that could be located for the reactions in this set to the value computed here. It contains 112 rows drawn from 11 source documents, of which 59 enter the statistics as 33 temperature and 26 energy comparisons; the rows that do not are excluded for a stated reason carried in the row itself.

Every row carries a benchmark class, and the classes are the point of the table. An *independent first-principles* comparison takes the published value from a density functional theory and lattice-dynamics calculation, and an *independent experimental* comparison takes it from measurement; these are classified separately from assessed-data comparisons, although individual ancillary data and phase assumptions can still overlap. A *secondary-source* comparison is a value quoted at second hand. A *shared-data-lineage* comparison is one in which the published value derives from the same assessed compilation that supplies this work's own 298 K reference frame, so agreement with it verifies consistency with that source and is not a test of whether the underlying data are right. Six distinct data lineages are represented across the 11 documents.

Two conventions apply to the Published column of Table S3.1. Nine of the rows drawn from Robie and Hemingway, B079 to B101, are comparison temperatures obtained in this work by interpolating that source's tabulated Gibbs energies to the stated condition. They are not temperatures printed in the bulletin, and they are marked SHARED-LIN for that reason. Where a source publishes a bracket rather than a point, rows B103 to B105 and B110, the signed deviation is measured to the nearer bracket edge and is zero anywhere inside the bracket. The CSV retains the midpoint difference in a separate field. The same convention is stated in the caption of Fig. 4 of the article.

The shared-data-lineage median $|\Delta T_{\mathrm{turn}}|$ is 6.6 K ($n = 18$), compared with 67.7 K for the combined independent categories ($n = 14$). These are descriptive summaries of heterogeneous comparisons, not pooled measures of predictive accuracy. The 11 first-principles temperature comparisons have a median absolute deviation of 71.7 K; three are above the adopted heat-capacity ranges. The eight in-range rows have a median of 79.0 K. The three experimental temperature comparisons included in the statistics all concern CaO and are extrapolated above its common heat-capacity range. Their 65.5 K median therefore does not constitute validation across materials or diluted gas mixtures. The main article shows the individual first-principles deviations as well as the source-class summaries.

Table S3.1 Condensed benchmark atlas, one row per published value: all 112 located rows, of which 59 enter the statistics. The displayed fields retain the benchmark identifier, reaction, source, quantity, published and computed values, deviation, absolute deviation, benchmark class, inclusion flag and reason or qualification. Classes: IND-DFT, independent first-principles comparison; IND-EXP, independent experimental comparison; SEC-SRC, value quoted at second hand; and SHARED-LIN, comparison sharing the assessed-data lineage used for the 298 K anchors. For a point datum the displayed signed deviation is computed minus published. For a published interval it is the signed distance to the nearer edge outside the interval, or zero inside; the absolute column uses its magnitude. The CSV retains the midpoint difference in a separate field. Superscripts H and L mark computed values above or below a participating heat-capacity data window; they are diagnostic extrapolations.

Row	Rxn	Source	Quantity	Published	Computed	Dev.	|Dev.|	Class	Used	Note / reason not used
B001	R1	Duan & Sorescu, J. Chem. Phys. 133, 074508 (2010)	turnover temperature at P_CO2 = 20 bar	690	786.4	96.4	96.4	IND-DFT	yes	
B002	R1	Duan & Sorescu, J. Chem. Phys. 133, 074508 (2010)	turnover temperature at P_CO2 = 0.1 bar	540	601.7	61.7	61.7	IND-DFT	yes	
B003	R2	Duan & Sorescu, J. Chem. Phys. 133, 074508 (2010)	turnover temperature at P_CO2 = 20 bar	1350	1405.0^{H}	55.0	55.0	IND-DFT	yes	
B004	R2	Duan & Sorescu, J. Chem. Phys. 133, 074508 (2010)	turnover temperature at P_CO2 = 0.1 bar	1010	1024.1	14.1	14.1	IND-DFT	yes	
B005	R3	Duan & Sorescu, J. Chem. Phys. 133, 074508 (2010)	turnover temperature at P_CO2 = 20 bar	>1500	–	–	–	IND-DFT	no	flagged in this work as not usable in deviation statistics
B006	R3	Duan & Sorescu, J. Chem. Phys. 133, 074508 (2010)	turnover temperature at P_CO2 = 0.1 bar	>1500	1633.4^{H}	–	–	IND-DFT	no	flagged in this work as not usable in deviation statistics
B007	R9	Duan & Sorescu, J. Chem. Phys. 133, 074508 (2010)	turnover temperature at P_CO2 = 20 bar	>1500	–	–	–	IND-DFT	no	flagged in this work as not usable in deviation statistics
B008	R9	Duan & Sorescu, J. Chem. Phys. 133, 074508 (2010)	turnover temperature at P_CO2 = 0.1 bar	600	790.9	190.9	190.9	IND-DFT	yes	
B009	R2	Duan & Sorescu, J. Chem. Phys. 133, 074508 (2010)	turnover temperature at P_CO2 = 1 bar	~1200	1159.0	-41.0	41.0	IND-DFT	no	flagged in this work as not usable in deviation statistics
B010	R9	Duan & Sorescu, J. Chem. Phys. 133, 074508 (2010)	turnover temperature at P_CO2 = 1 bar	750	1315.6^{H}	565.6	565.6	IND-DFT	no	flagged in this work as not usable in deviation statistics
B011	R2	Stanmore & Gilot, Fuel Process. Technol. 86, 1707 (2005)	turnover temperature at P_CO2 = 1 bar	1123	1159.0	36.0	36.0	SEC-SRC	yes	
B012	R2	Stanmore & Gilot, Fuel Process. Technol. 86, 1707 (2005)	turnover temperature at P_CO2 = 1 bar	1173	1159.0	-14.0	14.0	SEC-SRC	no	flagged in this work as not usable in deviation statistics
B013	R7	Duan, J. Renew. Sustain. Energy 3, 013102 (2011)	turnover temperature at P_CO2 = 20 bar	1000	1675.4^{H}	675.4	675.4	IND-DFT	yes	
B014	R7	Duan, J. Renew. Sustain. Energy 3, 013102 (2011)	turnover temperature at P_CO2 = 0.1 bar	780	916.8	136.8	136.8	IND-DFT	yes	
B015	R7	Duan, J. Renew. Sustain. Energy 3, 013102 (2011)	dH_rxn(300 K)	-158.562	-162.79	-4.22	4.22	IND-DFT	yes	
B016	R7	Duan, J. Renew. Sustain. Energy 3, 013102 (2011)	dG_rxn(300 K)	-103.845	-113.28	-9.43	9.43	IND-DFT	yes	
B017	R7	HSC Chemistry 6.1 database, as tabulated by Duan (2011)	dH_rxn(300 K)	-162.69	-162.79	-0.10	0.10	SHARED-LIN	no	flagged in this work as not usable in deviation statistics

continued on the next page

Table S3.1 (continued)

Row	Rxn	Source	Quantity	Published	Computed	Dev.	|Dev.|	Class	Used	Note / reason not used
B018	R7	HSC Chemistry 6.1 database, as tabulated by Duan (2011)	dG_rxn(300 K)	-113.18	-113.28	-0.10	0.10	SHARED-LIN	no	flagged in this work as not usable in deviation statistics
B019	R7	Duan, J. Renew. Sustain. Energy 3, 013102 (2011) [Li6Zr2O7, 1/3 Li6Zr2O7 + CO2 route]	turnover temperature at P_CO2 = 0.1 bar	880	916.8	36.8	36.8	IND-DFT	no	flagged in this work as not usable in deviation statistics
B020	R6	Duan, Luebke & Pennline, Int. J. Clean Coal Energy 1, 1 (2012)	turnover temperature at P_CO2 = 20 bar	1010	1121.3	111.3	111.3	IND-DFT	yes	
B021	R6	Duan, Luebke & Pennline, Int. J. Clean Coal Energy 1, 1 (2012)	turnover temperature at P_CO2 = 0.1 bar	770	794.8	24.8	24.8	IND-DFT	yes	
B022	R6	Duan, Luebke & Pennline, Int. J. Clean Coal Energy 1, 1 (2012)	dH_rxn(300 K)	-143.548	-144.32	-0.77	0.77	IND-DFT	yes	
B023	R6	Duan, Luebke & Pennline, Int. J. Clean Coal Energy 1, 1 (2012)	dG_rxn(300 K)	-93.972	-94.68	-0.71	0.71	IND-DFT	yes	
B024	R8	Duan, Luebke & Pennline, Int. J. Clean Coal Energy 1, 1 (2012)	turnover temperature at P_CO2 = 20 bar	1275	1346.7^{H}	71.7	71.7	IND-DFT	yes	
B025	R8	Duan, Luebke & Pennline, Int. J. Clean Coal Energy 1, 1 (2012)	turnover temperature at P_CO2 = 0.1 bar	925	903.1	-21.9	21.9	IND-DFT	yes	
B026	R8	Duan, Luebke & Pennline, Int. J. Clean Coal Energy 1, 1 (2012)	dH_rxn(300 K)	-158.327	-151.39	6.94	6.94	IND-DFT	yes	
B027	R8	Duan, Luebke & Pennline, Int. J. Clean Coal Energy 1, 1 (2012)	dG_rxn(300 K)	-114.121	-105.26	8.86	8.86	IND-DFT	yes	
B028	R8	HSC Chemistry database, as tabulated by Duan et al. (2012)	dH_rxn(300 K)	-151.403	-151.39	0.01	0.01	SHARED-LIN	no	flagged in this work as not usable in deviation statistics
B029	R8	HSC Chemistry database, as tabulated by Duan et al. (2012)	dG_rxn(300 K)	-105.252	-105.26	-0.01	0.01	SHARED-LIN	no	flagged in this work as not usable in deviation statistics
B030	R7	Duan, Luebke & Pennline, Int. J. Clean Coal Energy 1, 1 (2012)	no CO2 partial pressure resolvable from the conditions field	1000 / 780	–	–	–	IND-DFT	no	flagged in this work as not usable in deviation statistics
B031	R1	Duan, Luebke & Pennline, Int. J. Clean Coal Energy 1, 1 (2012)	dH_rxn(300 K)	-112.254	-116.56	-4.31	4.31	IND-DFT	yes	
B032	R1	Duan, Luebke & Pennline, Int. J. Clean Coal Energy 1, 1 (2012)	dG_rxn(300 K)	-58.867	-63.88	-5.01	5.01	IND-DFT	yes	

continued on the next page

Table S3.1 (continued)

Row	Rxn	Source	Quantity	Published	Computed	Dev.	|Dev.|	Class	Used	Note / reason not used
B033	R1	HSC Chemistry / FactSage database, as tabulated by Duan et al. (2012)	dH_rxn(300 K)	-100.891	-116.56	-15.67	15.67	SHARED-LIN	no	flagged in this work as not usable in deviation statistics
B034	R1	HSC Chemistry / FactSage database, as tabulated by Duan et al. (2012)	dG_rxn(300 K)	-48.206	-63.88	-15.67	15.67	SHARED-LIN	no	flagged in this work as not usable in deviation statistics
B035	R2	Duan, Luebke & Pennline, Int. J. Clean Coal Energy 1, 1 (2012)	dH_rxn(300 K)	-176.751	-178.45	-1.70	1.70	IND-DFT	yes	
B036	R2	Duan, Luebke & Pennline, Int. J. Clean Coal Energy 1, 1 (2012)	dG_rxn(300 K)	-129.523	-130.37	-0.85	0.85	IND-DFT	yes	
B037	R2	HSC Chemistry / FactSage database, as tabulated by Duan et al. (2012)	dH_rxn(300 K)	-178.166	-178.45	-0.28	0.28	SHARED-LIN	no	flagged in this work as not usable in deviation statistics
B038	R2	HSC Chemistry / FactSage database, as tabulated by Duan et al. (2012)	dG_rxn(300 K)	-130.127	-130.37	-0.25	0.25	SHARED-LIN	no	flagged in this work as not usable in deviation statistics
B039	R3	Duan, Luebke & Pennline, Int. J. Clean Coal Energy 1, 1 (2012)	dH_rxn(300 K)	-80.212	-69.13	11.08	11.08	IND-DFT	yes	
B040	R3	Duan, Luebke & Pennline, Int. J. Clean Coal Energy 1, 1 (2012)	dG_rxn(300 K)	-76.610	-64.13	12.48	12.48	IND-DFT	yes	
B041	R3	HSC Chemistry / FactSage database, as tabulated by Duan et al. (2012)	dH_rxn(300 K)	-69.035	-69.13	-0.09	0.09	SHARED-LIN	no	flagged in this work as not usable in deviation statistics
B042	R3	HSC Chemistry / FactSage database, as tabulated by Duan et al. (2012)	dG_rxn(300 K)	-64.033	-64.13	-0.10	0.10	SHARED-LIN	no	flagged in this work as not usable in deviation statistics
B043	R9	Duan, Luebke & Pennline, Int. J. Clean Coal Energy 1, 1 (2012)	dH_rxn(300 K)	-32.687	-35.50	-2.82	2.82	IND-DFT	yes	
B044	R9	Duan, Luebke & Pennline, Int. J. Clean Coal Energy 1, 1 (2012)	dG_rxn(300 K)	-26.177	-28.58	-2.40	2.40	IND-DFT	yes	
B045	R9	HSC Chemistry / FactSage database, as tabulated by Duan et al. (2012)	dH_rxn(300 K)	-19.665	-35.50	-15.84	15.84	SHARED-LIN	no	flagged in this work as not usable in deviation statistics
B046	R9	HSC Chemistry / FactSage database, as tabulated by Duan et al. (2012)	dG_rxn(300 K)	-12.762	-28.58	-15.81	15.81	SHARED-LIN	no	flagged in this work as not usable in deviation statistics
B047	R16	Duan, Luebke & Pennline, Int. J. Clean Coal Energy 1, 1 (2012)	reaction not in the v2 set	-153.824	–	–	–	IND-DFT	no	R16, the Na2CO3/NaHCO3 route, is outside the 23-reaction set, so no computed value exists for comparison; the row is retained in the register for its recorded published value.

continued on the next page

Table S3.1 (continued)

Row	Rxn	Source	Quantity	Published	Computed	Dev.	|Dev.|	Class	Used	Note / reason not used
B048	R16	Duan, Luebke & Pennline, Int. J. Clean Coal Energy 1, 1 (2012)	reaction not in the v2 set	-58.739	–	–	–	IND-DFT	no	R16, the Na2CO3/NaHCO3 route, is outside the 23-reaction set, so no computed value exists for comparison; the row is retained in the register for its recorded published value.
B049	R16	HSC Chemistry / FactSage database, as tabulated by Duan et al. (2012)	reaction not in the v2 set	-135.341	–	–	–	SHARED-LIN	no	flagged in this work as not usable in deviation statistics
B050	R16	HSC Chemistry / FactSage database, as tabulated by Duan et al. (2012)	reaction not in the v2 set	-34.126	–	–	–	SHARED-LIN	no	flagged in this work as not usable in deviation statistics
B051	R9	Duan, Luebke & Pennline, Int. J. Clean Coal Energy 1, 1 (2012)	turnover temperature at P_CO2 = 0.1 bar	600	790.9	190.9	190.9	IND-DFT	no	flagged in this work as not usable in deviation statistics
B052	X	Duan, Luebke & Pennline, Int. J. Clean Coal Energy 1, 1 (2012)	reaction not in the v2 set	460	–	–	–	IND-DFT	no	flagged in this work as not usable in deviation statistics
B053	X	Duan, Luebke & Pennline, Int. J. Clean Coal Energy 1, 1 (2012)	reaction not in the v2 set	520	–	–	–	IND-DFT	no	flagged in this work as not usable in deviation statistics
B054	R2	Lackner et al., Energy 20, 1153 (1995)	turnover temperature at P_CO2 = 1 bar	1161	1159.0	-2.0	2.0	SHARED-LIN	yes	
B055	R2	Lackner et al., Energy 20, 1153 (1995)	dH_rxn at the source's own T_max = 1670 K	-167	-155.38	11.62	11.62	SHARED-LIN	no	flagged in this work as not usable in deviation statistics
B056	R1	Lackner et al., Energy 20, 1153 (1995)	turnover temperature at P_CO2 = 1 bar	680	669.5	-10.5	10.5	SHARED-LIN	yes	
B057	R1	Lackner et al., Energy 20, 1153 (1995)	dH_rxn at the source's own T_max = 930 K	-115	-107.63	7.37	7.37	SHARED-LIN	no	flagged in this work as not usable in deviation statistics
B058	R3	Lackner et al., Energy 20, 1153 (1995)	turnover temperature at P_CO2 = 1 bar	1161	–	–	–	SHARED-LIN	no	Observation, not a correction: Lackner et al. 1995 Table 2 gives Ca(OH)2 the same T_max = 1161 K as CaO (row B054), the same pattern documented for brucite (OV01) and chrysotile (OV02), in which the hydroxide is assigned the anhydrous oxide's value because it dehydrates before it carbonates. The row produces no comparison in any case: the v2 model finds no turnover root for R3 in 300-2000 K at P_CO2 = 1 bar, because dG0(R3) rises only weakly with temperature (dS0_rxn = -16.6 J/(mol K)).
B059	R3	Lackner et al., Energy 20, 1153 (1995)	dH_rxn at the source's own T_max = 1161 K	-68	-75.71	-7.71	7.71	SHARED-LIN	no	flagged in this work as not usable in deviation statistics
B060	R9	Lackner et al., Energy 20, 1153 (1995)	turnover temperature at P_CO2 = 1 bar	680	1315.6[H]	635.6	635.6	SHARED-LIN	no	excluded by override OV01 (see benchmark_overrides.csv)

continued on the next page

Table S3.1 (continued)

Row	Rxn	Source	Quantity	Published	Computed	Dev.	|Dev.|	Class	Used	Note / reason not used
B061	R9	Lackner et al., Energy 20, 1153 (1995)	dH_rxn at the source's own T_max = 680 K	-37	-38.31	-1.31	1.31	SHARED-LIN	no	flagged in this work as not usable in deviation statistics
B062	R4	Lackner et al., Energy 20, 1153 (1995)	turnover temperature at P_CO2 = 1 bar	554	552.9	-1.1	1.1	SHARED-LIN	yes	
B063	R4	Lackner et al., Energy 20, 1153 (1995)	dH_rxn at the source's own T_max = 554 K	-87	-86.57	0.43	0.43	SHARED-LIN	no	flagged in this work as not usable in deviation statistics
B064	R10	Lackner et al., Energy 20, 1153 (1995)	turnover temperature at P_CO2 = 1 bar	474	480.6	6.6	6.6	SHARED-LIN	yes	
B065	R10	Lackner et al., Energy 20, 1153 (1995)	dH_rxn at the source's own T_max = 474 K	-81	-82.58	-1.58	1.58	SHARED-LIN	no	flagged in this work as not usable in deviation statistics
B066	R5	Lackner et al., Energy 20, 1153 (1995)	turnover temperature at P_CO2 = 1 bar	515	495.7	-19.3	19.3	SHARED-LIN	yes	
B067	R5	Lackner et al., Energy 20, 1153 (1995)	dH_rxn at the source's own T_max = 515 K	-88	-85.11	2.90	2.90	SHARED-LIN	no	flagged in this work as not usable in deviation statistics
B068	R13	Lackner et al., Energy 20, 1153 (1995)	turnover temperature at P_CO2 = 1 bar	437	459.4	22.4	22.4	SHARED-LIN	yes	
B069	R13	Lackner et al., Energy 20, 1153 (1995)	dH_rxn at the source's own T_max = 437 K	-71	-74.85	-3.85	3.85	SHARED-LIN	no	flagged in this work as not usable in deviation statistics
B070	R14	Lackner et al., Energy 20, 1153 (1995)	turnover temperature at P_CO2 = 1 bar	438	426.1	-11.9	11.9	SHARED-LIN	yes	
B071	R14	Lackner et al., Energy 20, 1153 (1995)	dH_rxn at the source's own T_max = 438 K	-81	-82.85	-1.85	1.85	SHARED-LIN	no	flagged in this work as not usable in deviation statistics
B072	R11	Lackner et al., Energy 20, 1153 (1995)	turnover temperature at P_CO2 = 1 bar	680	481.0[L]	-199.0	199.0	SHARED-LIN	no	excluded by override OV02 (see benchmark_overrides.csv)
B073	R11	Lackner et al., Energy 20, 1153 (1995)	dH_rxn at the source's own T_max = 680 K	-35	-32.89	2.11	2.11	SHARED-LIN	no	flagged in this work as not usable in deviation statistics
B074	R19	Lackner et al., Energy 20, 1153 (1995) [grossular]	turnover temperature at P_CO2 = 1 bar	465	470.0	5.0	5.0	SHARED-LIN	yes	
B075	X	Lackner et al., Energy 20, 1153 (1995) [anorthite glass]	reaction not in the v2 set	691	–	–	–	SHARED-LIN	no	flagged in this work as not usable in deviation statistics
B076	R20	Lackner et al., Energy 20, 1153 (1995) [pyrope]	turnover temperature at P_CO2 = 1 bar	533	522.4	-10.6	10.6	SHARED-LIN	yes	
B077	R2	Lackner et al., Energy 20, 1153 (1995)	turnover temperature at P_CO2 = 200 bar	1670	1691.7[H]	21.7	21.7	SHARED-LIN	no	flagged in this work as not usable in deviation statistics
B078	R1	Lackner et al., Energy 20, 1153 (1995)	turnover temperature at P_CO2 = 200 bar	930	911.7	-18.3	18.3	SHARED-LIN	no	flagged in this work as not usable in deviation statistics
B079	R1	Robie & Hemingway, USGS Bull. 2131 (1995)	turnover temperature at P_CO2 = 1 bar	679	669.5	-9.5	9.5	SHARED-LIN	yes	
B080	R1	Robie & Hemingway, USGS Bull. 2131 (1995)	dG_rxn(298.15 K)	-65.8	-64.20	1.60	1.60	SHARED-LIN	yes	
B081	R2	Robie & Hemingway, USGS Bull. 2131 (1995)	turnover temperature at P_CO2 = 1 bar	1161	1159.0	-2.0	2.0	SHARED-LIN	yes	
B082	R2	Robie & Hemingway, USGS Bull. 2131 (1995)	dG_rxn(298.15 K)	-131.0	-130.67	0.33	0.33	SHARED-LIN	yes	
B083	R3	Robie & Hemingway, USGS Bull. 2131 (1995)	turnover temperature at P_CO2 = 1 bar	>700	–	–	–	SHARED-LIN	no	flagged in this work as not usable in deviation statistics

continued on the next page

Table S3.1 (continued)

Row	Rxn	Source	Quantity	Published	Computed	Dev.	|Dev.|	Class	Used	Note / reason not used
B084	R3	Robie & Hemingway, USGS Bull. 2131 (1995)	dG_rxn(298.15 K)	-64.7	-64.17	0.54	0.54	SHARED-LIN	yes	
B085	R4	Robie & Hemingway, USGS Bull. 2131 (1995)	turnover temperature at P_CO2 = 1 bar	558	552.9	-5.1	5.1	SHARED-LIN	yes	
B086	R4	Robie & Hemingway, USGS Bull. 2131 (1995)	dG_rxn(298.15 K)	-41.4	-40.68	0.72	0.72	SHARED-LIN	yes	
B087	R5	Robie & Hemingway, USGS Bull. 2131 (1995)	turnover temperature at P_CO2 = 1 bar	508	495.7	-12.3	12.3	SHARED-LIN	yes	
B088	R5	Robie & Hemingway, USGS Bull. 2131 (1995)	dG_rxn(298.15 K)	-36.5	-34.34	2.16	2.16	SHARED-LIN	yes	
B089	R9	Robie & Hemingway, USGS Bull. 2131 (1995)	turnover temperature at P_CO2 = 1 bar	>800	1315.6[H]	–	–	SHARED-LIN	no	flagged in this work as not usable in deviation statistics
B090	R9	Robie & Hemingway, USGS Bull. 2131 (1995)	dG_rxn(298.15 K)	-30.2	-28.62	1.58	1.58	SHARED-LIN	yes	
B091	R10	Robie & Hemingway, USGS Bull. 2131 (1995)	turnover temperature at P_CO2 = 1 bar	491	480.6	-10.4	10.4	SHARED-LIN	yes	
B092	R10	Robie & Hemingway, USGS Bull. 2131 (1995)	dG_rxn(298.15 K)	-33.1	-31.54	1.56	1.56	SHARED-LIN	yes	
B093	R11	Robie & Hemingway, USGS Bull. 2131 (1995)	turnover temperature at P_CO2 = 1 bar	>800	481.0[L]	–	–	SHARED-LIN	no	Inconsistent with the cited source and retained at use=no. R11 has a NEGATIVE reaction entropy (-68.9 J/(mol K): one mole of CO2 is replaced by two thirds of a mole of H2O(g)), so dG_r0 RISES with temperature and must cross zero at LOW temperature, not above 800 K. Recomputing from Robie & Hemingway's own 298 K free energies gives dG_r0(298) = -14.2 kJ/mol and a crossing near 490 K.
B094	R11	Robie & Hemingway, USGS Bull. 2131 (1995)	dG_rxn(298.15 K)	-14.9	-12.63	2.27	2.27	SHARED-LIN	yes	
B095	R12	Robie & Hemingway, USGS Bull. 2131 (1995)	turnover temperature at P_CO2 = 1 bar	713	710.0	-3.0	3.0	SHARED-LIN	yes	
B096	R12	Robie & Hemingway, USGS Bull. 2131 (1995)	dG_rxn(298.15 K)	-66.7	-66.14	0.56	0.56	SHARED-LIN	yes	
B097	R13	Robie & Hemingway, USGS Bull. 2131 (1995)	turnover temperature at P_CO2 = 1 bar	466	459.4	-6.6	6.6	SHARED-LIN	yes	
B098	R13	Robie & Hemingway, USGS Bull. 2131 (1995)	dG_rxn(298.15 K)	-27.5	-26.45	1.05	1.05	SHARED-LIN	yes	
B099	R14	Robie & Hemingway, USGS Bull. 2131 (1995)	turnover temperature at P_CO2 = 1 bar	428	426.1	-1.9	1.9	SHARED-LIN	yes	
B100	R14	Robie & Hemingway, USGS Bull. 2131 (1995)	dG_rxn(298.15 K)	-25.6	-25.11	0.49	0.49	SHARED-LIN	yes	
B101	R15	Robie & Hemingway, USGS Bull. 2131 (1995)	turnover temperature at P_CO2 = 1 bar	692	694.5	2.5	2.5	SHARED-LIN	yes	
B102	R15	Robie & Hemingway, USGS Bull. 2131 (1995)	dG_rxn(298.15 K)	-69.1	-69.63	-0.53	0.53	SHARED-LIN	yes	

continued on the next page

Table S3.1 (continued)

Row	Rxn	Source	Quantity	Published	Computed	Dev.	|Dev.|	Class	Used	Note / reason not used
B103	R2	Harker & Tuttle, Am. J. Sci. 253, 209 (1955)	turnover temperature at P_CO2 = 6.9 bar	1253-1283	1305.6^{H}	22.6	22.6	IND-EXP	yes	
B104	R2	Harker & Tuttle, Am. J. Sci. 253, 209 (1955)	turnover temperature at P_CO2 = 20.7 bar	1303-1343	1408.5^{H}	65.5	65.5	IND-EXP	yes	
B105	R2	Harker & Tuttle, Am. J. Sci. 253, 209 (1955)	turnover temperature at P_CO2 = 34.5 bar	1373-1393	1462.8^{H}	69.8	69.8	IND-EXP	yes	
B106	R1	Harker & Tuttle, Am. J. Sci. 253, 209 (1955)	turnover temperature at P_CO2 = 68.9 bar	1003-1023	848.7	-154.3	154.3	IND-EXP	no	flagged in this work as not usable in deviation statistics
B107	R1	Harker & Tuttle, Am. J. Sci. 253, 209 (1955)	turnover temperature at P_CO2 = 31 bar	>923	807.3	–	–	IND-EXP	no	flagged in this work as not usable in deviation statistics
B108	R4	Harker & Tuttle, Am. J. Sci. 254, 239 (1956)	turnover temperature at P_CO2 = 1034 bar	913-963	889.4	-23.6	23.6	IND-EXP	no	flagged in this work as not usable in deviation statistics
B109	R7	Nakagawa & Ohashi, J. Electrochem. Soc. 145, 1344 (1998)	turnover temperature at P_CO2 = 1 bar	723-823	1076.5	253.5	253.5	IND-EXP	no	excluded by override OV08 (see benchmark_overrides.csv)
B110	R7	Nakagawa & Ohashi, J. Electrochem. Soc. 145, 1344 (1998)	turnover temperature at P_CO2 = 1 bar	>923	1076.5	–	–	IND-EXP	no	One-sided experimental constraint on the R7 turnover rather than a point value, so it is scored as satisfied or violated and cannot enter a deviation statistic.
B111	R7	Kato & Nakagawa, J. Ceram. Soc. Jpn. 109, 911 (2001)	turnover temperature at P_CO2 = 1 bar	723-823	1076.5	253.5	253.5	SHARED-LIN	no	flagged in this work as not usable in deviation statistics
B112	R7	Kato & Nakagawa, J. Ceram. Soc. Jpn. 109, 911 (2001)	turnover temperature at P_CO2 = 1 bar	>923	1076.5	–	–	SHARED-LIN	no	flagged in this work as not usable in deviation statistics

Table S3.2 documents the comparison rules, source classifications and qualifications used to match each literature datum to its computed counterpart. Source-register and comparison identifiers are retained so that exclusions, one-sided constraints and shared-data records can be traced individually.

Table S3.2 Comparison rules and source qualifications. Source-register fields and the applied comparison treatment are shown for each rule, with the reason and source. These rules concern literature matching and inclusion, not changes in the manuscript results.

ID	Record	Field	Source register	Comparison treatment	Reason	Source
OV01	B060	use_in_deviation_stats	yes	no	Lackner et al. 1995 Table 2 gives brucite the same T_max = 680 K as periclase and lists a separate dehydration temperature T_deh = 538 K; the 680 K is the MgO value quoted because brucite dehydrates before it carbonates. It is not a turnover temperature for $Mg(OH)_2 + CO_2$ -> $MgCO_3 + H_2O(g)$.	This work, provenance analysis; ESI section S6
OV02	B072	use_in_deviation_stats	yes	no	The analogous source convention applies to chrysotile; the source note on that row already records 'source estimates T_max from the MgO value, see p. 1161'.	This work, provenance analysis; the source note on row B072
OV03	B074	reaction_id	X	R19	Lackner et al. 1995 Table 2 grossular, T_max = 465 K at 1 bar. Grossular ($Ca_3Al_2Si_3O_{12}$) is in the species set of this work, so the row is not out-of-set.	This work, provenance analysis
OV04	B074	use_in_deviation_stats	no	yes	Included using the reaction mapping in OV03.	This work, provenance analysis
OV05	B076	reaction_id	X	R20	Lackner et al. 1995 Table 2 pyrope, T_max = 533 K at 1 bar. Pyrope ($Mg_3Al_2Si_3O_{12}$) is in the species set of this work.	This work, provenance analysis
OV06	B076	use_in_deviation_stats	no	yes	Included using the reaction mapping in OV05.	This work, provenance analysis
OV07	B093	note_this_work	–	note [a] below	The published row is inconsistent with its cited source on the sign of the temperature dependence; the recomputation is recorded in the note and the row is retained at use=no.	This work, provenance analysis; ESI section S6
OV08	B109	use_in_deviation_stats	yes	no	Nakagawa & Ohashi 1998 report a window of PROMPT CO_2 UPTAKE (723-823 K), which is a kinetic operating window chosen for rate, not the equilibrium turnover temperature. The equilibrium-relevant datum from the same experiment is the reversal (regeneration) onset, B110, which is retained as a one-sided constraint. This kinetic-window row is excluded from the equilibrium deviation statistics.	This work, atlas-construction decision
OV09	source_key == Lackner1995	independence_class	INDEPENDENT	SHARED-DATA-LINEAGE	Lackner et al. 1995 Table 2 states it is based on their ref. 25 = Robie, Hemingway & Fisher, USGS Bulletin 1452 (1978), the earlier edition that USGS Bulletin 2131 supersedes, and Bulletin 2131 supplies the 298 K reference frame of this work for 15 of 32 species. Agreement with these rows is an internal-consistency (structural) check, not independent accuracy.	This work, data-lineage analysis (this work, Section 5.8)
OV10	source_key == RobieHemingway1995	independence_class	INDEPENDENT	SHARED-DATA-LINEAGE	Same lineage as OV09, and the direct source of the 298 K anchors for the silicate and aluminosilicate species. Rows derived from it cannot test the accuracy of data taken from it.	This work, data-lineage analysis (this work, Section 5.8)

ID	Record	Field	Source register	Comparison treatment	Reason	Source
OV11	source_key == KatoNakagawa2001	independence_class	SHARED-DATA-LINEAGE	SHARED-DATA-LINEAGE	Source classification retained for completeness. Same Toshiba group and apparatus as NakagawaOhashi1998, reporting identical numbers; treated as a shared record in the comparison register.	This work, data-lineage analysis
OV12	source_key == HSC	independence_class	SHARED-DATA-LINEAGE	SHARED-DATA-LINEAGE	Source classification retained for completeness. HSC/FactSage assessed values share the critically assessed lineage of the model inputs; this is demonstrated directly for magnesite (HSC's dH(R1) sits within 0.2 kJ/mol of the older NBS/Wagman 1982 anchor).	This work, data-lineage analysis
OV13	B058	note_this_work	–	note [b] below	Recorded as an observation on the source's treatment of hydroxides; no value is changed and the row produces no comparison in any case.	This work, observation

[a] The value set by OV07 on `B093`, field `note_this_work`, in full: Inconsistent with the cited source and retained at use=no. R11 has a NEGATIVE reaction entropy (-68.9 J/(mol K): one mole of CO_2 is replaced by two thirds of a mole of H_2O(g)), so dG_r0 RISES with temperature and must cross zero at LOW temperature, not above 800 K. Recomputing from Robie & Hemingway's own 298 K free energies gives dG_r0(298) = -14.2 kJ/mol and a crossing near 490 K. The exclusion rests on that entropy-sign argument alone.

[b] The value set by OV13 on `B058`, field `note_this_work`, in full: Observation, not a correction: Lackner et al. 1995 Table 2 gives $Ca(OH)_2$ the same T_max = 1161 K as CaO (row B054), the same pattern documented for brucite (OV01) and chrysotile (OV02), in which the hydroxide is assigned the anhydrous oxide's value because it dehydrates before it carbonates. The row produces no comparison in any case: no turnover root is found for R3 in 300-2000 K at P_CO_2 = 1 bar, because dG0(R3) rises only weakly with temperature (dS0_rxn = -16.6 J/(mol K)).

Table S3.3 Deviation statistics by benchmark class. The counts in the last two columns are the number of comparisons within 25 and 50 K. The medians describe the stated comparison sets, with their phase, pressure and validity limitations. Shared-data agreement checks consistency with the source thermochemistry; it is not independent evidence of physical accuracy.

Benchmark class	n	median	mean	max	≤25 K	≤50 K
(a) turnover temperature, $\lvert\Delta T_{turn}\rvert$ / K						
Independent, first principles	11	71.7	132.7	675.4	3/11	3/11
Independent, experimental	3	65.5	52.6	69.8	1/3	1/3
Secondary source	1	36.0	36.0	36.0	0/1	1/1
Shared data lineage	18	6.6	7.9	22.4	18/18	18/18
**Independent, both together**	14	**67.7**	115.5	675.4	4/14	4/14
(b) standard reaction properties, $\lvert\Delta\rvert$ / kJ mol^{-1}						
Independent, first principles	14	4.27	5.11	12.48	–	–
Shared data lineage	12	0.89	1.12	2.27	–	–

Ten comparisons deviate by more than 50 K: B001, B002, B003, B008, B013, B014, B020, B024, B104 and B105. Carbonate melting above 993 K for Li_2CO_3 and 1123 K for Na_2CO_3 contributes to the four alkali-ceramic rows B013, B014, B020 and B024, the first of which is the 675.4 K R7 outlier. The magnesite reference state contributes to the three magnesium rows B001, B002 and B008. The remaining three, B003, B104 and B105, are all CaO comparisons whose computed value lies above the common heat-capacity interval. B008 has a second and more specific explanation: the published 600 K lies above the brucite dehydroxylation temperature of 537.6 K computed in Section 5.6 of the article, so the two sides are not comparing the same reactant. These contributions are not exclusive explanations of the residual differences and do not establish that independent first-principles results are incorrect.

Two additional magnesite comparisons help qualify the reference state. Koziol and Newton's reversal at 715°C and 590 ± 30 bar is close to the ideal-gas value, 987.2 versus 988.15 K, but cannot establish 1 K predictive accuracy. Their tabulated 988 K, 600 bar point has $f_{CO_2} = 682$ bar: the carbonation fugacity correction is approximately -1.05 kJ mol^{-1} and the condensed-phase pressure contribution is $+1.03$ kJ mol^{-1}. Their near cancellation explains why a close ideal-gas comparison can occur. This is not a validation of the 20 bar fugacity sensitivity or of diluted feeds. Bénézeth et al. report $\Delta_f H^\circ(298.15) = -1111.75 \pm 2$ and $\Delta_f G^\circ(298.15) = -1026.48 \pm 2$ kJ mol^{-1} with a stated 3σ uncertainty. Their enthalpy agrees with the adopted anchor to 0.06 kJ mol^{-1}. The adopted enthalpy and entropy give -1027.90 kJ mol^{-1} for the Gibbs energy, differing by 1.42 kJ mol^{-1} and lying within their interval. The difference is carried by the entropy, their 60.00 against the adopted measured 65.09 J mol^{-1} K^{-1}, not by the enthalpy. Combining their measured solubility product with CODATA ion values instead gives -1027.79 kJ mol^{-1}, within 0.10 kJ mol^{-1} of the present value; the two routes are different comparisons and both are reported. Source details are given in Section S7.3.

One lineage attribution follows from this. The only rows in the atlas traced to the NBS magnesite anchor are the assessed-database columns covered by override OV12 of Table S3.2; separately calculated first-principles columns are not described as sharing it, and Section 5.8 of the article is worded accordingly.

Twenty-six atlas rows concern a Class II or Class III reaction. Of the eight that survive into the deviation statistics, seven are standard-state enthalpies and Gibbs energies, for which no water convention arises. The eighth, B008, is the only hydrous turnover comparison in the statistics: $Mg(OH)_2$ at $P_{CO_2} = 0.1$ bar, published 600 K against 790.9 K computed. Its source locator and the value computed here both use $p_{H_2O} = 1$ bar, so the two sides are matched on the water convention. The residual is nevertheless most simply read as a phase-identity difference: brucite dehydroxylates at 537.6 K at that water pressure, below both numbers, so neither side describes a stable hydroxide reactant (Section 5.6 of the article). Table S3.1 records this matched convention in the note column for B005–B008 and B051.

S4. Numerical cross-check against an element-potential minimization

For each restricted species set used here, the element-composition matrix has nullity one. Its feasible composition space is therefore the stated reaction coordinate, subject to nonnegative mole numbers. Minimizing over these species and minimizing along that coordinate are mathematically equivalent. This equivalence does not establish global equilibrium against unlisted phases, alternative reactions, solid solutions or condensed water.

An element-potential (RAND) minimization was therefore formulated independently from the published algorithm of White, Johnson and Dantzig (*J. Chem. Phys.*, 1958, **28**, 751) in the form given by Smith and Missen (*Chemical Reaction Equilibrium Analysis*, Wiley, 1982), minimizing

$$G/RT = \sum_{i\in\text{gas}} n_i \left[c_i + \ln(n_i/n_{\text{gas}}) + \ln(P/P^\circ)\right] + \sum_{j\in\text{cond}} n_j c_j$$

subject to $\sum_i a_{ki} n_i = b_k$ and $n_i \geq 0$, with $c_i = g^\circ_{i,\text{ref}}(T)/(RT)$ taken from the same Shomate database used throughout this work. Here a_{ki} is the number of atoms of element k in one mole of species i, and b_k is the total number of moles of element k in the charge, which the reaction conserves. Introducing a multiplier λ_k for each element balance and setting the derivatives of the Lagrangian to zero gives the stationarity conditions $c_i + \ln(n_i/n_{\text{gas}}) + \ln(P/P^\circ) = \sum_k \lambda_k a_{ki}$ for gas species and $c_j = \sum_k \lambda_k a_{kj}$ for every condensed phase present. The λ_k are the element potentials, and it is from the converged λ_k that $\ln P_{CO_2}$ is read in Table S4.2. The second formulation treats every species mole number as an unknown, imposes the element balances through Lagrange multipliers, and determines which of the admitted condensed phases are present by enumeration. It is given the element balances, not the reaction, and it shares no numerical machinery with the ξ formalism.

Because the two formulations draw on the same thermochemical database, the comparison tests the numerical consistency of the two implementations and the completeness of the one-dimensional reaction-coordinate parameterization for the systems examined. It is not a test of the accuracy of the thermochemical data, which is the separate question addressed by Section S3.

Table S4.1 Agreement between the extent-of-reaction formalism, the independently formulated element-potential minimization and the affinity-based reconstruction, summarized over the whole state grid. These maxima are the source of the verification figures quoted in Section 5.8 of the article.

Quantity	Achieved	Gate	Verdict
Maximum $\lvert\Delta\xi_{\text{eq}}\rvert$ over 368 states (88 with an interior minimum)	4.443×10^{-7}	1×10^{-6}	PASS
Maximum $\lvert\Delta T_{\text{turn}}\rvert$ over 89 turnover pairs / K	2.135×10^{-9}	1 K	PASS
Maximum $\lvert\Delta\xi_{\text{eq}}\rvert$ of the affinity reconstruction over the 1495-state grid	2.162×10^{-7}	1×10^{-6}	PASS
Maximum $\lvert\Delta\xi_{\text{eq}}\rvert$ of the affinity reconstruction over the 368 verification states	1.604×10^{-7}	1×10^{-6}	PASS
Maximum atom-balance residual of the element-potential solver / mol	1.776×10^{-15}	–	–
Maximum residual on fitting the returned composition back to $n_i = n_i^0 + \nu_i\xi$ / mol	3.331×10^{-16}	–	–
Maximum $\lvert\Delta G\rvert$ at equilibrium / kJ	4.391×10^{-6}	–	–
Phase-selection disagreements	0	0	PASS
Turnover combinations with no root by either method	3 of 92	agree	PASS

Two structural properties of the formalism follow from the same comparison. The element-potential solution, obtained with no knowledge of the reaction stoichiometry, lies on the reaction line $n_i = n_i^0 + \nu_i\xi$ to within 3.3×10^{-16} mol, so the one-dimensional ξ parameterization spans each restricted species set; and the condensed-phase set selected independently by the second formulation matches the one implied by ξ_{eq} in every state. The maximum residual 4.44×10^{-7} in ξ_{eq} is within the stated 10^{-6} numerical tolerance and is smaller than the displayed precision of percentage conversions in the article. A third check, an affinity-based reconstruction that resolves the stationarity condition of eqn (7) of the article independently at every state, reproduces the 1495-state sweep to 2.16×10^{-7} in extent, the maximum occurring for R8 at 900 K and 1 bar, and the 368-state verification grid to 1.60×10^{-7}. All three maxima are listed in Table S4.1, which is the locus for the corresponding figures quoted in Section 5.8 of the article.

The numerical verification grid contains 368 states, 16 per reaction: $T = 500$, 700, 900, 1100 and 1300 K at 1 bar for each of $n_{N_2,0} = 0$ and 1 mol; $T = 600$, 800, 1000 and 1200 K at 1 bar with 4 mol inert; and $T = 700$ and 1000 K at 20 bar with 4 mol inert. The 1 mol CO_2 and water conventions are those of the reaction-coordinate calculation. Temperatures outside a reaction's data window still test numerical equivalence, but not physical extrapolation. The full 368-state numerical outputs are supplied in `Table_S4_3_equilibrium_crosscheck.csv`; the 92 buffered turnover checks are in `Table_S4_crosscheck.csv`. The runtime versions are stated in Section S7.5.

Table S4.2 Turnover temperature from the extent-of-reaction formalism and from the independent element-potential minimization, for every reaction and every CO_2 partial pressure. The element-potential route reads $\ln P_{CO_2}$ off the converged Lagrange multipliers of a buffered two-solid coexistence and then root-finds; it is told the element balances, not the reaction. Hydrous rows use $p_{H_2O} = 1$ bar, as in Table S2.4. This is a numerical consistency check, including extrapolated roots, not a validation of the thermochemical inputs.

Rxn	Reaction	P_{CO_2} / bar	T_{turn} / K extent	T_{turn} / K element pot.	$\lvert\Delta T\rvert$ / K	Status
R1	$MgO + CO_2 \rightarrow MgCO_3$	0.10	601.6551	601.6551	2.82×10^{-10}	agree
R1	$MgO + CO_2 \rightarrow MgCO_3$	0.15	612.5427	612.5427	1.25×10^{-11}	agree
R1	$MgO + CO_2 \rightarrow MgCO_3$	1.00	669.4788	669.4788	6.31×10^{-11}	agree
R1	$MgO + CO_2 \rightarrow MgCO_3$	20.00	786.4312	786.4312	3.64×10^{-12}	agree
R2	$CaO + CO_2 \rightarrow CaCO_3$	0.10	1024.0683	1024.0683	3.64×10^{-12}	agree
R2	$CaO + CO_2 \rightarrow CaCO_3$	0.15	1045.3781	1045.3781	2.27×10^{-13}	agree
R2	$CaO + CO_2 \rightarrow CaCO_3$	1.00	1158.9905	1158.9905	1.00×10^{-11}	agree
R2	$CaO + CO_2 \rightarrow CaCO_3$	20.00	1405.0498	1405.0498	4.14×10^{-10}	agree
R3	$Ca(OH)_2 + CO_2 \rightarrow CaCO_3 + H_2O(g)$	0.10	1633.3682	1633.3682	4.77×10^{-12}	agree
R3	$Ca(OH)_2 + CO_2 \rightarrow CaCO_3 + H_2O(g)$	0.15	1775.9437	1775.9437	4.55×10^{-13}	agree
R3	$Ca(OH)_2 + CO_2 \rightarrow CaCO_3 + H_2O(g)$	1.00	–	–	–	no root in 300-2000 K (both methods)
R3	$Ca(OH)_2 + CO_2 \rightarrow CaCO_3 + H_2O(g)$	20.00	–	–	–	no root in 300-2000 K (both methods)
R4	$CaSiO_3 + CO_2 \rightarrow CaCO_3 + SiO_2$	0.10	492.9212	492.9212	8.53×10^{-13}	agree
R4	$CaSiO_3 + CO_2 \rightarrow CaCO_3 + SiO_2$	0.15	502.4867	502.4867	1.93×10^{-9}	agree
R4	$CaSiO_3 + CO_2 \rightarrow CaCO_3 + SiO_2$	1.00	552.8995	552.8995	1.38×10^{-9}	agree
R4	$CaSiO_3 + CO_2 \rightarrow CaCO_3 + SiO_2$	20.00	658.4615	658.4615	7.28×10^{-12}	agree
R5	$\frac{1}{2}Mg_2SiO_4 + CO_2 \rightarrow MgCO_3 + \frac{1}{2}SiO_2$	0.10	446.2025	446.2025	3.58×10^{-12}	agree
R5	$\frac{1}{2}Mg_2SiO_4 + CO_2 \rightarrow MgCO_3 + \frac{1}{2}SiO_2$	0.15	454.1684	454.1684	1.21×10^{-11}	agree
R5	$\frac{1}{2}Mg_2SiO_4 + CO_2 \rightarrow MgCO_3 + \frac{1}{2}SiO_2$	1.00	495.6809	495.6809	2.39×10^{-12}	agree
R5	$\frac{1}{2}Mg_2SiO_4 + CO_2 \rightarrow MgCO_3 + \frac{1}{2}SiO_2$	20.00	580.0945	580.0945	1.76×10^{-11}	agree
R6	$Li_4SiO_4 + CO_2 \rightarrow Li_2CO_3 + Li_2SiO_3$	0.10	794.7574	794.7574	2.61×10^{-12}	agree
R6	$Li_4SiO_4 + CO_2 \rightarrow Li_2CO_3 + Li_2SiO_3$	0.15	810.8461	810.8461	1.02×10^{-12}	agree
R6	$Li_4SiO_4 + CO_2 \rightarrow Li_2CO_3 + Li_2SiO_3$	1.00	895.9265	895.9265	4.55×10^{-12}	agree
R6	$Li_4SiO_4 + CO_2 \rightarrow Li_2CO_3 + Li_2SiO_3$	20.00	1121.2876	1121.2876	2.66×10^{-11}	agree
R7	$Li_2ZrO_3 + CO_2 \rightarrow Li_2CO_3 + ZrO_2$	0.10	916.7924	916.7924	1.31×10^{-11}	agree
R7	$Li_2ZrO_3 + CO_2 \rightarrow Li_2CO_3 + ZrO_2$	0.15	936.9183	936.9183	4.55×10^{-13}	agree
R7	$Li_2ZrO_3 + CO_2 \rightarrow Li_2CO_3 + ZrO_2$	1.00	1076.4582	1076.4582	6.82×10^{-13}	agree
R7	$Li_2ZrO_3 + CO_2 \rightarrow Li_2CO_3 + ZrO_2$	20.00	1675.3731	1675.3731	2.50×10^{-11}	agree
R8	$Na_2ZrO_3 + CO_2 \rightarrow Na_2CO_3 + ZrO_2$	0.10	903.1400	903.1400	6.07×10^{-10}	agree
R8	$Na_2ZrO_3 + CO_2 \rightarrow Na_2CO_3 + ZrO_2$	0.15	923.2357	923.2357	7.50×10^{-12}	agree

continued on the next page

Rxn	Reaction	P_{CO_2} / bar	T_{turn} / K extent	T_{turn} / K element pot.	$\lvert\Delta T\rvert$ / K	Status
R8	$Na_2ZrO_3 + CO_2 \rightarrow Na_2CO_3 + ZrO_2$	1.00	1032.1623	1032.1623	6.82×10^{-12}	agree
R8	$Na_2ZrO_3 + CO_2 \rightarrow Na_2CO_3 + ZrO_2$	20.00	1346.7165	1346.7165	2.05×10^{-9}	agree
R9	$Mg(OH)_2 + CO_2 \rightarrow MgCO_3 + H_2O(g)$	0.10	790.8535	790.8535	6.37×10^{-12}	agree
R9	$Mg(OH)_2 + CO_2 \rightarrow MgCO_3 + H_2O(g)$	0.15	849.5709	849.5709	2.17×10^{-11}	agree
R9	$Mg(OH)_2 + CO_2 \rightarrow MgCO_3 + H_2O(g)$	1.00	1315.5620	1315.5620	2.02×10^{-11}	agree
R9	$Mg(OH)_2 + CO_2 \rightarrow MgCO_3 + H_2O(g)$	20.00	–	–	–	no root in 300-2000 K (both methods)
R10	$MgSiO_3 + CO_2 \rightarrow MgCO_3 + SiO_2$	0.10	432.5140	432.5140	1.44×10^{-10}	agree
R10	$MgSiO_3 + CO_2 \rightarrow MgCO_3 + SiO_2$	0.15	440.2635	440.2635	1.42×10^{-11}	agree
R10	$MgSiO_3 + CO_2 \rightarrow MgCO_3 + SiO_2$	1.00	480.6317	480.6317	6.80×10^{-11}	agree
R10	$MgSiO_3 + CO_2 \rightarrow MgCO_3 + SiO_2$	20.00	562.5846	562.5846	2.36×10^{-11}	agree
R11	$\frac{1}{3}Mg_3Si_2O_5(OH)_4 + CO_2 \rightarrow MgCO_3 + \frac{2}{3}SiO_2 + \frac{2}{3}H_2O(g)$	0.10	376.7372	376.7372	5.75×10^{-10}	agree
R11	$\frac{1}{3}Mg_3Si_2O_5(OH)_4 + CO_2 \rightarrow MgCO_3 + \frac{2}{3}SiO_2 + \frac{2}{3}H_2O(g)$	0.15	391.7046	391.7046	1.71×10^{-11}	agree
R11	$\frac{1}{3}Mg_3Si_2O_5(OH)_4 + CO_2 \rightarrow MgCO_3 + \frac{2}{3}SiO_2 + \frac{2}{3}H_2O(g)$	1.00	481.0166	481.0166	4.70×10^{-11}	agree
R11	$\frac{1}{3}Mg_3Si_2O_5(OH)_4 + CO_2 \rightarrow MgCO_3 + \frac{2}{3}SiO_2 + \frac{2}{3}H_2O(g)$	20.00	753.8233	753.8233	1.73×10^{-9}	agree
R12	$\frac{1}{2}Ca_2SiO_4 + CO_2 \rightarrow CaCO_3 + \frac{1}{2}SiO_2$	0.10	632.8229	632.8229	9.32×10^{-12}	agree
R12	$\frac{1}{2}Ca_2SiO_4 + CO_2 \rightarrow CaCO_3 + \frac{1}{2}SiO_2$	0.15	645.1328	645.1328	3.18×10^{-12}	agree
R12	$\frac{1}{2}Ca_2SiO_4 + CO_2 \rightarrow CaCO_3 + \frac{1}{2}SiO_2$	1.00	710.0484	710.0484	6.93×10^{-12}	agree
R12	$\frac{1}{2}Ca_2SiO_4 + CO_2 \rightarrow CaCO_3 + \frac{1}{2}SiO_2$	20.00	846.2058	846.2058	3.41×10^{-13}	agree
R13	$\frac{1}{2}CaMgSi_2O_6 + CO_2 \rightarrow \frac{1}{2}CaCO_3 + \frac{1}{2}MgCO_3 + SiO_2$	0.10	411.1320	411.1320	1.60×10^{-10}	agree
R13	$\frac{1}{2}CaMgSi_2O_6 + CO_2 \rightarrow \frac{1}{2}CaCO_3 + \frac{1}{2}MgCO_3 + SiO_2$	0.15	418.8687	418.8687	1.75×10^{-9}	agree
R13	$\frac{1}{2}CaMgSi_2O_6 + CO_2 \rightarrow \frac{1}{2}CaCO_3 + \frac{1}{2}MgCO_3 + SiO_2$	1.00	459.4270	459.4270	2.12×10^{-11}	agree
R13	$\frac{1}{2}CaMgSi_2O_6 + CO_2 \rightarrow \frac{1}{2}CaCO_3 + \frac{1}{2}MgCO_3 + SiO_2$	20.00	543.1442	543.1442	2.96×10^{-12}	agree
R14	$CaAl_2Si_2O_8 + CO_2 \rightarrow CaCO_3 + Al_2SiO_5 + SiO_2$	0.10	388.0936	388.0936	1.28×10^{-10}	agree
R14	$CaAl_2Si_2O_8 + CO_2 \rightarrow CaCO_3 + Al_2SiO_5 + SiO_2$	0.15	394.2821	394.2821	5.51×10^{-12}	agree
R14	$CaAl_2Si_2O_8 + CO_2 \rightarrow CaCO_3 + Al_2SiO_5 + SiO_2$	1.00	426.1475	426.1475	2.83×10^{-10}	agree

continued on the next page

Rxn	Reaction	P_{CO_2} / bar	T_{turn} / K extent	T_{turn} / K element pot.	$\lvert\Delta T\rvert$ / K	Status
R14	$CaAl_2Si_2O_8 + CO_2 \rightarrow CaCO_3 + Al_2SiO_5 + SiO_2$	20.00	488.9624	488.9624	1.65×10^{-11}	agree
R15	$CaCO_3 + MgO + CO_2 \rightarrow CaMg(CO_3)_2$	0.10	625.3969	625.3969	8.87×10^{-12}	agree
R15	$CaCO_3 + MgO + CO_2 \rightarrow CaMg(CO_3)_2$	0.15	636.5176	636.5176	1.48×10^{-12}	agree
R15	$CaCO_3 + MgO + CO_2 \rightarrow CaMg(CO_3)_2$	1.00	694.5072	694.5072	5.46×10^{-12}	agree
R15	$CaCO_3 + MgO + CO_2 \rightarrow CaMg(CO_3)_2$	20.00	812.6555	812.6555	3.07×10^{-12}	agree
R17	$Li_2SiO_3 + CO_2 \rightarrow Li_2CO_3 + SiO_2$	0.10	470.6787	470.6787	1.15×10^{-9}	agree
R17	$Li_2SiO_3 + CO_2 \rightarrow Li_2CO_3 + SiO_2$	0.15	479.7566	479.7566	9.83×10^{-11}	agree
R17	$Li_2SiO_3 + CO_2 \rightarrow Li_2CO_3 + SiO_2$	1.00	527.6124	527.6124	6.37×10^{-11}	agree
R17	$Li_2SiO_3 + CO_2 \rightarrow Li_2CO_3 + SiO_2$	20.00	628.6039	628.6039	2.41×10^{-11}	agree
R18	$\frac{1}{3}Mg_3Si_4O_{10}(OH)_2 + CO_2 \rightarrow MgCO_3 + \frac{4}{3}SiO_2 + \frac{1}{3}H_2O(g)$	0.10	339.6668	339.6668	1.87×10^{-10}	agree
R18	$\frac{1}{3}Mg_3Si_4O_{10}(OH)_2 + CO_2 \rightarrow MgCO_3 + \frac{4}{3}SiO_2 + \frac{1}{3}H_2O(g)$	0.15	348.2726	348.2726	9.09×10^{-13}	agree
R18	$\frac{1}{3}Mg_3Si_4O_{10}(OH)_2 + CO_2 \rightarrow MgCO_3 + \frac{4}{3}SiO_2 + \frac{1}{3}H_2O(g)$	1.00	395.1147	395.1147	7.05×10^{-12}	agree
R18	$\frac{1}{3}Mg_3Si_4O_{10}(OH)_2 + CO_2 \rightarrow MgCO_3 + \frac{4}{3}SiO_2 + \frac{1}{3}H_2O(g)$	20.00	502.1710	502.1710	2.13×10^{-9}	agree
R19	$\frac{1}{3}Ca_3Al_2Si_3O_{12} + CO_2 \rightarrow CaCO_3 + \frac{1}{3}Al_2SiO_5 + \frac{2}{3}SiO_2$	0.10	416.6964	416.6964	1.19×10^{-9}	agree
R19	$\frac{1}{3}Ca_3Al_2Si_3O_{12} + CO_2 \rightarrow CaCO_3 + \frac{1}{3}Al_2SiO_5 + \frac{2}{3}SiO_2$	0.15	425.1544	425.1544	2.96×10^{-10}	agree
R19	$\frac{1}{3}Ca_3Al_2Si_3O_{12} + CO_2 \rightarrow CaCO_3 + \frac{1}{3}Al_2SiO_5 + \frac{2}{3}SiO_2$	1.00	469.9841	469.9841	1.04×10^{-9}	agree
R19	$\frac{1}{3}Ca_3Al_2Si_3O_{12} + CO_2 \rightarrow CaCO_3 + \frac{1}{3}Al_2SiO_5 + \frac{2}{3}SiO_2$	20.00	565.2773	565.2773	8.15×10^{-11}	agree
R20	$\frac{1}{3}Mg_3Al_2Si_3O_{12} + CO_2 \rightarrow MgCO_3 + \frac{1}{3}Al_2SiO_5 + \frac{2}{3}SiO_2$	0.10	472.0258	472.0258	1.25×10^{-9}	agree
R20	$\frac{1}{3}Mg_3Al_2Si_3O_{12} + CO_2 \rightarrow MgCO_3 + \frac{1}{3}Al_2SiO_5 + \frac{2}{3}SiO_2$	0.15	480.1544	480.1544	7.49×10^{-11}	agree
R20	$\frac{1}{3}Mg_3Al_2Si_3O_{12} + CO_2 \rightarrow MgCO_3 + \frac{1}{3}Al_2SiO_5 + \frac{2}{3}SiO_2$	1.00	522.3566	522.3566	6.39×10^{-10}	agree
R20	$\frac{1}{3}Mg_3Al_2Si_3O_{12} + CO_2 \rightarrow MgCO_3 + \frac{1}{3}Al_2SiO_5 + \frac{2}{3}SiO_2$	20.00	607.3494	607.3494	1.59×10^{-12}	agree
R21	$\frac{1}{3}Ca_2MgSi_2O_7 + CO_2 \rightarrow \frac{2}{3}CaCO_3 + \frac{1}{3}MgCO_3 + \frac{2}{3}SiO_2$	0.10	522.3971	522.3971	6.59×10^{-10}	agree

continued on the next page

Rxn	Reaction	P_{CO_2} / bar	T_{turn} / K extent	T_{turn} / K element pot.	$\lvert\Delta T\rvert$ / K	Status
R21	$\frac{1}{3}Ca_2MgSi_2O_7 + CO_2 \rightarrow \frac{2}{3}CaCO_3 + \frac{1}{3}MgCO_3 + \frac{2}{3}SiO_2$	0.15	531.9187	531.9187	2.52×10^{-11}	agree
R21	$\frac{1}{3}Ca_2MgSi_2O_7 + CO_2 \rightarrow \frac{2}{3}CaCO_3 + \frac{1}{3}MgCO_3 + \frac{2}{3}SiO_2$	1.00	581.7572	581.7572	1.66×10^{-11}	agree
R21	$\frac{1}{3}Ca_2MgSi_2O_7 + CO_2 \rightarrow \frac{2}{3}CaCO_3 + \frac{1}{3}MgCO_3 + \frac{2}{3}SiO_2$	20.00	684.3399	684.3399	1.02×10^{-11}	agree
R22	$\frac{1}{4}Ca_3Mg(SiO_4)_2 + CO_2 \rightarrow \frac{3}{4}CaCO_3 + \frac{1}{4}MgCO_3 + \frac{1}{2}SiO_2$	0.10	586.6825	586.6825	1.41×10^{-11}	agree
R22	$\frac{1}{4}Ca_3Mg(SiO_4)_2 + CO_2 \rightarrow \frac{3}{4}CaCO_3 + \frac{1}{4}MgCO_3 + \frac{1}{2}SiO_2$	0.15	597.6310	597.6310	4.32×10^{-12}	agree
R22	$\frac{1}{4}Ca_3Mg(SiO_4)_2 + CO_2 \rightarrow \frac{3}{4}CaCO_3 + \frac{1}{4}MgCO_3 + \frac{1}{2}SiO_2$	1.00	655.0962	655.0962	7.62×10^{-12}	agree
R22	$\frac{1}{4}Ca_3Mg(SiO_4)_2 + CO_2 \rightarrow \frac{3}{4}CaCO_3 + \frac{1}{4}MgCO_3 + \frac{1}{2}SiO_2$	20.00	774.2267	774.2267	4.77×10^{-12}	agree
R23	$\frac{1}{3}Ca_3Si_2O_7 + CO_2 \rightarrow CaCO_3 + \frac{2}{3}SiO_2$	0.10	571.6753	571.6753	3.01×10^{-10}	agree
R23	$\frac{1}{3}Ca_3Si_2O_7 + CO_2 \rightarrow CaCO_3 + \frac{2}{3}SiO_2$	0.15	582.7795	582.7795	1.60×10^{-11}	agree
R23	$\frac{1}{3}Ca_3Si_2O_7 + CO_2 \rightarrow CaCO_3 + \frac{2}{3}SiO_2$	1.00	641.3561	641.3561	5.80×10^{-12}	agree
R23	$\frac{1}{3}Ca_3Si_2O_7 + CO_2 \rightarrow CaCO_3 + \frac{2}{3}SiO_2$	20.00	764.3941	764.3941	1.13×10^{-11}	agree
R24	$CaAl_2Si_2O_8 + CO_2 + 2H_2O(g) \rightarrow CaCO_3 + Al_2Si_2O_5(OH)_4$	0.10	415.4393	415.4393	8.23×10^{-10}	agree
R24	$CaAl_2Si_2O_8 + CO_2 + 2H_2O(g) \rightarrow CaCO_3 + Al_2Si_2O_5(OH)_4$	0.15	418.2008	418.2008	1.63×10^{-9}	agree
R24	$CaAl_2Si_2O_8 + CO_2 + 2H_2O(g) \rightarrow CaCO_3 + Al_2Si_2O_5(OH)_4$	1.00	431.6409	431.6409	1.78×10^{-10}	agree
R24	$CaAl_2Si_2O_8 + CO_2 + 2H_2O(g) \rightarrow CaCO_3 + Al_2Si_2O_5(OH)_4$	20.00	454.7836	454.7836	7.67×10^{-12}	agree

Table S4.3 Equilibrium extent from the two methods, summarized per reaction over the state grid. "Interior" counts states with a genuine interior minimum; the remaining sampled states have a minimum at one of the physical bounds. A boundary minimum does not by itself imply that $G(\xi)$ is linear, because Class II profiles and inert-containing Class III profiles may be curved while minimized at a bound. The two methods agree on the selected bound in every such state.

Rxn	states	interior	max $\lvert\Delta\xi_{eq}\rvert$	max atom residual / mol	max $\lvert\Delta G\rvert$ / kJ
R1	16	3	1.97×10^{-7}	4.44×10^{-16}	1.09×10^{-6}
R2	16	7	4.12×10^{-8}	4.44×10^{-16}	2.48×10^{-6}
R3	16	14	3.46×10^{-8}	8.88×10^{-16}	1.49×10^{-10}
R4	16	1	2.51×10^{-8}	8.88×10^{-16}	1.19×10^{-6}
R5	16	0	1.01×10^{-8}	4.44×10^{-16}	1.38×10^{-6}
R6	16	4	2.51×10^{-8}	8.88×10^{-16}	1.54×10^{-6}
R7	16	6	5.86×10^{-8}	8.88×10^{-16}	2.02×10^{-6}
R8	16	6	2.51×10^{-8}	2.22×10^{-16}	1.88×10^{-6}
R9	16	16	9.54×10^{-8}	8.88×10^{-16}	6.82×10^{-13}
R10	16	0	1.01×10^{-8}	0	1.41×10^{-6}
R11	16	16	4.44×10^{-7}	1.78×10^{-15}	6.37×10^{-12}
R12	16	3	6.43×10^{-8}	1.11×10^{-16}	9.70×10^{-7}
R13	16	0	1.01×10^{-8}	8.88×10^{-16}	1.36×10^{-6}
R14	16	0	1.01×10^{-8}	1.78×10^{-15}	1.68×10^{-6}
R15	16	3	2.83×10^{-8}	8.88×10^{-16}	1.08×10^{-6}
R17	16	1	4.86×10^{-8}	8.88×10^{-16}	1.00×10^{-6}
R18	16	2	1.01×10^{-8}	8.88×10^{-16}	3.66×10^{-7}
R19	16	0	1.01×10^{-8}	1.11×10^{-16}	1.24×10^{-6}
R20	16	1	1.38×10^{-7}	2.22×10^{-16}	1.39×10^{-6}
R21	16	1	3.23×10^{-8}	8.88×10^{-16}	1.22×10^{-6}
R22	16	3	2.02×10^{-7}	4.44×10^{-16}	1.09×10^{-6}
R23	16	1	2.51×10^{-8}	8.88×10^{-16}	1.06×10^{-6}
R24	16	0	1.01×10^{-8}	2.22×10^{-16}	4.39×10^{-6}

S5. Supporting figures

Figures S1–S5 show the adopted-input Gibbs profiles, conversion curves, reaction properties and conversion maps. Source and fugacity sensitivity figures are presented in Section S7.

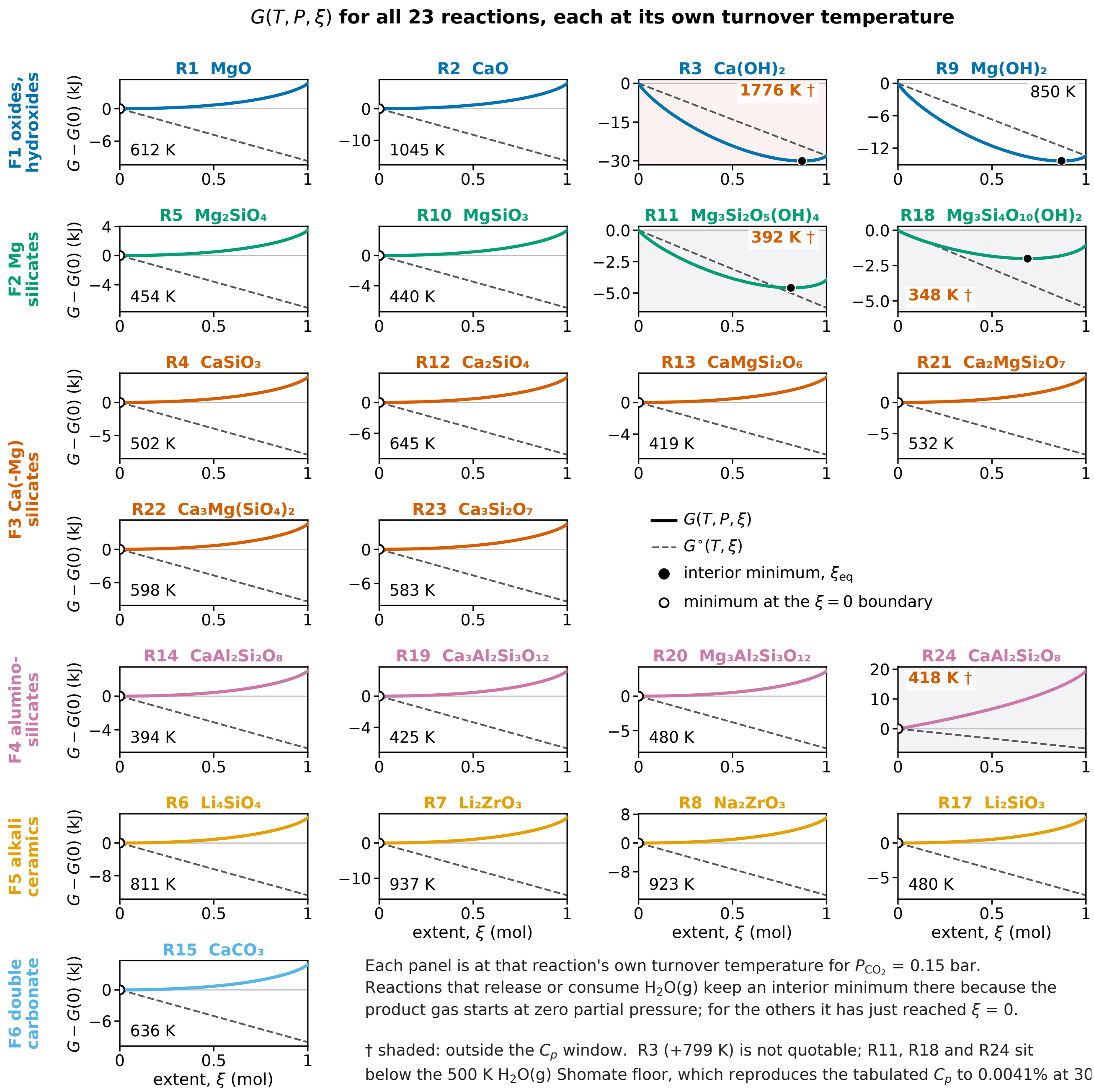

Fig. S1 Total Gibbs energy against extent of reaction for all 23 reactions, grouped by family, each panel evaluated at that reaction's own turnover temperature at $P_{CO_2} = 0.15$ bar under the five gas conditions. The Class I construction illustrated in the article generalizes to other reactions: a dry undiluted charge has a linear profile, with an endpoint minimum except at the degenerate transition boundary. Dilution introduces gas-mixing curvature and can produce an interior minimum. Class II reactions can have the same curvature without added inert because water is produced. A curved profile can still minimize at a feasible endpoint. The underlying equilibrium states and turnover temperatures are given in Tables S2.2 and S2.4.

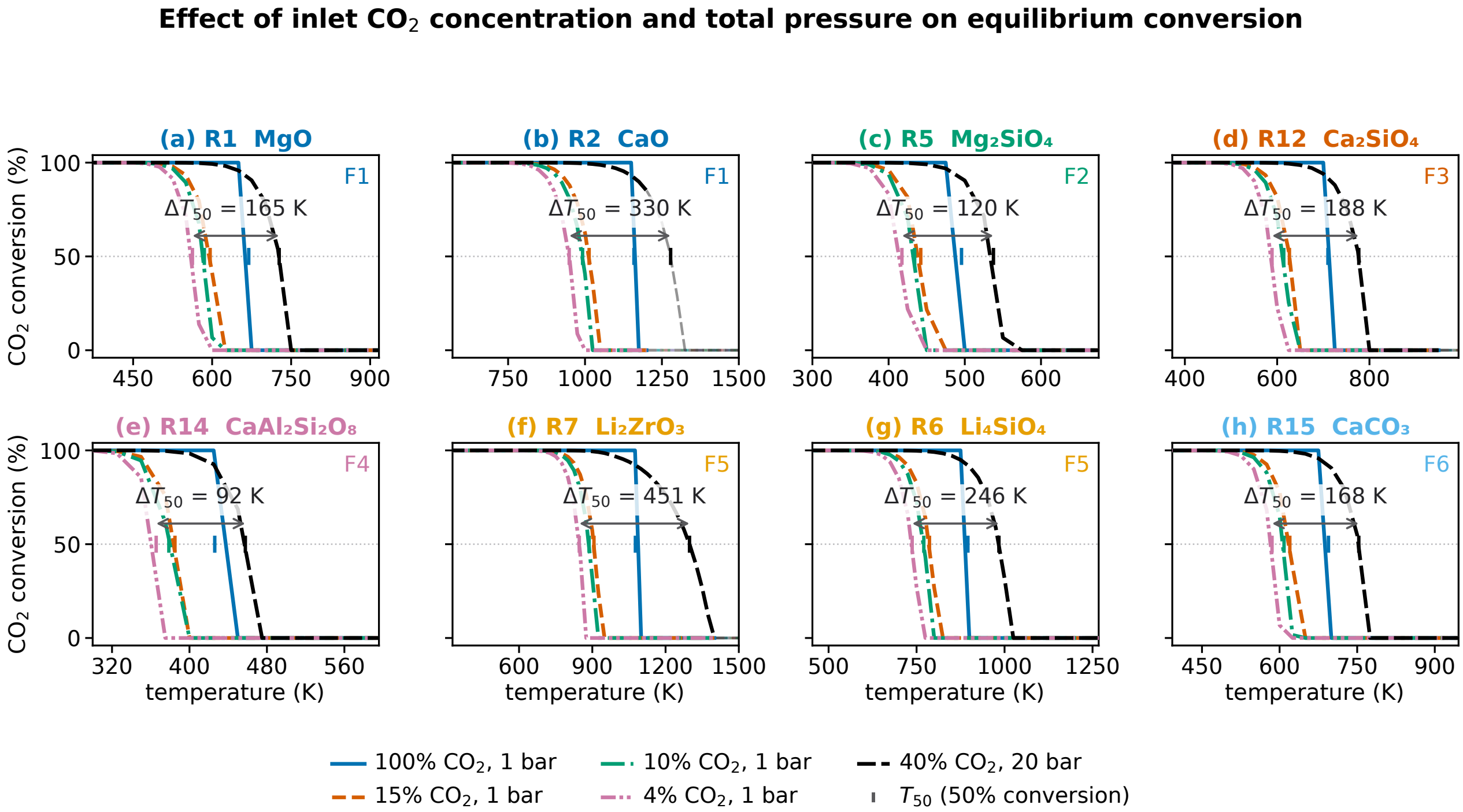

Fig. S2 Effect of inlet CO_2 concentration and total pressure on equilibrium conversion, for eight representative reactions covering all six families, under the five gas conditions. Markers give the temperature at which conversion passes 50%, and the bar beneath each panel gives the span of that temperature across the conditions. All eight reactions shown are Class I, so the undiluted condition is a pure CO_2 gas for each of them. Over the 18 Class I reactions, all of which respond, the shift between pure CO_2 and 4% CO_2 has a full-set diagnostic median of 100.5 K, a minimum of 60.5 K and a maximum of 229.8 K for Li_2ZrO_3. The Class II reactions respond less, by a median of 58.9 K over the three for which the comparison is defined, and brucite does not respond at all: it releases one mole of H_2O(g) per mole of CO_2 consumed, so $\Delta\nu_{gas} = 0$ and its ideal-gas equilibrium is independent of inert loading and total pressure when the initial reactive charge is held fixed. The 229.8 K maximum is conditional on the adopted sources and phase treatment (Section S7.3). The underlying values are given in Table S2.2.

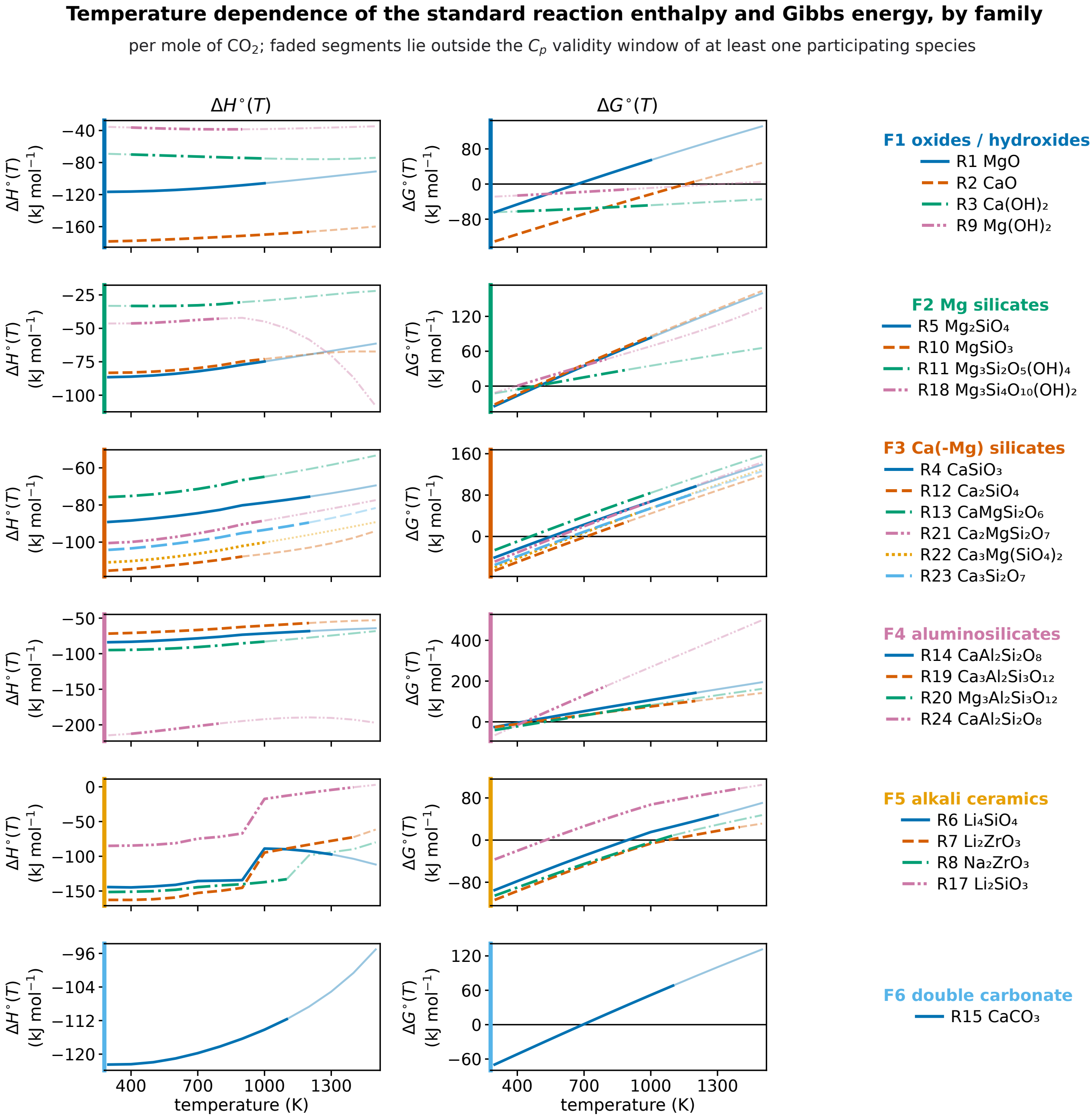

Fig. S3 Standard reaction enthalpy and standard reaction Gibbs energy against temperature from 298 to 1500 K, by family, per mole of CO_2 consumed. Within their respective heat-capacity validity windows, all 23 reactions remain exothermic; plotted extensions outside those windows are shown for context and are not treated as results. Within the valid ranges, ΔG° rises with temperature at a slope set by $|\Delta S^\circ|$. The two hydroxide reactions that release one mole of H_2O(g) per mole of CO_2 stand apart: their small $|\Delta S^\circ|$ gives an almost flat $\Delta G^\circ(T)$, which makes a temperature inferred from a zero crossing especially sensitive to thermochemical data and water convention. Standard reaction properties alone do not specify conversion in a finite gas charge. The underlying values are given in Table S2.2.

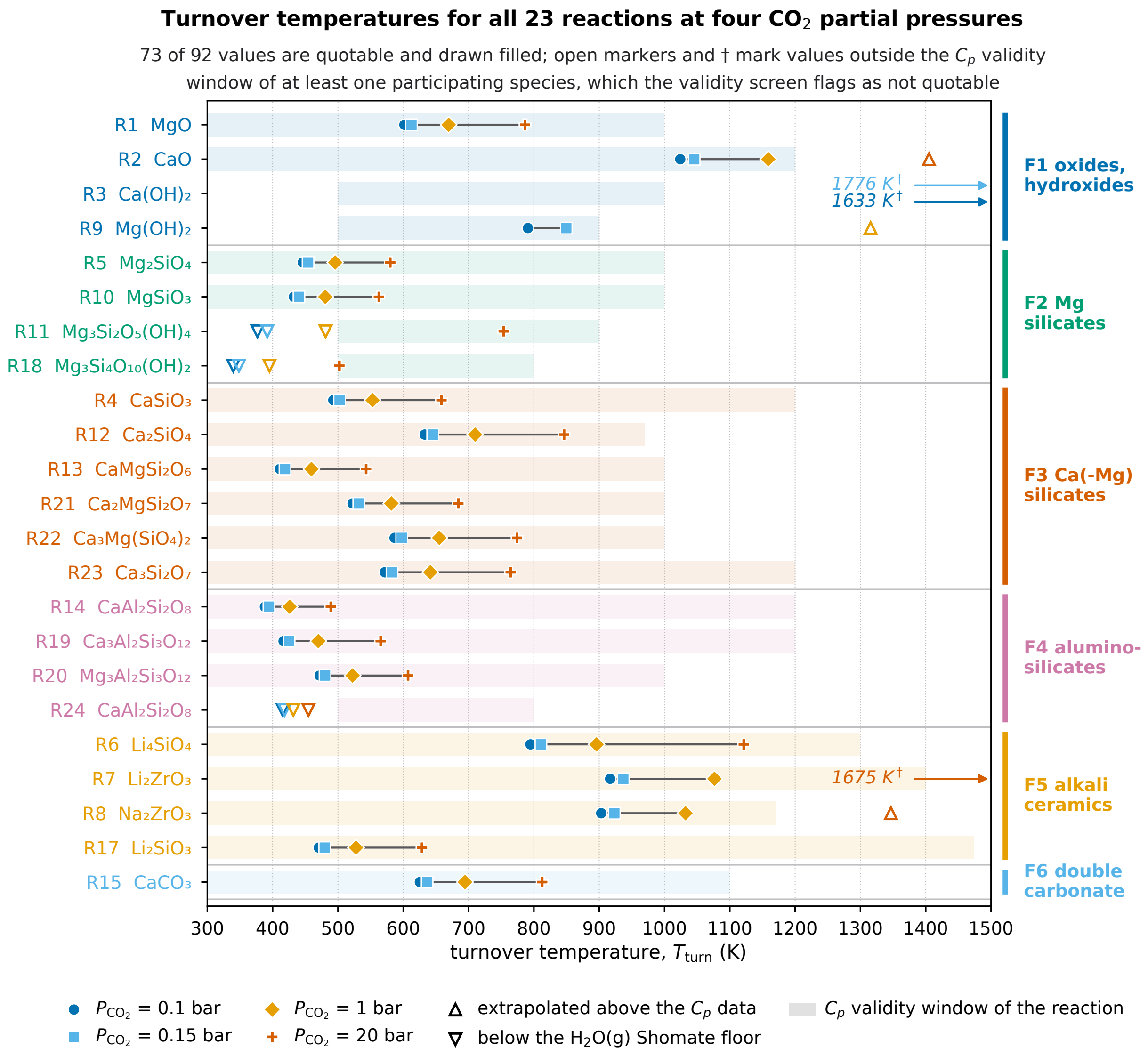

Fig. S4 Turnover temperatures for all 23 reactions at four CO_2 partial pressures, drawn against the temperature interval in which every participating species has heat-capacity data, shown as the shaded band behind each row. This is the graphical form of Table S2.4. The article separates Class I turnover values (Table 1) from the water conventions (Table 2). A marker outside its band is a conditional extrapolation; sixteen of the 92 values are in that position, and the two portlandite values extend furthest. The rows for R3, R9, R11, R18 and R24 are Class II or Class III: $H_2O(g)$ enters their reaction quotient, so their turnover temperature is not a function of P_{CO_2} alone, and the markers drawn for them are the $p_{H_2O} = 1$ bar values of Tables S2.4 and S2.5.

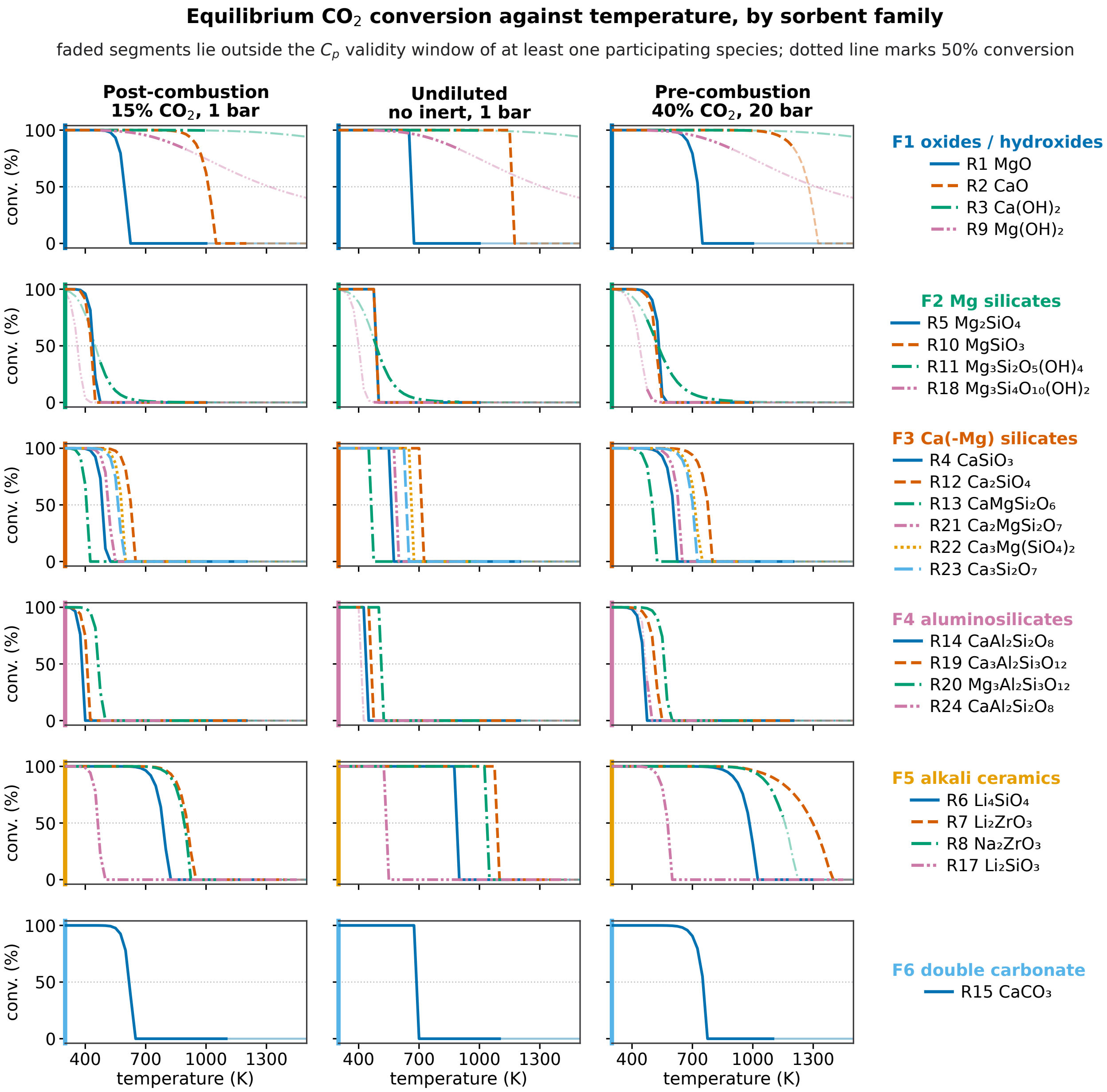

Fig. S5 Equilibrium conversion maps for all 23 reactions, grouped by family. The columns represent nominal 15% CO_2 at 1 bar, undiluted CO_2 at 1 bar, and nominal 40% CO_2 at 20 bar. Temperature extensions outside the common heat-capacity ranges are faded. R24 includes two initial moles of water per mole of CO_2; its actual inlet fractions $(y_{CO_2}, y_{H_2O}, y_{N_2})$ are $(0.1154, 0.2308, 0.6538)$, $(1/3, 2/3, 0)$ and $(0.2222, 0.4444, 0.3333)$, respectively. The dry-reactive-feed labels therefore do not denote its three-component gas fractions. These maps describe each restricted reaction and do not include competing phases or water condensation. R15 denotes carbonation of the $CaCO_3 + MgO$ reactant pair to dolomite; the short plot label identifies its carbonate component.

S6. Candidates outside the admitted data set

The admission rule is that a reaction enters the set only if the standard formation enthalpy, the standard entropy and the heat capacity of every participating species have a citable primary or critically assessed source, with the lineage recorded in Table S1. Three anchors fail even that test and are carried with an explicit label; they are named in Section S1 and in Table S1. Nothing in the database is estimated by Neumann–Kopp oxide additivity or interpolated between compounds. The set that results is 23 reactions across six families. What follows records the candidates that the rule excluded, and why.

S6.1 Rejected for a missing primary datum

$Na_2CO_3 + CO_2 + H_2O(g) \longrightarrow 2NaHCO_3$, the dry carbonate-looping route (register identifier R16).

No temperature-dependent heat-capacity data set for $NaHCO_3$ meeting the study's traceability and validity criteria was established from the sources examined. Robie and Hemingway give $\Delta_f H^\circ(298) = -949.0 \pm 0.2$ kJ mol^{-1} and $S^\circ(298) = 102.1 \pm 1.7$ J mol^{-1} K^{-1} for nahcolite, but no temperature-dependent coefficients are supplied in the corresponding tables. This exclusion concerns the traceable inputs available to this study, not the existence of heat-capacity information elsewhere. A traceable curve and temperature interval would permit this one missing species to be assessed.

Its 298 K standard properties, formed from the cited anchors, are $\Delta H^\circ(298) = -131.95$ and $\Delta G^\circ(298) = -32.55$ kJ per mole CO_2, against -135.34 and -34.13 from the assessed-database column of the first-principles screening study and -153.82 and -58.74 from its density functional theory column. Relative to the assessed-database column, these values differ by 3.39 kJ mol^{-1} in ΔH° and 1.58 kJ mol^{-1} in ΔG°; relative to the first-principles column, the corresponding differences are 21.87 and 26.19 kJ mol^{-1}.

Li_8SiO_6, a lithium-rich ceramic candidate. Missing data: $\Delta_f H^\circ(298)$, $S^\circ(298)$ and $C_p(T)$, all three. The compound has a real experimental CO_2 chemisorption literature, but those papers report uptake kinetics and capacity, not formation thermochemistry. No calorimetric formation enthalpy, no third-law entropy and no measured heat capacity was located. Estimating the phase by oxide additivity is precisely the practice the admission rule excludes, and was not done.

S6.2 Deferred as outside the reaction scope

Robie and Hemingway provide high-temperature tables for hatrurite Ca_3SiO_5, gehlenite $Ca_2Al_2SiO_7$, monticellite $CaMgSiO_4$, calcio-olivine γ-Ca_2SiO_4, spurrite, tilleyite and chrysotile $Mg_3Si_2O_5(OH)_4$. Chrysotile is included: its fitted heat capacity reproduces the tabulated values (p. 394) to 0.111%. At 298.15 K, the fit gives 273.75 J mol^{-1} K^{-1} against 274.05 in the source table. The oxide-additivity estimate of 281.0 is a contextual comparison and is not used as an input.

The other six phases lie outside the selected reaction set. None of them appears in any of the 23 reactions of this study. Hatrurite, gehlenite, monticellite, spurrite and tilleyite are additional clinker and skarn phases that would extend the scope rather than complete it. Calcio-olivine is the low-temperature Ca_2SiO_4 polymorph, whose admission would require the β to γ relation to be modeled rather than a single polymorph chosen (Section 5.1 of the article). Their exclusion is a scope decision, not a data-availability finding. Hatrurite is otherwise ready: its heat capacity recomputed at 298.15 K from the printed equation, 171.6 J mol^{-1} K^{-1}, matches the accepted value.

S6.3 Alternatives located, evaluated and not adopted

The alternatives fall into two groups. Three concern the magnesite anchor: -1111.68 kJ mol^{-1} from the reversed phase equilibrium of Koziol and Newton, -1113.30 from Robie and Hemingway and -1114.51 from Chernosky and Berman. The two more negative of them are worth $+2.5$ to $+9.5$ K and $+4.3$ to $+16.7$ K respectively on the

turnover temperatures of the seven Class I magnesite-forming reactions at 1 bar. Two other magnesite formers lie outside those ranges, serpentine R11 at +23.3 and +40.7 K and talc R18 at +13.7 and +24.1 K, as does brucite R9, whose small reaction enthalpy makes it the most sensitive row in the set.

The remaining alternatives concern other species or phases. They are the older NBS anchor −1095.80 for magnesite, the 1967 NIST-JANAF enstatite entry, and andalusite in place of kyanite as the Al_2SiO_5 polymorph, the last worth at most 1.3 K on any imposed-pressure Class I turnover, so that the choice does not matter numerically for the turnover temperatures. On the closed-charge quantities of the same scenario it is larger, 3.5 K on the R14 4% T_{50} and 2.2 K on the R14 dilution shift. The fourth is Kleykamp's Li_2SiO_3 heat capacity in place of the JANAF assessment. That is a 5.2% source disagreement worth at most 2.04 K on any turnover or target temperature in this study, and it affects R6 and R17. The seven compilation-disagreement anchors introduced by the source-attribution audit of Section S1 and the three reaction-level alternatives for R6, R7 and R17 are listed with their effects in Section S7.3; with the andalusite case that makes 23 scenarios in all.

With all phase transitions retained, the alternative Li_2SiO_3 heat capacity changes the Li_4SiO_4 turnover by −1.9 K at 0.1 bar and −1.4 K at 20 bar. Its largest effect in the target grid is −2.04 K for R6 at 1 bar. This isolates heat-capacity sensitivity from the separate solid-carbonate diagnostic of Section S7.3.

S6.4 Out of scope by design

Four classes of candidate are out of scope by design, and are named so that no reader assumes they were overlooked. Aqueous and brine carbonation routes of every mineral in this set are excluded, the work being strictly solid–gas. Hydrated magnesium carbonates (nesquehonite, hydromagnesite, artinite) are excluded because their phase and water-activity relations require an expanded species set. Solid solutions of any kind are excluded, every phase here being treated as a pure stoichiometric condensed phase. Iron-bearing phases are excluded because they would require an oxygen-fugacity variable the formalism does not carry, although complete data for them do exist.

S7. Target conversion and conditional sensitivity analyses

S7.1 Closed-charge target temperatures

All targets use one initial mole of CO_2, the stoichiometric condensed reactant charge and a constant total pressure. For R3, R9, R11 and R18 the initial water charge is zero unless specified. R24 receives two initial moles of water per mole of CO_2 in the target grid. Consequently, its nominal dry-basis CO_2 fraction does not equal its actual inlet fraction. The CSV reports both definitions explicitly. For a target conversion c, the equilibrium condition is

$$F(T;c) = \Delta_r G^\circ(T) + RT \sum_{i\in\text{reactive gas}} \nu_i \ln\left[\frac{n_{i,0}+\nu_i c}{\sum_{j\in\text{gas}}(n_{j,0}+\nu_j c)}\frac{P}{P^\circ}\right] = 0, \tag{2}$$

where $P^\circ = 1$ bar and every consumed gas must remain positive at c. Targets are fractions of the initial CO_2, not fractions of the water-limited maximum. Both T_{50} and T_{90} are reported. In a dry undiluted Class I charge, or the undiluted stoichiometric R24 charge, the quotient is extent-independent; the reported root denotes the transition boundary and does not select a unique conversion.

The complete target grid contains 460 rows: 23 reactions, five nominal CO_2 fractions (0.04, 0.10, 0.15, 0.40 and 1.00), two total pressures (1 and 20 bar), and two target conversions. Table S7.1 gives the 15%, 1 bar selection. Superscripts L and H indicate roots below or above the common heat-capacity interval; unmarked temperatures lie within it. NR means no crossing was located in 300–2000 K. These labels do not certify global phase stability or the absence of condensation. The full grid and its status fields are in `Table_S7_1_target_conversion_grid.csv`.

Table S7.1 Closed-charge target temperatures at nominal 15% CO_2 and 1 bar. R24 has actual inlet fractions $(0.1154, 0.2308, 0.6538)$ for CO_2, H_2O and N_2.

Reaction	Sorbent	T_{50} / K	T_{90} / K
R1	MgO	596.2	558.9
R2	CaO	1013.4	941.6
R3	$Ca(OH)_2$	NR	1667.2^{H}
R4	$CaSiO_3$	488.1	455.6
R5	Mg_2SiO_4	442.2	414.8
R6	Li_4SiO_4	786.7	732.1
R7	Li_2ZrO_3	906.8	840.2
R8	Na_2ZrO_3	893.1	826.0
R9	$Mg(OH)_2$	1315.6^{H}	805.3
R10	$MgSiO_3$	428.6	402.0
R11	$Mg_3Si_2O_5(OH)_4$	449.5^{L}	367.2^{L}
R12	Ca_2SiO_4	626.6	584.8
R13	$CaMgSi_2O_6$	407.2	380.8
R14	$CaAl_2Si_2O_8$	385.0	363.5
R15	$CaCO_3 + MgO$	619.8	581.6
R17	Li_2SiO_3	466.1	435.2
R18	$Mg_3Si_4O_{10}(OH)_2$	361.2^{L}	323.9^{L}
R19	$Ca_3Al_2Si_3O_{12}$	412.5	383.8
R20	$Mg_3Al_2Si_3O_{12}$	467.9	439.9
R21	$Ca_2MgSi_2O_7$	517.6	485.0
R22	$Ca_3Mg(SiO_4)_2$	581.2	543.8
R23	$Ca_3Si_2O_7$	566.1	528.3
R24	$CaAl_2Si_2O_8$	388.3^{L}	364.7^{L}

S7.2 Matched inlet humidity

To distinguish gas stoichiometry from feed composition, the five water-producing or water-consuming reactions are compared at identical actual inlet fractions: $y_{CO_2,0} = 0.15$, $y_{H_2O,0} = 0$, 0.05, 0.10, 0.30 or 0.60, and $y_{N_2,0} = 0.85 - y_{H_2O,0}$, at 1 bar. Initial molar charges are $n_{CO_2,0} = 1$, $n_{H_2O,0} = y_{H_2O,0}/0.15$ and $n_{N_2,0} = y_{N_2,0}/0.15$. For R24, $c_{max} = \min[1, y_{H_2O,0}/(2y_{CO_2,0})]$; a target at or beyond this limit is labeled FL. Thus, an initially dry R24 feed cannot carbonate, while its baseline stoichiometric-water charge can.

Water production suppresses the forward reaction through its product activity; added water has the opposite stoichiometric role in R24. At a specified temperature, R3 and R9 can also be described by

$$\left(\frac{p_{H_2O}}{p_{CO_2}}\right)_{eq} = \exp\left[-\frac{\Delta_r G^\circ}{RT}\right]. \tag{3}$$

This ratio is independent of ideal-gas total pressure, but the conversion required to attain it depends on the initial humidity. At 500, 700 and 900 K, R9 gives ratios 2.9×10^2, 21 and 4.9; R3 gives 2.0×10^6, 1.5×10^4 and 9.0×10^2. The ratio depends exponentially on $\Delta_r G^\circ$, so two significant figures are quoted. All six ratios lie within the respective heat-capacity windows. Full-precision ratios accompany the 50 matched-humidity target rows in the supplementary CSV files. Low-temperature hydrous roots are retained with L flags; the restricted ideal-gas calculation does not permit condensed water.

Table S7.2 Matched actual inlet humidity at $y_{CO_2,0} = 0.15$ and $P = 1$ bar. L/H: heat-capacity extrapolation; NR: no root in 300–2000 K; FL: target at or beyond the available reactive-gas charge.

Reaction	$y_{H_2O,0}$	c_{max}	T_{50} / K	T_{90} / K
R3	0.00	1.000	NR	1667.2^{H}
R3	0.05	1.000	NR	1571.1^{H}
R3	0.10	1.000	NR	1506.9^{H}
R3	0.30	1.000	1906.6^{H}	1366.1^{H}
R3	0.60	1.000	1667.2^{H}	1266.4^{H}
R9	0.00	1.000	1315.6^{H}	805.3
R9	0.05	1.000	1142.5^{H}	763.5
R9	0.10	1.000	1053.2^{H}	734.6
R9	0.30	1.000	896.9	669.0
R9	0.60	1.000	805.3	621.5
R11	0.00	1.000	449.5^{L}	367.2^{L}
R11	0.05	1.000	427.3^{L}	357.5^{L}
R11	0.10	1.000	415.3^{L}	351.1^{L}
R11	0.30	1.000	392.3^{L}	336.9^{L}
R11	0.60	1.000	377.0^{L}	326.4^{L}
R18	0.00	1.000	361.2^{L}	323.9^{L}
R18	0.05	1.000	352.8^{L}	319.2^{L}
R18	0.10	1.000	349.0^{L}	316.7^{L}
R18	0.30	1.000	342.2^{L}	311.6^{L}
R18	0.60	1.000	337.7^{L}	308.0^{L}
R24	0.00	0.000	FL	FL
R24	0.05	0.167	FL	FL
R24	0.10	0.333	FL	FL
R24	0.30	1.000	394.2^{L}	371.1^{L}
R24	0.60	1.000	408.0^{L}	398.9^{L}

S7.3 Paired source and phase sensitivities

Each source variant is applied consistently to every reaction containing the affected species and to both members of the pure/4% feed pair. The dilution shift is $T_{50}(y_{CO_2,0} = 1) - T_{50}(y_{CO_2,0} = 0.04)$ at 1 bar. The two temperatures are not perturbed independently. The 414 paired rows comprise 23 specified scenarios for 18 Class I reactions. Eleven of the 23 were added in this revision. Seven are anchor scenarios in which a species whose two consulted compilations disagree by more than the printed uncertainty takes the alternative value. Those seven are $CaCO_3$ and $CaSiO_3$ from Robie and Hemingway in place of the NBS Tables, Li_2CO_3, Li_2SiO_3, Mg_2SiO_4 and Na_2CO_3 from NIST-JANAF in place of the NBS Tables, and ZrO_2 from NIST-JANAF in place of Robie and Hemingway. Three more are reaction-level anchors taken from published 300 K reaction properties for R6, R7 and R17. The eleventh is the HSC-Chemistry comparison column printed beside the R7 first-principles values. These are deterministic conditional sensitivities, not confidence intervals or a complete uncertainty budget; covariance information for a joint probabilistic treatment was not established.

The selected magnesite enthalpy alternatives are -1111.68, -1113.30 and -1114.51 kJ mol^{-1}. They represent a specified literature-anchor range, not an exhaustive range of all published values. Entropy and heat capacity remain fixed when this anchor alone is varied. A separate stress calculation uses the literal JANAF printed center and uncertainty, -1111.7 ± 8 kJ mol^{-1}, hence endpoints -1119.7 and -1103.7. This older entry was reviewed in 1966; the 1998 compilation date is not its measurement date. The specified literature alternatives produce Class I full-set diagnostic median shifts of 100.5–101.4 K, whereas the JANAF endpoint stress spans 96.5–102.7 K.

The alternative Li_2SiO_3 heat capacity is Kleykamp's measured-enthalpy representation over 298–1400 K,

$$C_p^\circ = 125.860 + 0.03426T - 3614467/T^2 \quad \mathrm{J\,mol^{-1}\,K^{-1}}, \tag{4}$$

with T in kelvin. Its integrated enthalpy and entropy are anchored to the same 298.15 K values as the adopted case, isolating the heat-capacity effect. This alternative is paired with each selected magnesite anchor. A metastable solid-only carbonate case isolates the effect of suppressing melting; it is not an alternative prediction of stable high-temperature phases.

Table S7.3 Paired dilution-shift statistics across the 18 Class I reactions at 1 bar. Medians and maxima refer to the reaction set, not to an individual material. All unspecified thermochemical inputs remain fixed.

Specified scenario	n	Median / K	Maximum / K
Adopted sources and carbonate phases	18	100.5	229.8
$MgCO_3$ anchor -1111.68 kJ mol^{-1} (Koziol and Newton)	18	100.5	229.8
$MgCO_3$ anchor -1113.30 kJ mol^{-1} (Robie and Hemingway)	18	101.1	229.8
$MgCO_3$ anchor -1114.51 kJ mol^{-1} (Chernosky and Berman)	18	101.4	229.8
-1111.68 anchor with the Kleykamp Li_2SiO_3 C_p	18	100.5	229.8
-1113.30 anchor with the Kleykamp Li_2SiO_3 C_p	18	101.1	229.8
-1114.51 anchor with the Kleykamp Li_2SiO_3 C_p	18	101.4	229.8
JANAF enthalpy endpoint stress: -1119.7 kJ mol^{-1}	18	102.7	229.8
JANAF enthalpy endpoint stress: -1103.7 kJ mol^{-1}	18	96.5	229.8
Metastable solid-only carbonate continuation	18	100.5	210.4
R7 reaction anchor, Duan 2011 JRSE Table VI at 300 K	18	100.5	210.4
R7 Duan 2011 anchor with the solid-only continuation	18	100.5	210.4
Al_2SiO_5 polymorph: andalusite in place of kyanite	18	100.5	229.8
$CaCO_3$ anchors: Robie and Hemingway for the NBS Tables	18	100.8	229.8
$CaSiO_3$ anchors: Robie and Hemingway for the NBS Tables	18	100.7	229.8
Li_2CO_3 anchors: NIST-JANAF for the NBS Tables	18	100.5	229.1
Li_2SiO_3 anchors: NIST-JANAF for the NBS Tables	18	100.5	229.8
Mg_2SiO_4 anchors: NIST-JANAF for the NBS Tables	18	100.5	229.8
Na_2CO_3 anchors: NIST-JANAF for the NBS Tables	18	100.5	229.8
ZrO_2 anchors: NIST-JANAF for Robie and Hemingway	18	100.5	210.4
R17 reaction anchor, Duan et al. 2013 PCCP Table 5 at 300 K	18	100.5	229.8
R6 reaction anchor, Duan et al. 2013 PCCP Table 5 at 300 K	18	100.5	229.8

Specified scenario	n	Median / K	Maximum / K
R7 reaction anchor, the HSC-Chemistry column of Duan 2011 note a	18	100.5	229.1

The largest adopted-input shift, 229.8 K for R7 (Li_2ZrO_3), requires its own source test because changing magnesite does not affect that reaction. Duan's Table VI reports the R7 reaction pair at *300 K*: $\Delta_r H^\circ = -158.562$ and $\Delta_r G^\circ = -103.845$ kJ mol^{-1}, implying $\Delta_r S^\circ = -182.39$ J mol^{-1} K^{-1}. Replacing this pair consistently at 300 K while retaining the present heat-capacity integration yields the diagnostic in Table S7.4. The same source prints an HSC-Chemistry comparison pair in its footnote a, -162.69 and -113.18 kJ mol^{-1}, which is carried as a separate scenario and gives 229.1 K. A third alternative replaces the ZrO_2 anchors by the NIST-JANAF pair and gives 210.2 K. None of these is a reproduction of the complete first-principles calculation or a measured alternative formation enthalpy for one species. The specified R7 source/phase cases span 150.9–229.8 K. Under the smaller R7 shifts, CaO becomes the largest-shift reaction at 210.4 K; the range for R7 must therefore not be reported as the range of the maximum across all reactions.

Table S7.4 Source and phase diagnostics at 1 bar for R6, R7 and R17. For each of R7, R6 and R17 the table lists the reference case, every scenario of Table S7.3 that moves that reaction's shift, and its own reaction-level alternative even where that alternative moves the shift by less than 0.1 K. Pure-feed entries denote the phase boundary; dilute entries are closed-charge T_{50}. All displayed roots are within the adopted reaction heat-capacity interval.

Rxn	Specified case	Pure / K	4% / K	Shift / K
R7	Adopted sources and carbonate phases	1076.5	846.7	229.8
R7	Metastable solid-only carbonate continuation	1047.6	846.7	200.9
R7	R7 reaction anchor, Duan 2011 JRSE Table VI at 300 K	900.2	749.3	150.9
R7	Li_2CO_3 anchors: NIST-JANAF for the NBS Tables	1075.6	846.5	229.1
R7	ZrO_2 anchors: NIST-JANAF for Robie and Hemingway	1038.7	828.5	210.2
R7	R7 reaction anchor, the HSC-Chemistry column of Duan 2011 note a	1075.2	846.1	229.1
R6	Adopted sources and carbonate phases	895.9	737.5	158.4
R6	-1111.68 anchor with the Kleykamp Li_2SiO_3 C_p	893.9	735.8	158.1
R6	Li_2CO_3 anchors: NIST-JANAF for the NBS Tables	895.7	737.5	158.2
R6	Li_2SiO_3 anchors: NIST-JANAF for the NBS Tables	908.1	747.0	161.1
R6	R6 reaction anchor, Duan et al. 2013 PCCP Table 5 at 300 K	893.5	735.2	158.4
R17	Adopted sources and carbonate phases	527.6	438.3	89.3
R17	-1111.68 anchor with the Kleykamp Li_2SiO_3 C_p	528.8	438.8	90.0
R17	Li_2CO_3 anchors: NIST-JANAF for the NBS Tables	527.8	438.5	89.3
R17	Li_2SiO_3 anchors: NIST-JANAF for the NBS Tables	517.2	430.0	87.2
R17	R17 reaction anchor, Duan et al. 2013 PCCP Table 5 at 300 K	442.0	361.9	80.0

The relevant sources are Chase, *NIST-JANAF Thermochemical Tables*, 4th edn (1998), $MgCO_3$, printed p. 627 (JANAF entry); Koziol and Newton (1995), *American Mineralogist*, **80**, 1252–1260, Tables 4–5 (doi:10.2138/am-1995-11-1214); Robie and Hemingway (1995), USGS Bulletin 2131; and the Chernosky–Berman assessment compared by Koziol and Newton. The direct magnesite Gibbs-energy comparison is Bénézeth et al. (2011), *Chemical Geology*, **286**, 21–31 (doi:10.1016/j.chemgeo.2011.04.016). For the heat-capacity alternative, see Kleykamp (1994), *Thermochimica Acta*, **237**, 1–12 (doi:10.1016/0040-6031(94)85178-6). The R7 pair is from Duan (2011), *Journal of Renewable and Sustainable Energy*, **3**, 013102, Table VI (doi:10.1063/1.3529427); its footnote a supplies the HSC-Chemistry comparison pair. The R6 and R17 reaction alternatives are from Duan, Pfeiffer, Li, Romero-Ibarra, Sorescu, Luebke and Halley (2013), *Phys. Chem. Chem. Phys.*, **15**, 13538, Table 5 at 300 K (doi:10.1039/c3cp51659h).

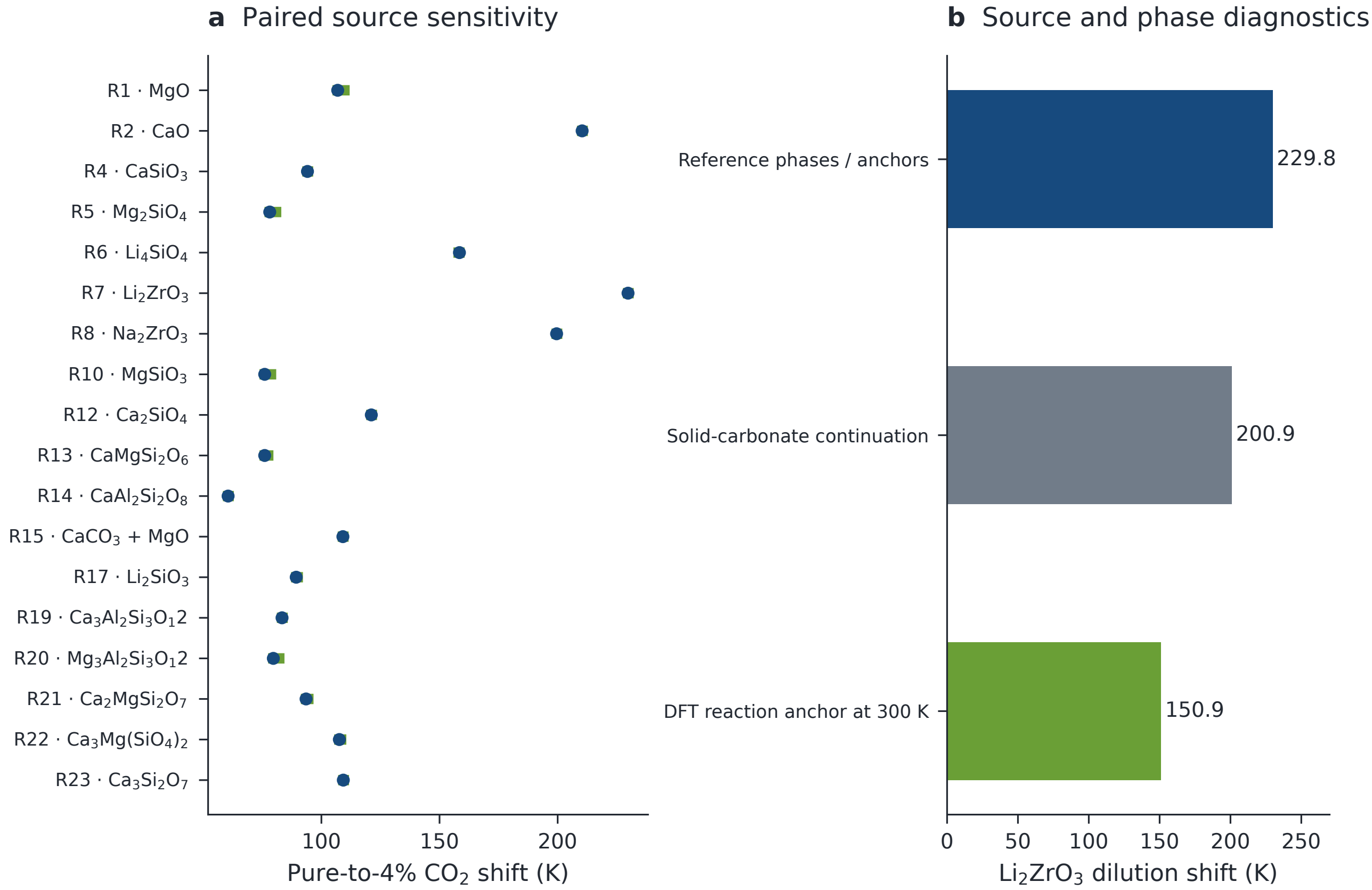

Fig. S6 Conditional source and phase sensitivities of the paired pure/4% dilution shift at 1 bar. (a) Navy circles show the adopted-input result for each Class I reaction. Green intervals span the specified magnesite literature anchors with the Kleykamp heat-capacity alternative; intervals can be smaller than the marker. This panel does not show the broader JANAF endpoint stress. (b) R7 shifts under the adopted anchors and phases (navy), metastable solid-carbonate continuation (gray), and Duan's 300 K reaction anchors with the present heat capacities (green). The source and phase cases are deterministic alternatives, not confidence intervals. Full values, including the separate JANAF stress, are given in Tables S7.3–S7.4 and the paired-source CSV.

S7.4 Representative gas-fugacity sensitivity

The Peng–Robinson (PR) equation of state (EOS) of 1976 is used as a gas-phase sensitivity, with classical quadratic attraction mixing, linear covolume mixing and $k_{ij} = 0$:

$$a_i(T) = 0.45724\frac{R^2T_{c,i}^2}{P_{c,i}}\left[1+\kappa_i(1-\sqrt{T/T_{c,i}})\right]^2, \qquad b_i = 0.07780\frac{RT_{c,i}}{P_{c,i}}, \tag{5}$$

$$\kappa_i = 0.37464 + 1.54226\omega_i - 0.26992\omega_i^2, \qquad a_m = \sum_i\sum_j y_i y_j \sqrt{a_i a_j}, \quad b_m = \sum_i y_i b_i. \tag{6}$$

Here $R = 0.08314462618$ L bar mol^{-1} K^{-1} for the EOS coefficients. With $A = a_mP/(R^2T^2)$ and $B = b_mP/(RT)$, the largest real root satisfying $Z > B$ is selected from

$$Z^3-(1-B)Z^2+(A-3B^2-2B)Z-(AB-B^2-B^3) = 0. \tag{7}$$

The fugacity coefficient is

$$\ln\phi_i = \frac{b_i}{b_m}(Z-1) - \ln(Z-B) - \frac{A}{2\sqrt{2}B}\left[\frac{2\sum_j y_j\sqrt{a_ia_j}}{a_m} - \frac{b_i}{b_m}\right]\ln\left[\frac{Z+(1+\sqrt{2})B}{Z+(1-\sqrt{2})B}\right]. \tag{8}$$

The reaction contribution $RT\sum_i \nu_i \ln\phi_i$ is added to eqn (2). Fugacity coefficients use the actual gas composition at the target extent and the total pressure, not the inlet CO_2 partial pressure. The nitrogen standard-state term is extent-independent, but nitrogen mixing and its effect on ϕ_i are included. Pure condensed-phase pressure corrections are held fixed; this test isolates gas nonideality. The equation is from Peng and Robinson (1976), *Industrial & Engineering Chemistry Fundamentals*, **15**, 59–64 (doi:10.1021/i160057a011). The component constants are specified in Table S7.5.

Table S7.5 Critical constants and acentric factors used in the PR gas sensitivity. Constants follow the component records in the CoolProp fluid library; pressure is converted to bar before forming the dimensionless EOS coefficients.

Component	T_c / K	P_c / bar	ω
CO_2	304.1282	73.773	0.22394
N_2	126.192	33.958	0.0372
H_2O	647.096	220.64	0.3442920843

The component records are available in the CoolProp fluid database; the PR calculation uses the constants above, rather than invoking CoolProp's multiparameter mixture model. The evaluated dry-gas set contains 45 roots: R1, R2, R6, R7 and R8, each at initial CO_2 fractions 1.00, 0.40 and 0.15 and total pressures 0.15, 1 and 20 bar. These pressures differ from the two-pressure full target grid in Section S7.1. Across these roots, ϕ_{CO_2} ranges from 0.999925 to 1.005201. The maximum absolute T_{50} change is 1.655 K, or 0.848 K when both ideal and PR roots lie inside the common heat-capacity interval. These are results at the selected target conditions, not a uniform error bound for all temperatures, mixtures or pressures up to 20 bar.

Table S7.6 Dry-gas PR sensitivity by reaction. $\Delta T = T_{50,\mathrm{PR}} - T_{50,\mathrm{ideal}}$; the final column includes only pairs whose two roots are within the heat-capacity interval. All 45 individual comparisons, fugacity coefficients and flags are supplied in the corresponding CSV.

Reaction	n	Min. ΔT / K	Max. ΔT / K	n_{IR}	Max. $\lvert\Delta T\rvert_{\mathrm{IR}}$ / K
R1	9	-0.002	+0.102	9	0.102
R2	9	+0.002	+0.442	7	0.357

Reaction	n	Min. ΔT / K	Max. ΔT / K	n_{IR}	Max. $\|\Delta T\|_{IR}$ / K
R6	9	+0.001	+0.535	9	0.535
R7	9	+0.001	+1.655	8	0.848
R8	9	+0.001	+0.731	8	0.549

Four additional humid-gas cases use R9 and R24, actual inlet fractions $(y_{CO_2}, y_{H_2O}, y_{N_2}) = (0.15, 0.30, 0.55)$ and 1 or 20 bar. They are explicitly uncalibrated $k_{ij} = 0$ sensitivities: water association and binary interaction parameters are not fitted. The CO_2/H_2O binary is the pair for which that approximation is worst, so these four rows should be read as an order-of-magnitude bound on the size of a gas-phase correction and not as a calibrated humid-mixture result. The dry-gas cases, where $k_{ij} = 0$ for CO_2/N_2 is a standard and defensible choice, carry the fugacity conclusion of the article. The selected target states remain below the pure-water saturation-pressure screen, but that check is not a complete mixture dew-point calculation. Both R24 temperatures lie below the adopted 500 K water heat-capacity limit. The R9 PR correction breaks the exact ideal-gas pressure cancellation even though its stoichiometric gas-mole change remains zero.

Table S7.7 Humid-gas PR sensitivity, using the matched initial composition stated above. L marks heat-capacity extrapolation. These four cases are not validated humid-mixture equilibrium predictions.

Reaction	P/bar	Ideal T_{50}/K	PR T_{50}/K	ΔT/K	ϕ_{CO_2}	ϕ_{H_2O}
R9	1	896.9	896.9	+0.088	1.000185	0.999676
R9	20	896.9	898.6	+1.755	1.003762	0.993688
R24	1	394.2^{L}	394.1^{L}	-0.072	0.998026	0.995046
R24	20	457.8^{L}	456.6^{L}	-1.232	0.978567	0.938115

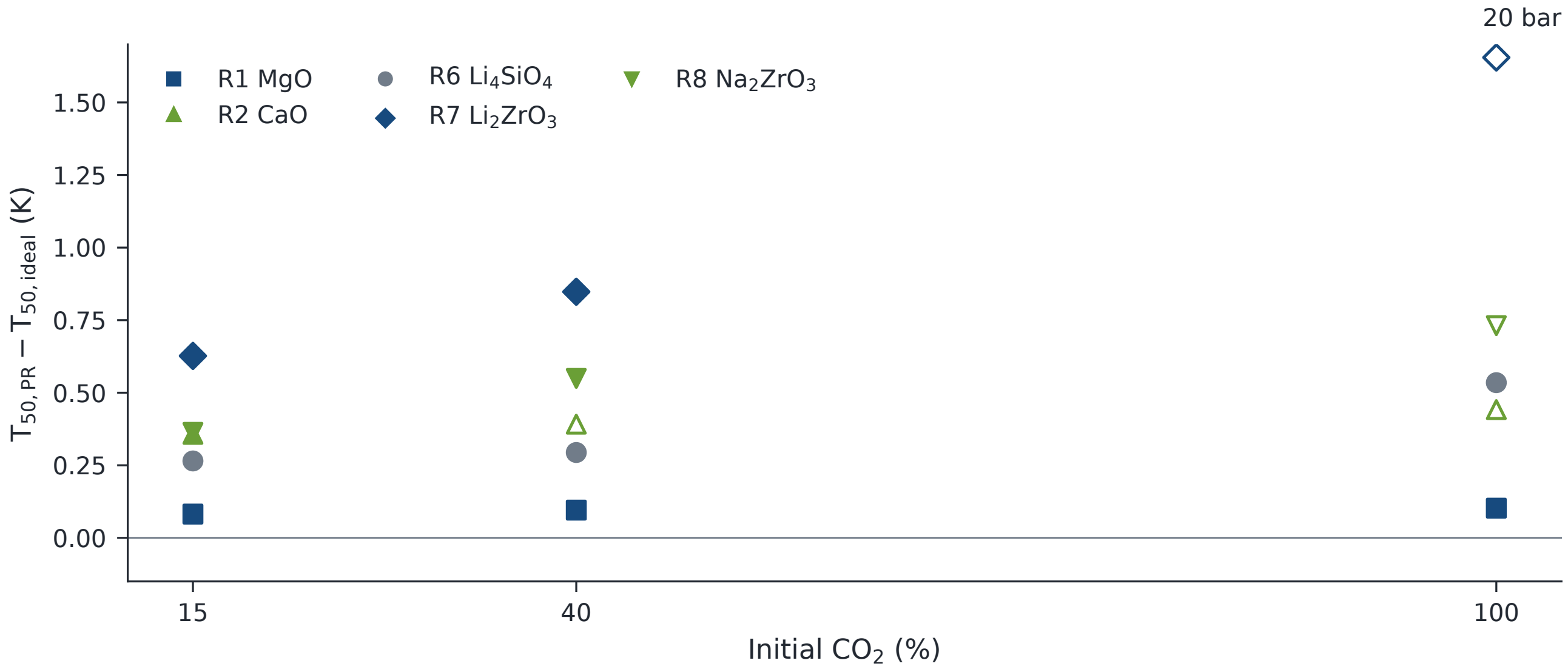

Fig. S7 Dry-gas fugacity sensitivity at 20 bar, shown for three initial CO_2 fractions and five reactions. Filled markers indicate that both the ideal and PR roots are within the common heat-capacity interval; open markers indicate that at least one root lies outside it. PR uses the instantaneous CO_2/N_2 composition, classical mixing and $k_{ij} = 0$. The full 45-case set also includes 0.15 and 1 bar (Table S7.6 and CSV). Humid cases are reported separately in Table S7.7 as uncalibrated water-mixture sensitivities.

S7.5 Numerical verification and supplementary data key

Target roots were bracketed over 300–2000 K with phase boundaries included in the scan and solved by Brent's method with an absolute temperature tolerance of 10^{-8} K. No multiple roots were located in the 460-row grid. Numerical tolerances govern reproducibility of the stated equations, not thermochemical precision; displayed target temperatures are rounded to 0.1 K. Independent total-Gibbs-energy minimization including all gas residual terms agrees with the nondegenerate dry-gas PR stationary roots within 2.3×10^{-7} in conversion. This quantity is unrelated to the 2.16×10^{-7} affinity-reconstruction maximum of Table S4.1, which concerns the ideal-gas sweep. Pure-feed degenerate boundaries are excluded from that conversion-difference check. The PR ideal-gas limit and an independent implementation in SI units were also checked. The calculation environment used Python 3.10.12, NumPy 2.2.6 and SciPy 1.15.3. The gas constant adopted in the equilibrium and target calculations is $R = 8.314$ J mol^{-1} K^{-1}. The dimensionless EOS coefficients of Section S7.4 use $R = 0.08314462618$ L bar mol^{-1} K^{-1}, so the two constants differ in the fifth significant figure. Recomputing the equilibrium roots with the CODATA value throughout changes no tabulated temperature by more than 0.07 K, the largest difference being 0.062 K at the 20 bar R7 root. A small number of tabulated values shift by one unit in the last printed digit. The ratios of Table 2A of the article inherit the same dependence, to about 0.08%. The value is stated here so that an independent recomputation using a library constant can account for differences of this size.

The supplementary CSV files contain numerical inputs and outputs, with one row per stated case:

- `Table_S7_1_target_conversion_grid.csv`: 460 target rows; nominal and actual inlet fractions, total pressure, target conversion, temperature, feasible maximum, root count and validity status.
- `Table_S7_2_matched_humidity.csv`: 50 target rows; actual $CO_2/H_2O/N_2$ fractions, pressure, feasible maximum, target temperature and status.
- `Table_S7_3_paired_source_sensitivity.csv`: 414 paired source/phase rows; scenario, reaction, both target temperatures, their difference and separate endpoint validity flags. Scenario names correspond to the 23 cases in Table S7.3.

- `Table_S7_equilibrium_water_ratios.csv`: six equilibrium water-to-CO_2 ratios for R3 and R9 at 500, 700 and 900 K.
- `Table_S7_6_PR_dry.csv`: 45 dry-gas comparisons; feed, total pressure, ideal/PR roots, temperature difference, CO_2 fugacity coefficient, compressibility and validity flags.
- `Table_S7_7_PR_humid.csv`: four humid-gas comparisons; the matched feed, ideal/PR roots, both reactive-gas fugacity coefficients and explicit gas-phase and heat-capacity qualifications.

All pressures are in bar, temperatures in kelvin and gas fractions on a molar basis. Blank target temperatures signify the stated no-root or feed-limit status, never zero temperature. The heat-capacity flags identify the intersection of the species data ranges and must accompany quoted extrapolated values. None of these numerical verification or source-sensitivity checks assigns a probability that the full physical model is correct.

S7.8 Primary screening population and provisional cases

R6 and R7 depend on three unlocated reference-state entries and are excluded from the primary aggregate. Individual calculations are retained unchanged for inspection and sensitivity analysis. Table S7.8 reports the primary statistics; `Table_S7_8_screening_scope.csv` gives both populations for all 23 scenarios. The 100.5 K median in the full-set tables refers to 18 reactions including these two provisional cases. Uncertainty of a population median cannot be inferred from the incomplete single-reaction uncertainty contributions.

Table S7.8. Dilution shifts for the 16 Class I reactions with located reference-state sources. The comparison is between the pure-feed step boundary and the 4% initial-CO_2 50% conversion boundary at 1 bar. R6 and R7 are excluded. Values in kelvin.

Source scenario	Median	Maximum
Reference	93.8	210.4
Koziol–Newton magnesite anchor	93.8	210.4
Robie–Hemingway magnesite anchor	94.1	210.4
Chernosky–Berman magnesite anchor	94.3	210.4
JANAF lower enthalpy endpoint	95.2	210.4
JANAF upper enthalpy endpoint	92.4	210.4
